# Fluoro-Aryl Substituted α,β^2,3^-Peptides in the Development of Foldameric Antiparallel β-Sheets: A Conformational Study

**DOI:** 10.3389/fchem.2019.00192

**Published:** 2019-04-02

**Authors:** Raffaella Bucci, Alessandro Contini, Francesca Clerici, Egle Maria Beccalli, Fernando Formaggio, Irene Maffucci, Sara Pellegrino, Maria Luisa Gelmi

**Affiliations:** ^1^Department of Pharmaceutical Sciences (DISFARM), University of Milan, Milan, Italy; ^2^Department of Chemistry, University of Padova, Padova, Italy; ^3^CNRS UMR 7025, Génie Enzymatique et Cellulaire, Centre de Recherche de Royallieu, Compiègne, France; ^4^Génie Enzymatique et Cellulaire, Centre de Recherche de Royallieu, Sorbonne Universités, Université de Technologie de Compiègne, Compiègne, France

**Keywords:** β^2,3^-diaryl-amino acid, α, β^2,3^-peptide, extended peptide, antiparallel β-sheet, conformational analyses, foldamers

## Abstract

α,β^2,3^-Disteroisomeric foldamers of general formula Boc(*S-*Ala-β-2*R*,3*R-*Fpg)_n_OMe or Boc(*S-*Ala-β-2*S*,3*S-*Fpg)_n_OMe were prepared from both enantiomers of *syn* H-2-(2-F-Phe)-h-PheGly-OH (named β-Fpg) and *S-*alanine. Our peptides show two appealing features for biomedical applications: the presence of fluorine, attractive for non-covalent interactions, and aryl groups, crucial for π-stacking. A conformational study was performed, using IR, NMR and computational studies of diastereoisomeric tetra- and hexapeptides containing the β^2,3^-amino acid in the *R,R*- and *S,S*-stereochemistry, respectively. We found that the stability of peptide conformation is dependent on the stereochemistry of the β-amino acid. Combining *S*-Ala with β-2*R,3R*-Fpg, a stable extended β-strand conformation was obtained. Furthermore, β-2*R,3R*-Fpg containing hexapeptide self-assembles to form antiparallel β-sheet structure stabilized by intermolecular H-bonds and π,π-interactions. These features make peptides containing the β^2,3^-fluoro amino acid very appealing for the development of bioactive proteolytically stable foldameric β-sheets as modulators of protein-protein interaction (PPI).

## Introduction

Amino acids are small molecules able to induce high molecular complexity, promoting discrete three-dimensional folded structures in peptides. α-Amino acid-based peptides show some drawbacks such as the low stability to proteases, and, in the case of short peptides, the lack of a stable secondary structure. The use of oligomers with unnatural backbones is a powerful strategy to overcome those issues, while maintaining the desired fragment structure.

In particular, β-peptide foldamers (Seebach et al., 1996, 2004; Gellman, 1998) have been extensively studied in the last two decades. These molecules can adopt specific compact conformations despite the increased conformational space due to the additional methylene group of the β-amino acid. Furthermore, their substitution pattern can influence the conformation of β-peptides, as reported in several experimental (Cheng et al., 2001; Seebach et al., 2006; Seebach and Gardiner, 2008; Pilsl and Reiser, 2011; Vasudev et al., 2011; Berlicki et al., 2012; Johnson and Gellman, 2013; Basuroy et al., 2014; Lee et al., 2014; Wang and Schepartz, 2016; Bucci et al., 2017a) and molecular modeling studies (Wu et al., 2008; Zhu et al., 2010; Baldauf and Hofmann, 2012). An even larger pool of secondary structure motifs could be obtained, by the combination of α- and both acyclic and cyclic β-amino acids (αβ; α*αβ*; α*ββ*; etc). Inspired by the diversity of folded structures and functions manifested by peptides and proteins, β-peptide mimics and foldamers have been thus exploited for different applications ranging from biomedicine (Horne, 2011; Cabrele et al., 2014; Checco and Gellman, 2016) to material science (Gopalan et al., 2015; Clerici et al., 2016; Del Borgo et al., 2017).

Only few examples are reported related to α,β-repeating sequences containing acyclic amino acids (Sharma et al., 2005; Srinivasulu et al., 2006; Angelici et al., 2007; Balamurugan and Muraleedharan, 2012; Basuroy et al., 2014), the majority of them giving helix constructs.

Our interest toward the preparation of non-natural amino acids (Pellegrino et al., 2008; Penso et al., 2012; Ruffoni et al., 2015) and their use for peptidomimetic synthesis for different applications (Pellegrino et al., 2015, 2016; Ruffoni et al., 2016; Bucci et al., 2018, 2019; Tonali et al., 2018) is well-documented. Recently, our research group reported on a diastreoselective synthesis of a new class of β-amino acids, *syn*-S^*^, S^*^-β^2,3^-diarylamino acids, differently substituted on the aromatic ring (Bonetti et al., 2014).

**Figure 1:**
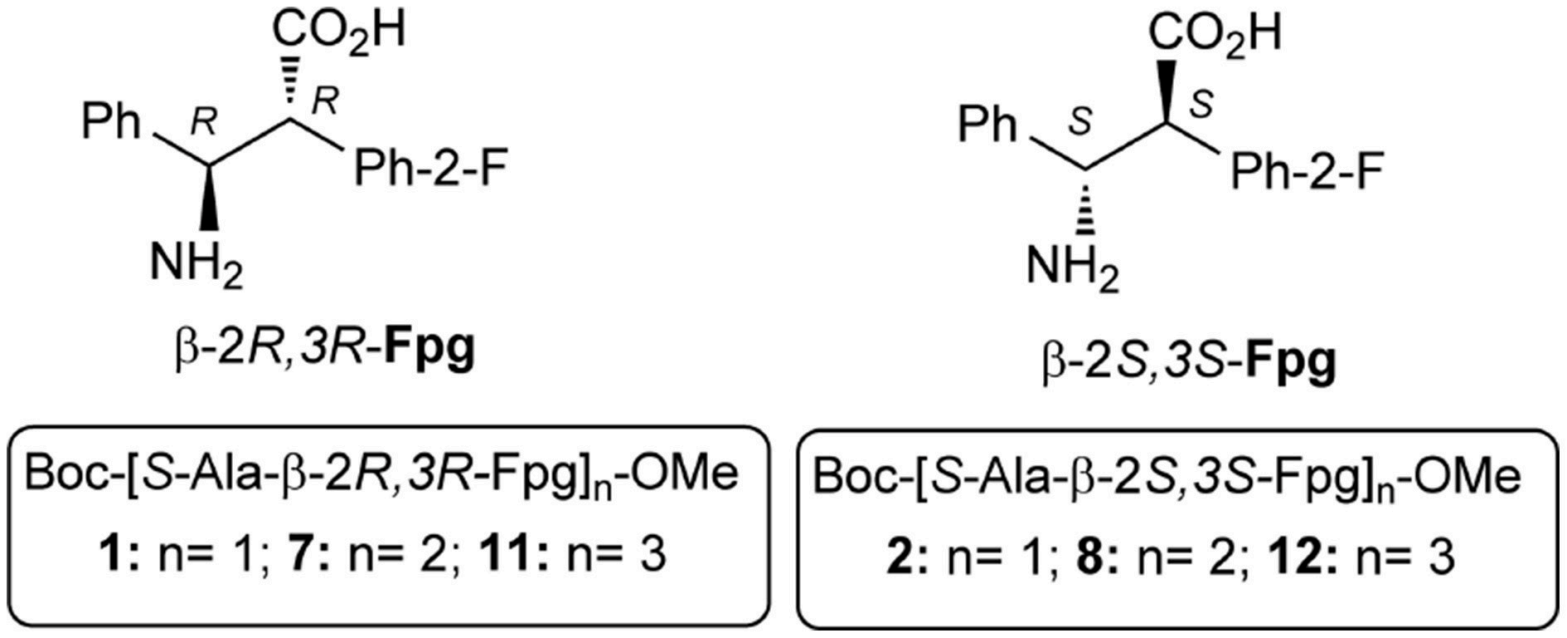
Di-, tetra-, and hexa-peptides from *S*-Ala and β-Fpg.

Due to the lack of information related to α,β^2,3^-amino acids repeating sequences, here we report on the preparation and conformational studies of α,β^2,3^-peptides, containing *syn* H-2-(2-F-Phe)-h-PheGly-OH, named β-Fpg, and helicogenic *S*-alanine (Figure 1). This scaffold shows two features that can be appealing for biomedical applications: the presence of a fluorine, useful for non-covalent interactions, and two aryl groups, crucial for π-stacking and hydrophobic interactions. Taking advantage of the use of fluorine-substituted β-2*S,3S*-Fpg (Figure 1), in combination with *S*-Ala or *S*-Arg-*S*-Ala, we recently prepared short peptides able to generate proteolytically stable nanotubes (Bonetti et al., 2015) and spherical aggregates (Bucci et al., 2017b) as drug delivery systems.

Here we report on a conformational study on diastereoisomeric tetra- and hexapeptides α,β^2,3^-foldamers of general formula Boc(*S-*Ala-β-2*R,3R*-Fpg)_n_OMe and Boc(*S-*Ala-β-2*S,3S*-Fpg)_n_OMe (Figure 1).

Our results showed that the stability of peptide conformation is dependent on the stereochemistry of the β-amino acid. Combining *S*-Ala with β-2*R,3R*-Fpg, a stable extended β-strand conformation was obtained. Moreover, it was found that hexapeptide **11** (Figure 1) self-assembles to form antiparallel β-sheet structure stabilized by intermolecular H-bonds and π,π-interactions. This result is important since, to the best of our knowledge, no examples of extended α,β^2,3^-foldamers are reported. Furthermore, there is a need of the development of bioactive proteolytically stable foldameric β-sheets that might be useful as modulators of protein-protein interactions (PPI) (Hegedüs et al., 2016). The design of this secondary structure is indeed not trivial, although β-sheet interfaces frequently occurred in PPI.

## Materials and Methods

### Chemistry

Experimental procedures, compound characterization data for newly synthesized peptides (**3-5** and **7-12**; for ^1^H, ^13^C, and ^19^F NMR spectra see Figures S10–S39) are reported in the Supplementary Material. Dipeptide **1**, **2** (Bonetti et al., 2015) and **6** (Bucci et al., 2017b) are known compounds.

Melting points were determined with a Stuart Scientific melting point apparatus in open capillary tubes and are uncorrected. Chemicals were purchased from Sigma Aldrich and were used without further purification. ESI MS were recorded on a LCQ Advantage spectrometer from Thermo Finningan and a LCQ Fleet spectrometer from Thermo Scientific. The FT-IR absorption spectra were recorded with a Perkin-Elmer 1720X spectrophotometer, nitrogen flushed, equipped with a sample-shuttle device, at 2 cm^−1^ nominal resolution, averaging 100 scans. Solvent (base-line) spectra were recorded under the same conditions. Cells with path lengths of 1.0 and 10 mm (with CaF_2_ windows) were used. Spectrograde CDCl_3_ (99.8% D) was purchased from Fluka. The NMR spectroscopic experiments were carried out either on a Varian MERCURY 200 MHz (200 and 50 MHz for ^1^H and ^13^C, respectively), Varian MERCURY 300 MHz (300, 75, 282 MHz for ^1^H, ^13^C, and ^19^F, respectively), or Bruker Avance I 500 MHz spectrometers (500 and 125 MHz for ^1^H and ^13^C, respectively). Chemical shifts (δ) are given in ppm relative to the CHCl_3_ internal standard, and the coupling constants *J* are reported in Hertz (Hz). Optical rotations were measured on a Perkin–Elmer 343 polarimeter at 20°C (concentration in g/100 mL).

### Computational Methods

The non-natural β-amino acids, capped respectively with an acetyl (Ac) and an OMe group at the *N*- and *C*-termini, were designed using MOE (Molecular Operating Environment, 2013) and submitted to a “low mode” conformational search (MMFF94x force field, Born solvation, iteration limit = 40 000, MM iteration limit = 2,500, rejection limit = 500). The two lowest energy conformations having ϕ, θ, and ψ dihedrals corresponding to two different secondary structures, namely the extended one and a β-turn, were selected to derive partial charges with the R.E.D.III.52 software (Dupradeau et al., 2010). Each geometry was optimized at the HF/6-31G(d) level, and two different spatial orientations were used to derive orientation- and conformation-independent RESP-A1 charges.

The tetra- (**m7** and **m8**) and hexapeptides (**m11** and **m12**) of general formula 8 were built with the *tleap* module of Amber14 (Case et al., 2014) by imposing an extended conformation (ϕ = ψ = ω = θ = 180°), and solvated with an octahedral box of CHCl_3_. All the systems were consequently submitted to a preliminary equilibration with the pmemd module of Amber14 package, using the ff14SB (Maier et al., 2015) force field. In detail, the systems were relaxed by minimizing hydrogens and solvent (2,000 cycles of steepest descent and 5,000 cycles of conjugated gradient). The solvent box was equilibrated at 300 K by 100 ps of NVT and 100 ps of NPT simulation using a Langevin thermostat with a collision frequency of 2.0 ps^−1^. Successively, a minimization of side chains and CHCl_3_ with restraints on backbone atoms of 25 kcal/mol and a total minimization (2,500 cycles of steepest descent and 5,000 cycles of conjugated gradient) were performed. The systems were then heated up to 300 K in 6 steps of 5 ps each (ΔT = 50 K), where backbone restraints were reduced from 10.0 to 5 kcal/mol. Full equilibration was performed in the NVT ensemble (100 ps, backbone restraints = 5.0 kcal/mol) and in the NPT ensemble (1 step of 200 ps, backbone restraints = 5 kcal/mol; 3 steps of 100 ps each, reducing the backbone restraints from 5.0 to 1.0 kcal/mol, and 1 step 1 ns with 1.0 kcal/mol of nd backbone restraints). Finally, unrestrained production runs were run at 300 K for 15 ns. An electrostatic cutoff of 8.0 Å was applied to all the calculations. The equilibrated geometries and the average dihedral energies and potential obtained through the accurate systems equilibrations were used as starting point for accelerated MD (aMD) simulations, where the α value was set to 0.2 and 0.16, for the dihedral and potential factors, respectively.

The aMD simulations of each peptide were run until convergence, for a total of 500 ns for the tetrapeptides **m7** and **m8**, and 800 ns for hexapeptides **m11** and **m12**, respectively. The root mean square displacement of backbone heavy atoms from the extended conformation was used as a metric of convergence (Figure S1). All the analyses were conducted on the last 200 ns of trajectory. Cluster analyses were performed with the *cpptraj* module of Amber14 by sampling one of every 8 frames, using the average-linkage algorithm and requesting ten clusters; the pairwise mass-weighted RMSD on backbone Cα atoms was used as a metric. H-bonds were computed with cpptraj by setting a donor-acceptor distance threshold of 4.0 Å and an angle cutoff of 110°. Only H-bonds with an occupancy >20% were considered. *Cpptraj* was used also for computing the radius of gyration of investigated peptides.

## Results and Discussion

### Synthesis

**Scheme 1:**
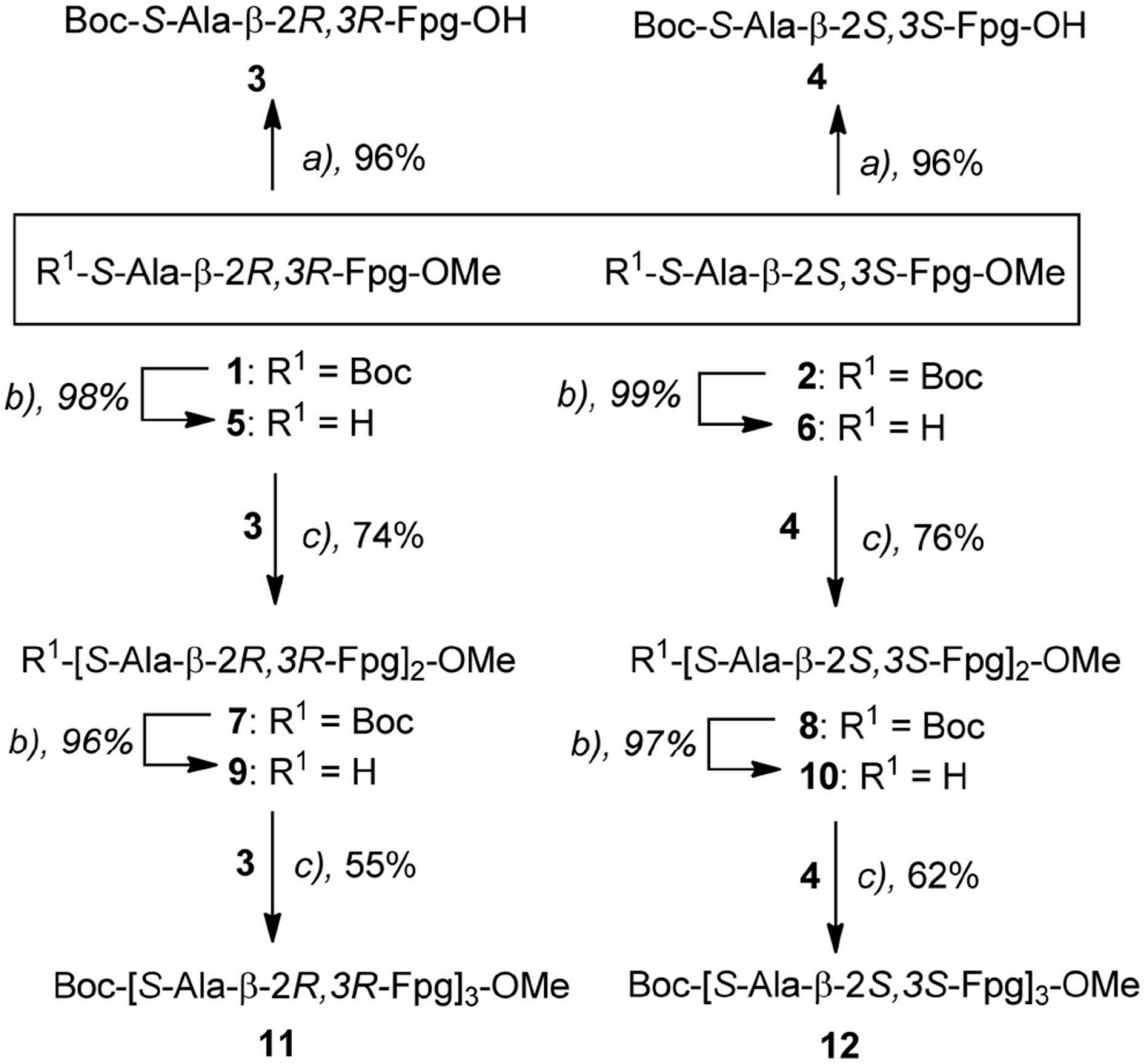
*a*) *i)* 1 M HCl, 80°C, 12 h; *ii)* (Boc_2_O, DCM, TEA, 25°C, 12 h; *b*) TFA, CH_2_Cl_2_, 0°C, 1 h; *c)* EDC (1.1 equiv.)/EtCN-oxime(1.1 equiv.), CH_2_Cl_2_, 25°C, 3 h.

Dipeptides **1** and **2** were efficiently synthesized in gram scale starting from a racemic mixture of amino acid β-Fpg, according to a known procedure (Bonetti et al., 2015). Pure dipeptides were used for the preparation of α,β^2,3^-tetra- and hexa-peptide sequences (Scheme 1).

The deprotection of the carboxyl group of dipeptides **1** and **2** with LiOH or KOH gave a partial epimerization of the benzylic-C_α_ position of the β-amino acid, even at low temperature. To avoid this problem, we moved to the deprotection of both *C*- and *N*-termini operating in 1 M HCl (80 °C, 12 h, 99%), followed by Boc-protection of *N-*terminu*s* (Boc_2_O, DCM, TEA, 25 °C, 12 h, 96%). Dipeptide **3** and **4** were obtained in excellent overall yields. The deprotection of nitrogen atom was performed in standard conditions (TFA in CH_2_Cl_2_, 0°C, 1 h) affording dipeptides **5** and **6** from **1** and **2** (99%), respectively. Good yields in the coupling reactions between **3** with **5** and **4** with **6** affording **7** (74%) and **8** (76%), respectively, were achieved using EDC (1.1 equiv.)/EtCN-oxime/(1.1 equiv.) in CH_2_Cl_2_ (25°C, 3h).

Using similar *N*-deprotection/condensation protocols, hexapeptides **11** and **12** were prepared, *via* esters **9** and **10**.

### IR Analysis

In our FTIR absorption investigation we focused on the N-H stretching (amide A) region (3,470–3,240 cm^−1^). Absorptions in this region are highly dependent on H-bond formation and, consequently, they are conformationally informative. To get insights into the modes of folding and self-association we chose as solvent CDCl_3_. It has a relatively low polarity and it is known to support ordered secondary structures in peptides.

As the peptide chain is elongated, the relative intensity of the N-H stretching band at about 3,330 cm^−1^ markedly increases as compared to that of the band at about 3,430 cm^−1^ (Figure S9). The first band is assigned to H-bonded NH groups, while the second is due to free (solvated) NH groups (Palumbo et al., 1976; Toniolo et al., 1985).

**Figure 2:**
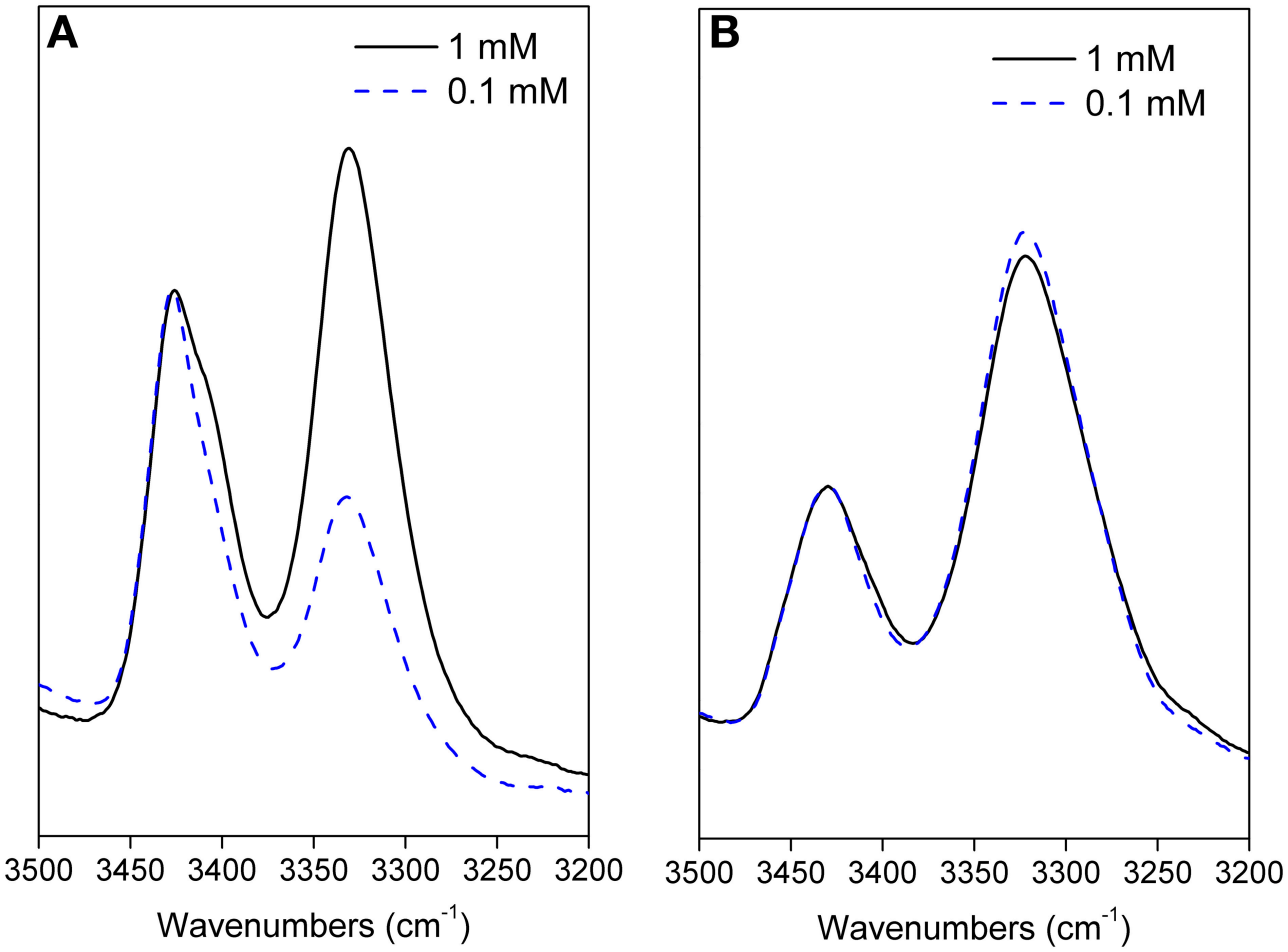
Amide A portion of the FTIR spectra acquired for **11 (A)** and **12 (B)** in CDCl_3_ solution at 0.1 and 1 mM peptide concentrations (cell path lengths of 10 and 1 mm were used, respectively).

In the absence of aggregation, this behavior can be ascribed to the formation of ordered secondary structures, stabilized by intramolecular H-bonds. From our analysis we conclude that the chirality of the two stereogenic centers of the fluoro amino acid remarkably affects the peptide 3D-structure. In particular, the maximum of the bonded N-H band is located at 3,320 cm^−1^ for peptide **12**, but at higher energy, 3,330 cm^−1^, for peptide **11** (Figure 2). This latter observation implies that the H-bonds in the **11** hexapeptide are weaker. Indeed, an inspection to the behavior of the two hexapeptides at two concentrations (0.1 and 1 mM) is highly informative (Figure 2). For peptide **12** the relative intensities of the bands centered at 3,430 cm^−1^ (free NHs) and at 3,320 cm^−1^ (H-bonded NHs) do not significantly change upon diluting 10 times (Figure 2B). Therefore, we assume that the H-bonded NHs originate mainly from intramolecular interactions in **12**. Conversely, a clear dilution effect is observed for **11** (Figure 2A) highlighting an important contribution from intermolecular H-bonds. The onset of a β-sheet conformation can thus be hypothesized for **11** based on this FTIR absorption analysis.

In conclusion, this IR absorption analysis indicates that the longer oligomers adopt secondary structures strongly dependent on the chirality of the fluoro-β^2,3^-diarylamino acid.

### NMR Characterization

All synthesized peptides were fully characterized by NMR (^1^H, ^13^C, COSY, TOCSY, HMBC, HMQC, NOESY, ROESY) in CDCl_3_. In many cases broad CH and NH signals were detected, thus preventing a clear information on spatial proximities. Detailed data are reported in the Supporting Material (**7**: Table TS1; **8**: Table TS2; **11**: Tables TS3, TS4; **12**: Table TS5). Finally, fluorine NMR was performed for all peptides. In all cases, fluorine atoms resonate in δ −118 - −117 region, except for fluorine of Ar_β2_ and Ar_β4_ of **11** that resonate at lower fields (δ −116.1, −114.8, broad signals; not assigned).

As reported in the literature (Cheng et al., 2001; Balamurugan and Muraleedharan, 2012; March et al., 2012), *J*_2,3_ and *J*_NH−CHβ_ values help to predict the secondary structure of peptides containing β-amino acids. Large values (10–12 Hz) indicate the antiperiplanar arrangement of C_α_/C_β_ substituents and of NH-CH_β_ and are consistent with an extended conformation. Smaller values (3–4 Hz) indicate a gauche conformation corresponding to turn/helix constructs. Since we started from a *syn*-amino acid, an extended conformation could be expected. On the other hand, the presence of helicogenic alanine could influence the secondary conformation of the peptide. As shown in Table 1, large *J*_2,3_ and *J*_NH−CHβ_ values were detected for peptides containing β-2*R,3R*-Fpg amino acid indicating their extended conformation. Lower *J* values were detected for peptides containing β-2*S,3S*-Fpg amino acid, mostly for *J*_2,3_ of amino acids at position 2 and 4 of peptide **12**. On the other hand, these *J* values are higher with respect to those reported for helix constructs containing β-amino acids (*J* 3–4 Hz), suggesting that for **12** more than one preferred conformation is present (Seebach et al., 1999).

**Table 1:** ^1^H NMR parameters for di- (**1**,**2**) tera- (**7**,**8**) and hexapeptides (**11**,**12**).

**Compd**.	**Amino acid**	**β-2*****R,3R*****-Fpg series**				**β-2*****S,3S*****-Fpg series**			
		**NH_**δ**_**	***J*_**NH-CHα**_**	***J*_**NH-CHβ**_**	***J*_**2,3**_**	**NH_**δ**_**	***J*_**NH-CHα**_**	***J*_**NH-CHβ**_**	***J*_**2,3**_**
**1** and **2**	Ala-1	5.38	brs	–	–	5.38	brs	–	–
	Beta-2	7.07	–	10.8	10.9	7.07	–	9.9	10.5
**7** and **8**	Ala-1	5.08	brs	–	–	4.83	brs	–	–
	Beta-2	6.99	–	^a^	11	7.37	–	9.0	8.3
	Ala-3	6.39	brs	–	–	6.36	brs	–	–
	Beta-4	6.52	–	10.3	10.7	6.69	–	8.4	10.3
**11** and **12**	Ala-1	5.49	6.8	–		5.12	brs	–	
	Beta-2	7.49	–	9.4	11.1	8.09	–	8.2	6.4
	Ala-3	7.43	^a^	–	–	7.56	brs	–	–
	Beta-4	6.88	–	10.7	11.4	8.18	–	9.7	6.0
	Ala-5	6.91	^a^	–	–	7.24	^a^	–	–
	Beta-6	6.76	–	11.2	10.6	6.58	–	8.6	9.4

a *Overlapped signals*.

A further main difference between the two series is the Me_Ala_ resonances. According to an extended conformation, Me_Ala3_ of **7** (see **Figure 7A**) is located between Ph_β2_ and Ar_Fβ4_ possessing the same orientation. This induces a strong shielding effect on the methyl group that resonates at higher field (δ 0.48). Similarly, **11** is characterized by two shielded methyl groups [Me_Ala3_ (δ 0.32); Me_Ala5_ (δ 0.35)], in agreement with an extended conformation (**Figure 8A**). Me_Ala3_ (δ 0.97) in **8**, as well as all three Me_Ala_ [Me_Ala1_(δ 1.36); Me_Ala3_ (δ 1.20); Me_Ala5_ (δ 1.01)] of **12**, not affected by this electronic effect, resonate at lower field. Furthermore, differences of NH chemical shift are mostly found for hexapeptides **11** and **12**, being the NH resonances of this last at lower field.

The temperature dependence experiments of the NH proton chemical shifts variation, giving insights on NH inaccessibility or on NH-bond network (Augspurger et al., 1995) were performed for peptides **7**, **8**, and **12** (NHs proximity to aromatic protons prevented a clear overview of their behavior in peptide **11**).

**Figure 3:**
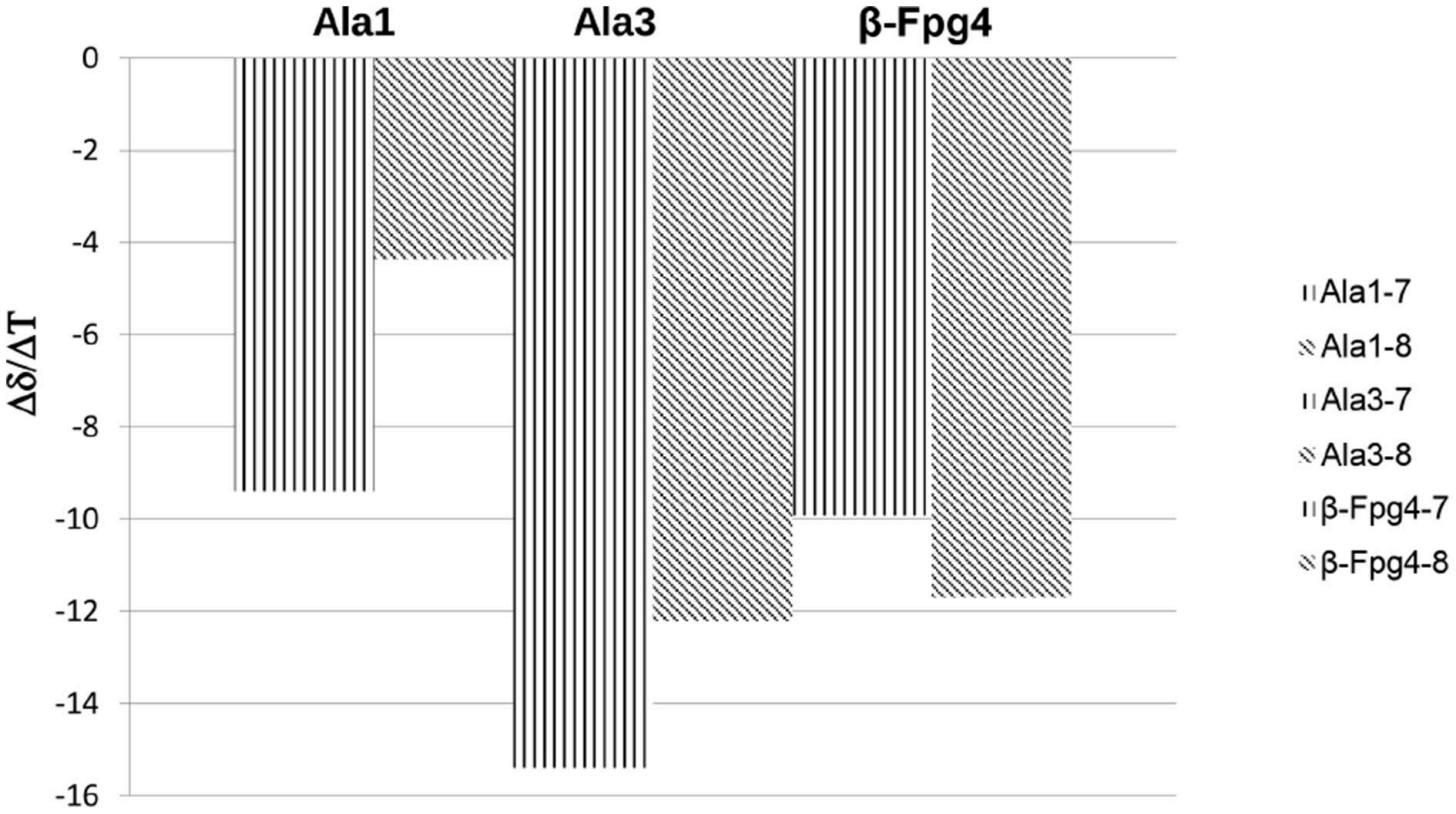
Δδ/ΔT NH values for peptides **7** and **8** (273–328 K).

Very high Δδ/ΔT values (>-9.4 ppb K^−1^; this value is not detectable for NH_β2_, overlapped signal) were found for **7** and **8**, indicating absence of H-bonds. As exception, a medium value (−4.3 ppb K^−1^) was detected for NH_Ala1_ of **8**, indicating an equilibrium between a H-bonded and non-bonded status or a shielded environmental (Figure 3).

These results agree with IR data indicating, for both peptides, a low intensity of the bond at about 3,320 cm^−1^ and a lower area for **7** with respect to **8** (Figure 2).

**Figure 4:**
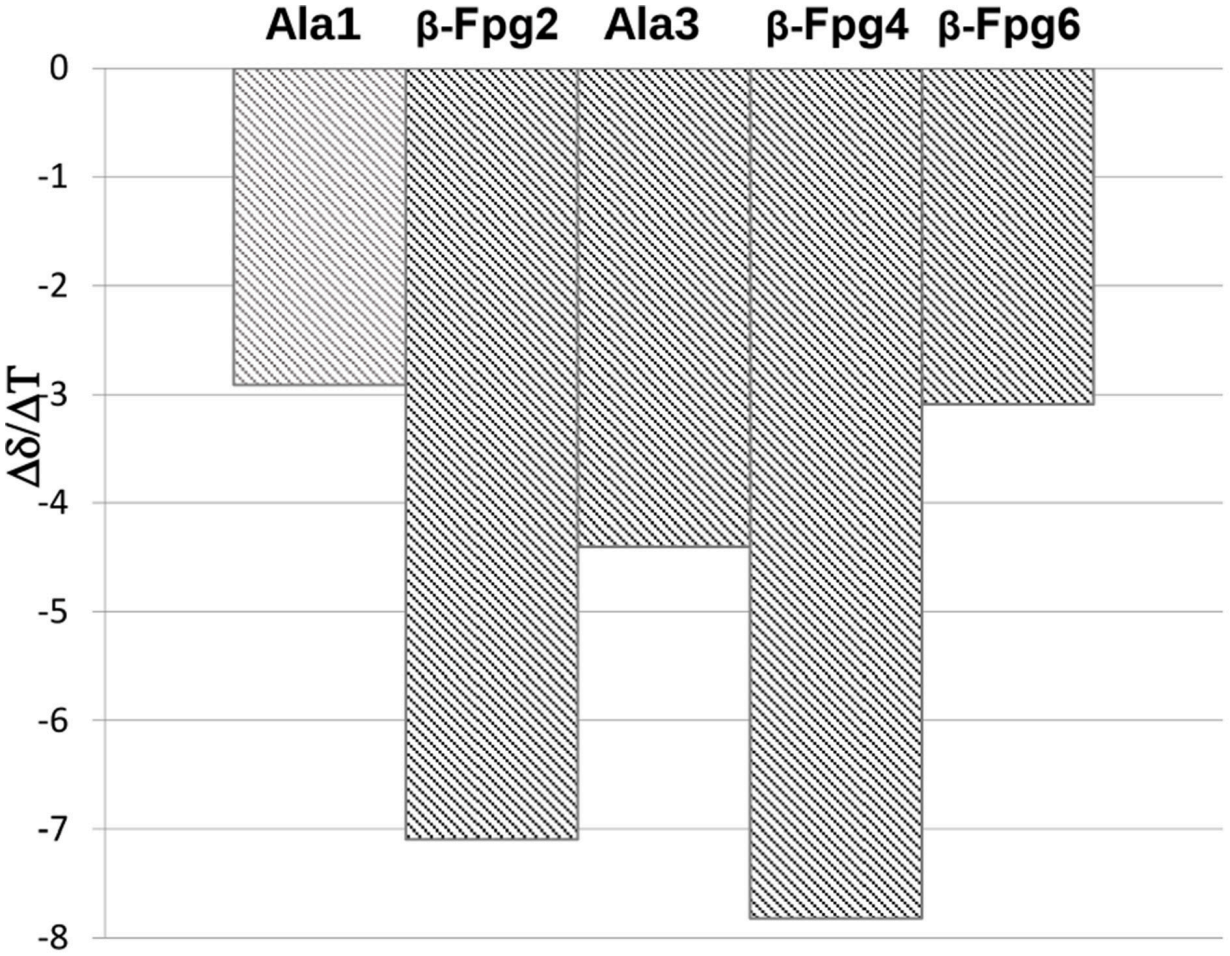
Δδ/ΔT NH values for peptides **12** (273–328 K).

Smaller Δδ/ΔT values were found for NHs of **12** with respect to **8**, mostly for NH_Ala1_, NH_β6_, and NH_Ala3_ (−2.9, −3.01, −4.4 ppb K^−1^, respectively; Figure 4). These data are consistent with IR analyses (Figure 2B) indicating the tendency of **12** to give strong/medium intramolecular C = O···H-N H-bonds.

**Figure 5:**
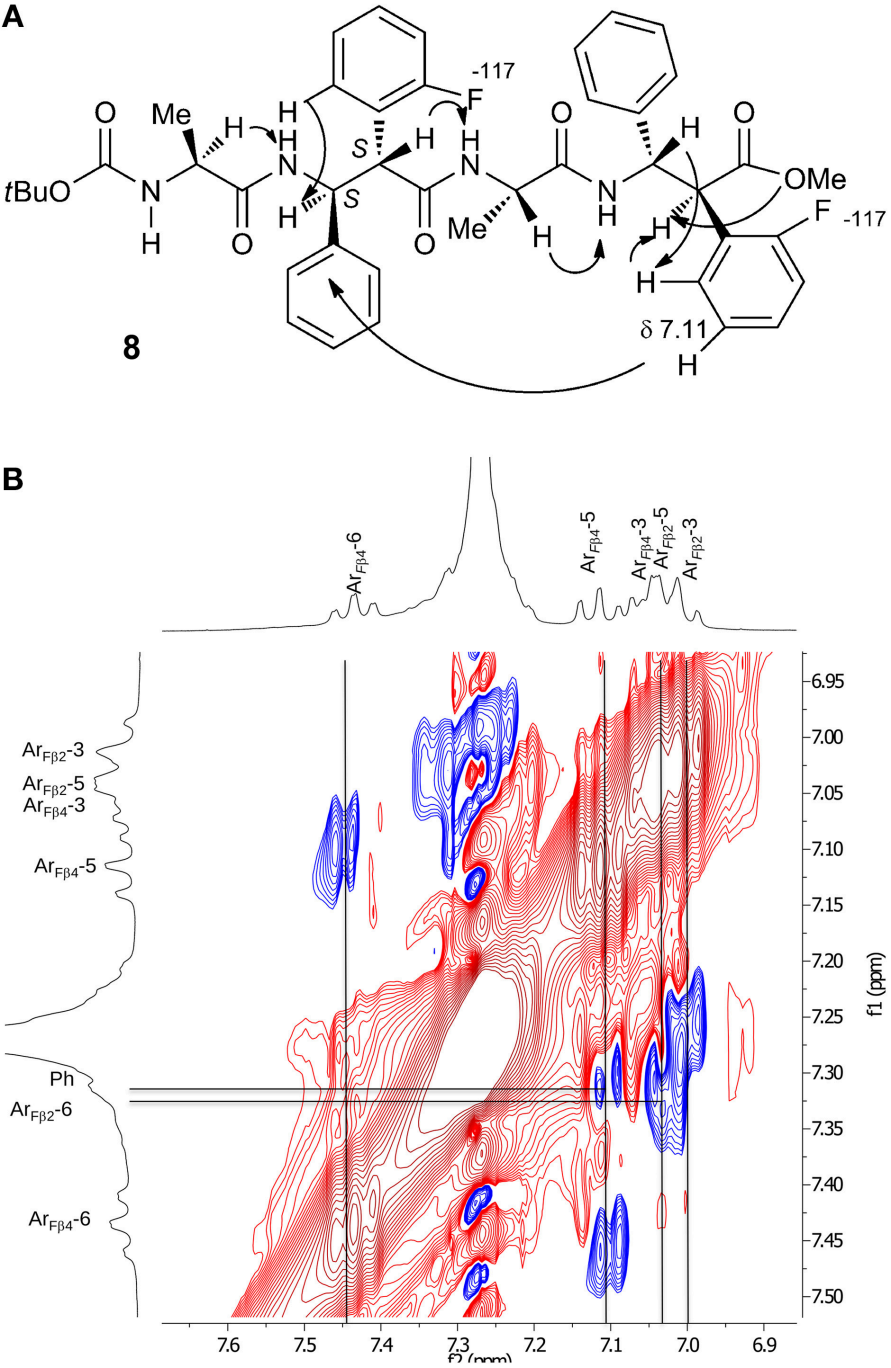
**(A)** NOEs of peptide **8**. **(B)** Zoom of aromatic region spectrum (CDCl_3_, 10 mM, 293 K, 400 MHz, 300 ms).

A complete set of CH_α_/NH_β_ (i, i+1) and H_β_-2/NH_α_ (i,i+1) NOEs characterizes peptides **7** (Figure S2), **8** (Figure S3), and **12** (Figure S8). A peculiar NOE for **8** is between H-5 of Ar_β4_ (δ 7.11) with Ph_β2_ (δ 7.31, Figure 5). The resonances of Ph protons in both Ph-3_β2_ and Ph-3_β4_ range in δ 7.31–7.22 region. On the other hand, the antiperiplanar position of Ph with Ar_*F*_ of β-4 amino acid (*J*_2,3_ 10.3 Hz) excludes that the detected spatial proximity between Ar_F_ and Ph derives from the aromatic moieties of the same amino acid. The formation of a turn in β-2/Ala3/β-4 region is suggested, as confirmed by computational data (**Figure 7C**), that is consistent with the deviation of *J*_NH−CHα_ values characterizing the extended peptides (Table 1).

Considering peptide **12**, apart the above-mentioned NOEs, only a weak spatial proximity was detected between NH-2 and NH-3 (Figure S8). Detection of possible NOEs between non-sequential Ph/Ar protons, as shown for **8**, were prevented because of the presence of several overlapped aryl protons.

**Figure 6:**
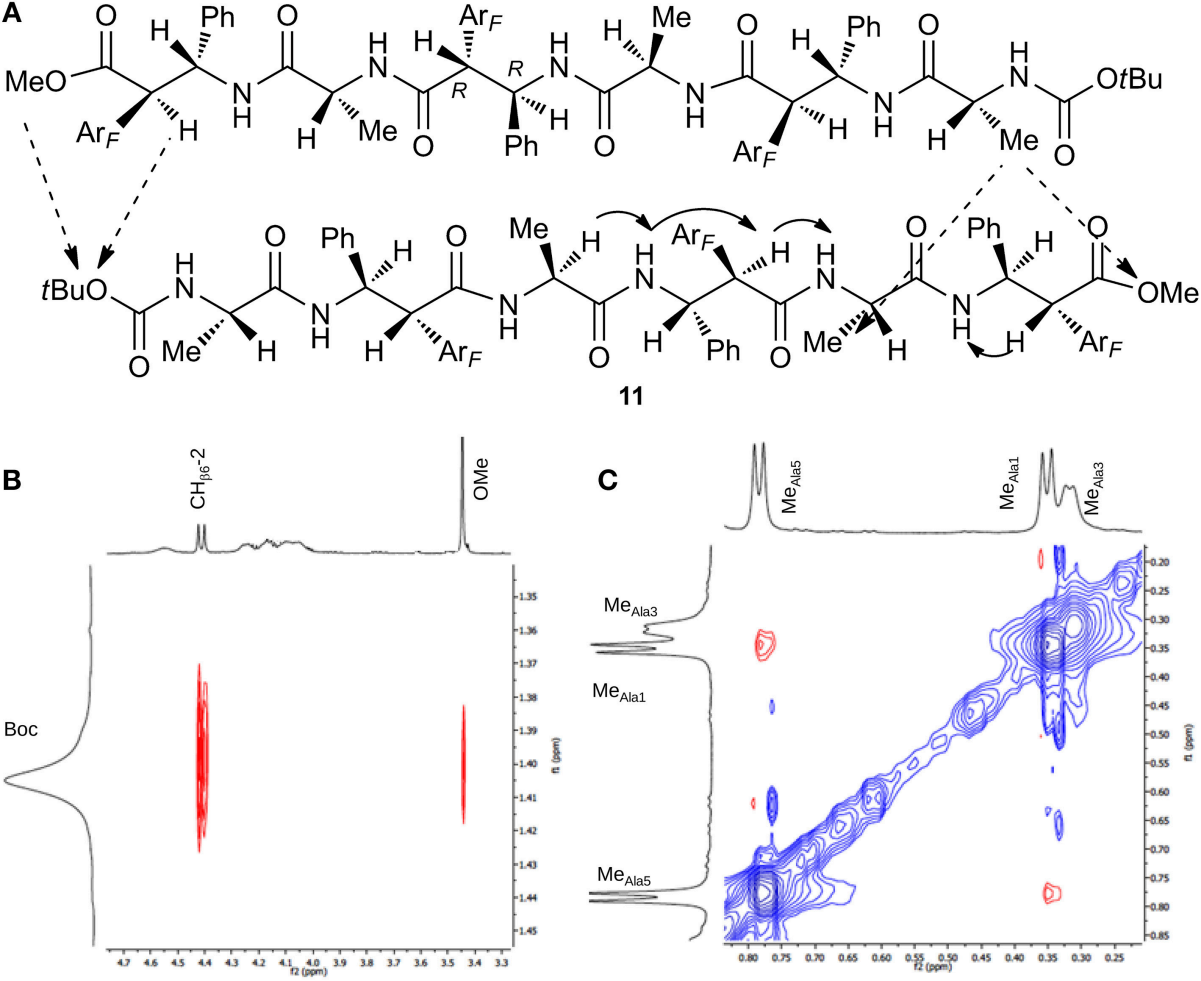
**(A)** Intra- (continuous arrow) and inter- (dotted arrow) strand NOEs for peptide **11**. **(B)** ROESY zoom of Boc region. **(C)** ROESY zoom of Me region spectrum (CDCl_3_, 10 mM, 293 K, 400 MHz, 200 ms).

Focusing on **11**, only some CH_α_/NH_β_ (i, i+1) and H_β_-2/NH_α_ (i,i+1) NOEs were detected, because several signals are overlapped preventing certain NOEs. Interestingly, the formation of an antiparallel pleated sheet arrangement in which the *C*-terminus of one strand is faced on the *N*-terminus of a second strand is supported by Noesy/Roesy experiments. Different inter-strand spatial proximities are present that are Me_Ala1_ with both Me_Ala5_(m) and OMe (vw) and Boc with both CH_β6_-2(m) and OMe(w) (Figure 6). ^1^H NMR studies at variable concentration (1.15–9.10 mM; Figure S32) showed concentration dependence chemical shift changes indicating aggregation. Noesy/Roesy experiments confirmed this tendency also at very low concentration (1.5 mM in CDCl_3_, Figure S5). DMSO-solvent titration of **11** was performed that was matched with Tocsy experiments (0%, 8% and 20% *v/v* of DMSO in CDCl_3_) to ensure the correct correlation between NH with the corresponding amino acid. A strong NH downshift was detected for NH_β6_, NH_β4_, and NH_Ala−3_ (Δδ > 1, 0.89, and 0.54, respectively) indicating their solvent exposure. A medium-weak H-bond (Δδ 0.35) was detected for NH-2. Instead, NH-1 and NH-5 are strongly involved in a H-bond (Δδ < 0.05) (Figure S7). As a result, our hypothesis is that this antiparallel sheet is stabilized by intermolecular H-bond.

The NMR analysis of **11** is also performed in DMSO-d_6_ (10 mM), that is known to be a dissociating solvent. A similar ^1^H NMR spectrum was detected concerning protons multiplicity, indicating an extended architecture, except for the NH resonances that are at lower field (Table TS4). This allowed to detect a complete set of CH_α_/NH_β_ and H_β_-2/NH_α_ (i,i+1) spatial proximities. As expected, inter-strand spatial proximities were absent in the ROESY experiment (Figure S6).

### Computational Studies

To gain additional insight on the conformational behavior of both tetra (**m7** and **m8**) and hexapeptides (**m11** and **m12**), we performed accelerated molecular dynamics (aMD) simulations in explicit CHCl_3_ solvent (Pierce et al., 2012).

Concerning the tetrapeptides, both clustering and hydrogen bond (H-bond) analyses suggested a significantly different conformational behavior for **m7** and **m8**. The analyses of the aMD trajectories of both peptides only evidenced two potential H-bonds, one between the acetyl carbonyl and NH_β2_, the other between C = O_β2_ and NH_β4_. However, the occupancy is about 20% higher in the case of **m8** (Table 2), suggesting that **m8** can adopt a more compact conformation. This behavior is also confirmed by the analysis of the ϕ, ψ, and θ dihedrals of the representative structures of the most populated clusters (Table 3).

**Table 2:** H-bonds analysis for tetrapeptideds **m7** and **m8** performed on the last 200 ns of the aMD trajectories.

**Acceptor**	**Donor**	**Fraction%**	**Avg Dist (Å)^a^**	**Avg Ang (°)^b^**
**m7**				
Ac(O)	β-2(NH)	51.6	3.0	138.4
β-2(O)	β-4(NH)	35.4	3.1	135.9
**m8**				
Ac(O)	β-2(N)	74.1	3.0	139.8
β-2(O)	β-4(N)	50.8	3.1	135.6

a *A donor-acceptor distance cutoff of 4.0 Å was requested*.

b *A donor-H···acceptor angle cutoff of 110° was requested*.

**Table 3:** Structural analysis^a^ of the top clusters for the **m7** and **m8** tetrapeptides obtained from the analysis of the last 200 ns of the aMD simulations.

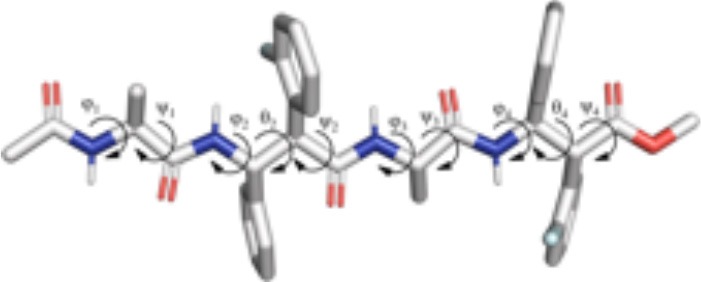											
**c#**	**pop%**	**ϕ1**	**ψ1**	**ϕ2**	**θ2**	**ψ2**	**ϕ3**	**ψ3**	**ϕ4**	**θ4**	**ψ4**
**m7**											
0	45.6	57.7 ± 128.8	−32.5 ± 118.9	−100.8 ± 131.5	175.9 ± 8.4	120.1 ± 16.0	−160.6 ± 22.2	164.5 ± 17.5	−113.0 ± 20.5	−174.6 ± 12.2	131.2 ± 23.0
1	35.0	−79.0 ± 44.4	96.8 ± 57.7	−106.3 ± 27.9	163.4 ± 13.9	77.6 ± 47.0	−58.4 ± 27.8	81.6 ± 38.7	−93.0 ± 38.7	−163.6 ± 23.4	104.6 ± 30.8
**m8**											
0	87.7	−112.3 ± 41.2	−1.5 ± 73.4	112.2 ± 23.1	−175.7 ± 9.5	−121.4 ± 28.3	−57.6 ± 52.3	102.5 ± 71.9	128.1 ± 26.0	−179.9 ± 11.3	−119.8 ± 58.7
1	9.1	−71.3 ± 34.0	150.7 ± 84.6	107.1 ± 21.5	−177.8 ± 10.1	−153.2 ± 15.5	−166.9 ± 20.0	174.6 ± 20.1	115.9 ± 38.0	170.5 ± 17.9	−98.7 ± 41.0

a *Dihedrals are measured on the non-minimized most representative conformation of each cluster. Intervals are the mean deviations of the whole cluster population from the centroid*.

**Figure 7:**
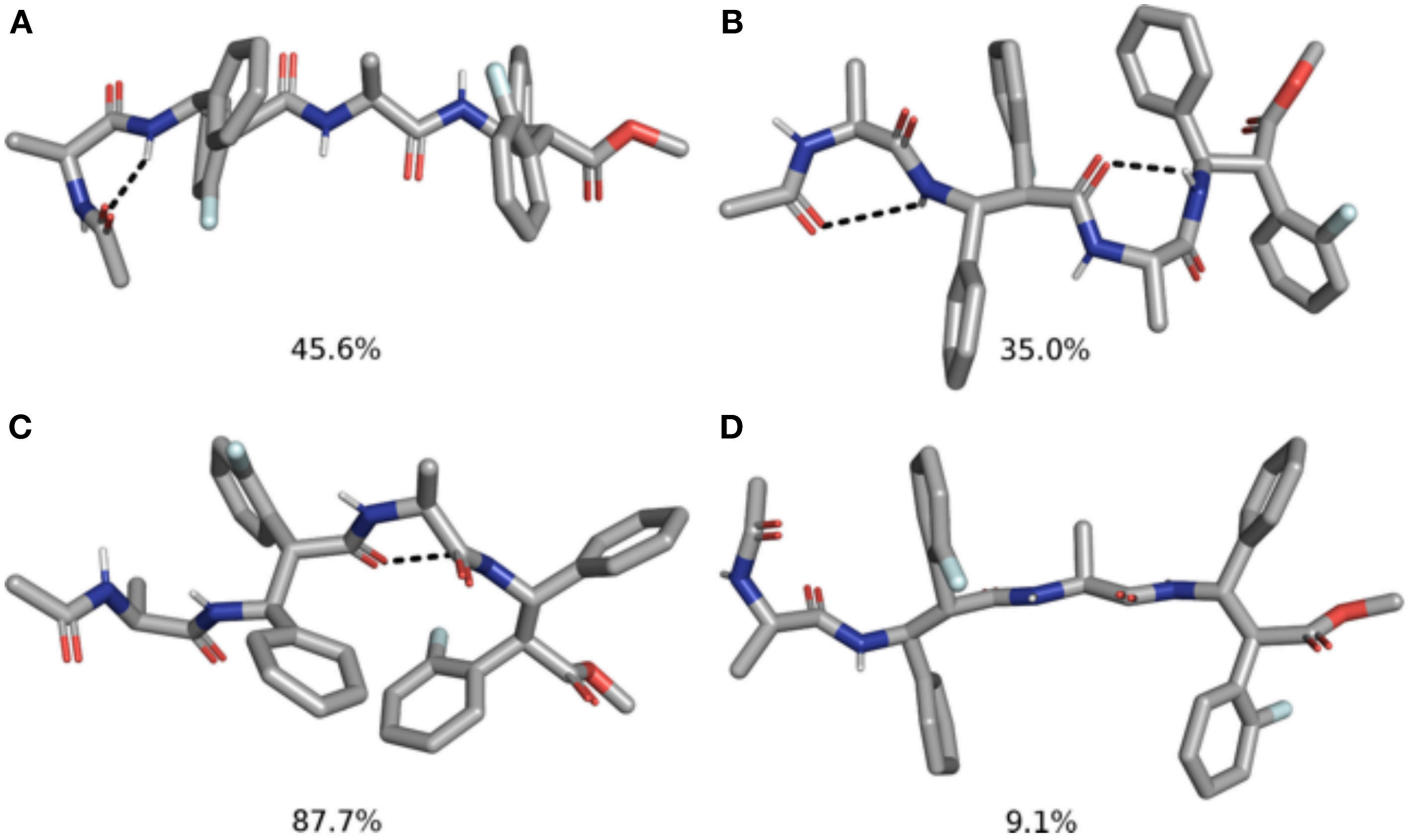
Top 2 clusters representative structures of **m7 (A,B)** and **m8 (C,D)** tetrapeptides. The populations of each cluster are indicated as a percentage. Hydrogen bonds are indicated as dashed black lines.

Indeed, **m7** prefers an almost completely extended conformation (Figure 7A), except for a γ-turn involving the acetyl cap at the *N*-terminus. This conformation is consistent with the high chemical shift observed for Me_Ala3_ (see above). At a minor extent, a geometry characterized by two γ-turns, the first involving the acetyl cap and the NH_β2_, the second between the C = O_β2_ carbonyl and NH_β4_, is also observed as the secondary cluster (Figure 7B). This conformation is possibly stabilized by a π-π interaction between the Ph_β2_ and the Ar_Fβ4_. However, the low H-bond population, the relatively long donor-acceptor distances and narrow donor-H···angles (Table 2) suggest that these γ-turns are not stable. Overall, these data suggest that **m7** can switch from a completely extended conformation to a partially folded conformation. However, this last is only marginally stabilized by π-π interactions between the two β-amino acids and by γ-turn H-bonds. The equilibrium is consequently shifted toward the extended conformation, coherently with NMR observations.

Conversely, **m8** tetrapeptide showed an opposite behavior. A highly populated primary cluster (pop = 87.7%; Figure 7C) is indeed characterized by a relatively compact geometry, showing a γ-turn between β-2 and β-4 and a π-π interaction between the Ph_β2_ and Ar_Fβ4_. This interaction is consistent with Noesy experiments (see above). A poorly sampled fully extended conformation (pop = 9.1%) was also found as a secondary cluster (Figure 7D).

**Figure 8:**
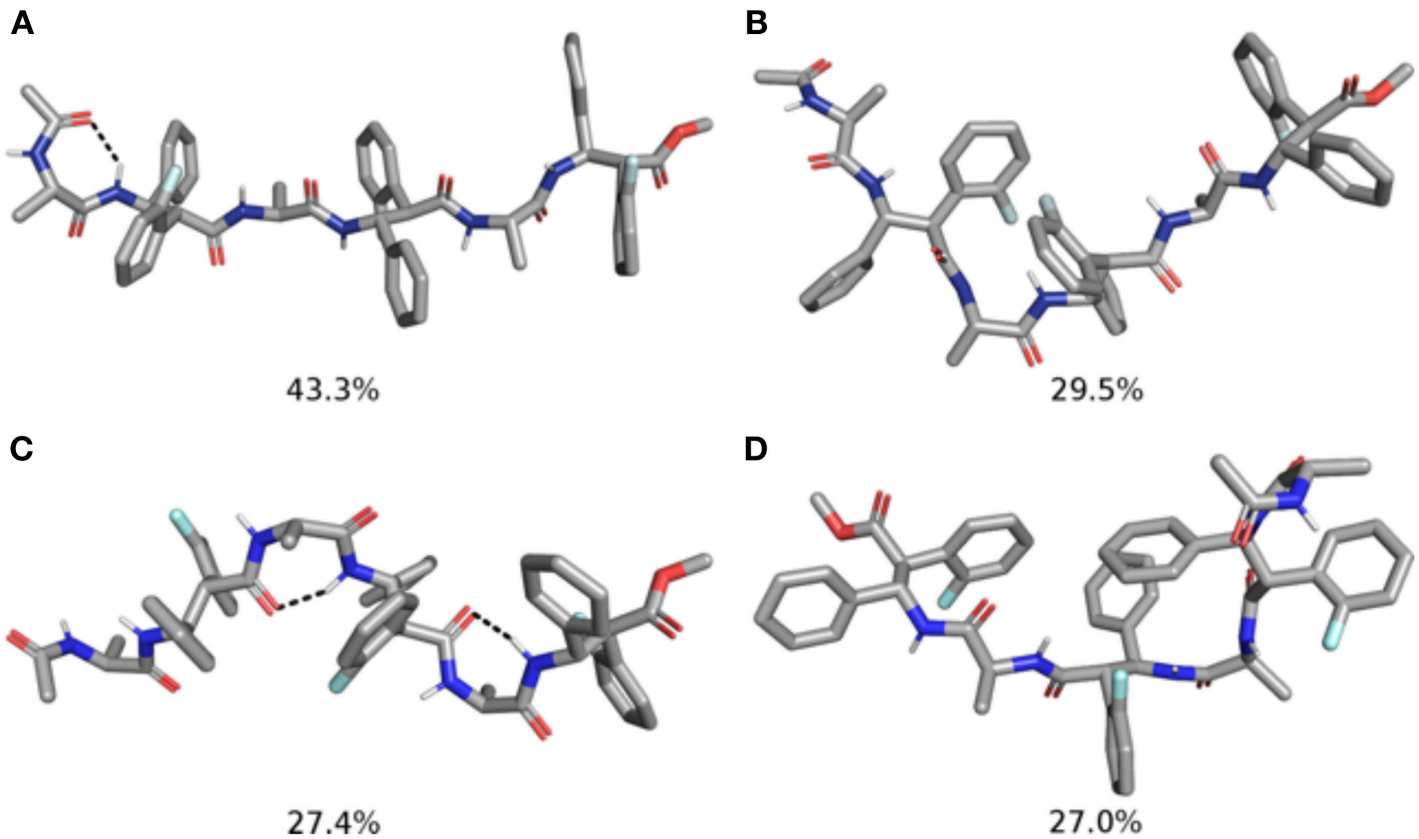
Representative structures of the two most populated clusters of **m11 (A,B)** and **m12 (C,D)** hexapeptides. The population of each cluster is indicated as a percentage. Hydrogen bonds are depicted as dashed black lines.

The analysis of the aMD simulations performed on **m11** and **m12** hexapeptides led to similar conclusions, compared to the tetrapeptide series. Indeed, **m11** most populated cluster (pop = 43.3%) corresponds to an extended conformation, as showed by the ϕ, ψ, and θ dihedrals of the corresponding representative structure (Table 4 and Figure 8A). The second most populated cluster (pop = 29.5%) represents conformations showing π-π interactions involving the Ar_F_ of both β-2 and β-4, while the *C*-terminus remains extended (Table 4 and Figure 8B). Only the third cluster (pop = 19.8%) presented the Ar_F_/Ph π-π interactions between the aryl groups of β-2/β-4 and of β-4/β-6, similarly to what observed in **m7**. We could also observe γ-turns between the backbone C = O of Ac capping group with NH_β2_, and carbonyls of β-2 and β-4 with NH_β4_ and NH_β6_ (Table 5).

**Table 4:** Structural analysis of the top 3 clusters for the **m11** and **m12** hexapeptides obtained from the analysis of the last 200 ns of the aMD simulations.

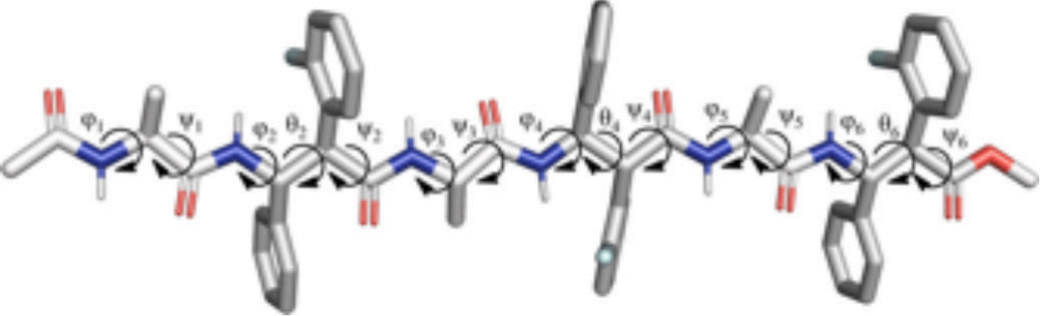																
**c#**	**pop%**	**ϕ1**	**ψ1**	**ϕ2**	**θ2**	**ψ2**	**ϕ3**	**ψ3**	**ϕ4**	**θ4**	**ψ4**	**ϕ5**	**ψ5**	**ϕ6**	**θ6**	**ψ6**
**m11**																
0	43.3	−59.2 ± 51.6	75.0 ± 59.5	−110.1 ± 25.8	179.8 ± 9.2	133.5 ± 18.4	−150.9 ± 20.1	140.6 ± 20.2	−117.7 ± 20.5	173.8 ± 8.5	122.4 ± 19.9	−104.5 ± 35.1	115.0 ± 47.6	−101.6 ± 27.9	155.6 ± 23.8	110.5 ± 26.2
1	29.5	−155.7 ± 58.5	164.9 ± 64.6	−147.4 ± 27.5	170.9 ± 9.3	126.0 ± 23.6	−123.6 ± 48.5	51.1 ± 40.7	−165.6 ± 31.2	172.5 ± 10.8	152.9 ± 32.3	−151.2 ± 23.1	128.7 ± 34.7	−89.0 ± 35.1	−161.2 ± 24.2	152.2 ± 30.2
2	19.8	−78.0 ± 35.2	77.3 ± 52.9	−162.7 ± 36.8	−179.1 ± 10.0	148.2 ± 29.2	−62.0 ± 24.8	57.5 ± 32.4	−106.5 ± 31.1	175.2 ± 9.0	90.6 ± 38.6	−89.3 ± 15.8	36.1 ± 37.7	−124.8 ± 24.1	−169.5 ± 19.3	143.5 ± 26.2
**m12**																
0	27.4	−74.1 ± 19.9	−20.8 ± 76.2	106.5 ± 24.8	−175.6 ± 9.3	−140.6 ± 25.5	65.3 ± 102.2	−34.7 ± 66.5	117.2 ± 21.5	−171.8 ± 10.2	−161.9 ± 40.3	80.3 ± 99.5	−45.2 ± 81.5	102.0 ± 32.9	173.1 ± 14.7	−150.1 ± 26.6
1	27.0	−72.5 ± 21.8	0.5 ± 71.3	101.6 ± 29.2	177.5 ± 11.8	−141.3 ± 21.6	−110. 1 ± 54.6	−8. 8 ± 50.3	174.0 ± 54.5	−165.8 ± 14.8	−148.2 ± 26.3	−150.5 ± 35.5	157.1 ± 60.6	88.3 ± 46.4	175.5 ± 14.3	−106.0 ± 29.5
2	16.4	−176.2 ± 85.5	114.2 ± 61.7	103.3 ± 26.1	178.9 ± 12.4	−144.4 ± 22.7	−129.3 ± 27.6	−168.7 ± 37.1	89.2 ± 53.6	−175.7 ± 9.2	−106.0 ± 37.5	−136.0 ± 36.8	147.4 ± 51.4	179.9 ± 44.0	175.1 ± 16.8	−134.3 ± 21.5

**Table 5:** H-bonds Analysis for Hexapeptides **m11** and **m12** Performed on the Last 200 ns of the aMD Trajectory.

**Acceptor**	**Donor**	**Fraction%**	**Avg Dist (Å)^a^**	**Avg Ang (°)^b^**
**m11**				
Ac(O)	β-2(N)	43.8	3.0	137.7
β-2(O)	β-4(N)	39.0	3.1	136.4
β-4(O)	β-6(N)	31.7	3.1	136.2
**m12**				
Ac(O)	β-2(N)	60.4	3.1	139.6
β-2(O)	β-4(N)	47.5	3.0	137.2
β-4(O)	β-6(N)	46.9	3.0	137.0

a *A donor-acceptor distance cutoff of 4.0 Å was requested*.

b *A donor-H···acceptor angle cutoff of 110° was requested*.

The top two clusters of the **m12** hexapeptide are relatively low populated (pop = 27.4 and 27.0%, respectively). However, they both show a folded conformation, with π-π interactions between the aromatic rings of the β-amino acids, and the above described γ-turns (Figures 8C,D; Table 4). This behavior resembles what previously observed for the tetrapeptides series, where **m8** principal geometry was more compact, compared to **m7**. A similar behavior between the hexa- and tetrapeptide series was also observed in terms of H-bonds. Indeed, the occupancies of the H-bonds defining the γ-turns are about 15% higher in **m12**, compared to **m11** (Table 5). In the hexapeptide series, also a relatively populated third cluster (pop = 16.4%) showed a partially folded representative structure. Indeed, computed ϕ, ψ, and θ dihedrals (ψ3, ϕ4, and θ4, in particular), assume values that are compatible with interactions between the Ph_β2_ and the Ar_Fβ4_, as well as between the Ph_β4_ and Ar_Fβ6_.

**Figure 9:**
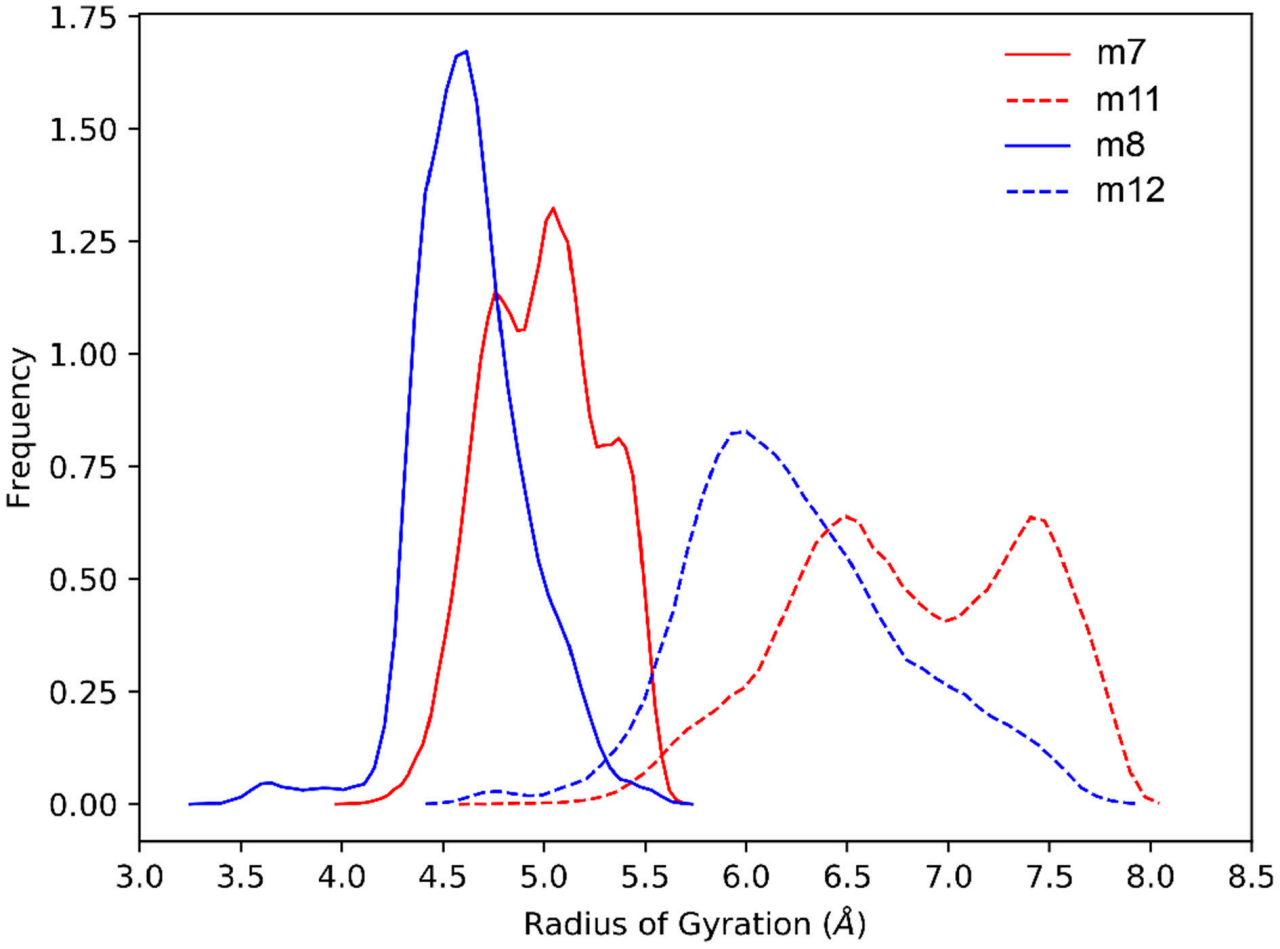
Comparison of the normalized sampling frequencies of RoG calculated from the last 200 ns of the aMD trajectories of **m7**, **m8**, **m11**, and **m12**.

Overall, the computational analysis shows a coherent influence of the stereochemical configuration of α and β carbons on the investigated β-amino acids inserted in model Ac(*S-*Ala*-*β-Fpg)_n_OMe peptides. Indeed, our observation suggest that β*-*2*R*,3*R*-Fpg helps to stabilize an extended conformation. Conversely, β*-*2*S*,3*S*-Fpg seems to induce more compact conformations, possibly stabilized by inter-residue π-π interactions and/or by γ-turns. This behavior is also supported by *J*_*NH*−*CHβ*_ and *J*_2,3_ coupling constants obtained by ^1^H NMR experiments. Indeed, larger *J* values were obtained for both **7** and **11**, compared to **8** and **12**, suggesting a more folded conformation for these latter peptides (Li et al., 2015). To provide additional evidences of this behavior, we compared the frequency of the radius of gyrations (RoG) sampled in the last 200 ns of simulation (Figure 9). For both tetra- and hexapeptides, it can be observed that a larger RoG is sampled more frequently for peptides containing β*-*2*R*,3*R*-Fpg, compared to peptides containing β*-*2*S*,3*S*-Fpg. Figure 9 also shows that two peaks are found for **m11**, centered at 6.5 and 8.0 Å. This is coherent with the results of the clustering analysis, which evidence the possibility of a “bending” of the extended geometry at the Ala3/β4 level (Figure 8). **m7** instead shows a “spiked” peak centered at 5.0 Å, corresponding to the representative geometry shown in Figure 7A. The two “spikes” at 4.8 and 5.4 Å correspond to the breaking of the *N*-terminal γ-turn (involving the acetyl cap) and to the forming of the additional γ-turn (involving β-2 and β-4), respectively. Conversely, both **m8** and **m12** show a main peak (sharper, for **m8**) that is centered onto a RoG value that is significantly lower to that computed for the corresponding peptides containing β*-*2*R*,3*R*-Fpg.

## Conclusions

Taken together these data, we can conclude that the architecture of the above peptides depends on the balance between the features of the selected amino acids, i.e., the *syn* β-amino acid, favoring an extended conformation, and alanine, inducing turns. The chirality of the two stereogenic centers of β-amino acid remarkably affects the peptide 3D-structures. A preferred extended conformation was found matching the β-*R,R*-amino acid with *S*-alanine. This is confirmed by *J* values of both tetra- and hexapeptides containing β*-*2*R*,3*R*-Fpg as well as by computational analysis. The extended conformation of this peptide induces the formation of an antiparallel β-sheet, also at low concentration, as documented by NMR data of **11**. Our hypothesis is that the formation of this pleated sheet is favored by π,π-interactions of aromatic moieties and is stabilized by H-bonds. IR analysis of this peptide indicates the involvement of NHs in H-bonds, confirmed by the titration experiment (NH-1 and NH-6 involvement). The NH-network proposed by computational experiments does not fit with NMR data. On the other hand, computational data are consistent with a **11** single strand and not with pleated sheet architecture. In this case the formation of H-bonds is driven by γ-turn formation.

β-*S,S*-amino acid with *S*-alanine gives more compact folded structures that are probably driven by alanine moieties. In fact, peptides containing β*-*2*S*,3*S*-Fpg display a higher stability and a larger content of an ordered secondary structure as compared to the peptides containing β*-*2*R*,3*R*-Fpg. Both NMR and computational data support the formation of a turn for **8** stabilized by π-π interactions between Ph_β2_ and Ar_Fβ4_. IR absorption analysis and experiments at variable temperature confirm that the longer oligomer **12** is stabilized by intramolecular C = O···H-N bonds. On the other hand, *J* values indicate that an equilibrium occurred between multiple compact conformations.

## Author Contributions

MG conceived the research. RB and SP carried out change with RB, EB, and SP carried out the synthesis. MG, FC, and FF carried out the spectroscopic analyses. AC and IM carried out the computational studies. MG and AC supervised the work. MG, AC, and RB wrote the paper. All the authors revised the manuscript.

### Conflict of Interest Statement

The authors declare that the research was conducted in the absence of any commercial or financial relationships that could be construed as a potential conflict of interest.
