# Supplementary material for: Fluoro-Aryl Substituted α,β2,3-Peptides in the Development of Foldameric Antiparallel β-Sheets: A Conformational Study
Source: Front Chem. 2019 Apr 2;7:192. doi: 10.3389/fchem.2019.00192 (PMC6454073; doi:10.3389/fchem.2019.00192)
Supplement: Supplementary file 1 [file Data_Sheet_1.PDF]

Fluoro Aryl Substituted α,$\beta^{2,3}$-Peptides in the Development of Foldameric Antiparallel β–Sheets: a Conformational Study.

Raffaella Bucci,[†] Alessandro Contini,[†] Francesca Clerici,[†] Egle Maria Beccalli,[†] Fernando Formaggio,[§] Irene Maffuicci,[‡] Sara Pellegrino[†] and Maria Luisa Gelmi*[†]

[†]*Department of Pharmaceutical Sciences (DISFARM), University of Milan, via Venezian 21, I-20133 Milano, Italy*

[§]*Department of Chemistry, University of Padova, via Marzuolo 1, I-35131 Padova, Italy*

[‡]*CNRS UMR 7025, Génie Enzymatique et Cellulaire. Centre de Recherche de Royallieu. CS 60319, 60203 Compiègne Cedex, France and Sorbonne Universités, Université de Technologie de Compiègne, Génie Enzymatique et Cellulaire. Centre de Recherche de Royallieu. CS 60319, 60203 Compiègne Cedex, France*

SUPPLEMENTARY MATERIAL

Figure S1. Backbone heavy atoms RMSD distributions from the extended conformation for peptides containing 2*R*,3*R*- and 2*S*,3*S*-β-Fpg	S2
Experimental procedures and characterization data for peptides **3-5** and **7-12**	S2-S15
Figure S9. FTIR absorption spectra (N-H stretching region) for peptides **1,7,11** and **2,10,12**	S15
NMR spectra for peptides **3-5** and **7-12**	S16-S31

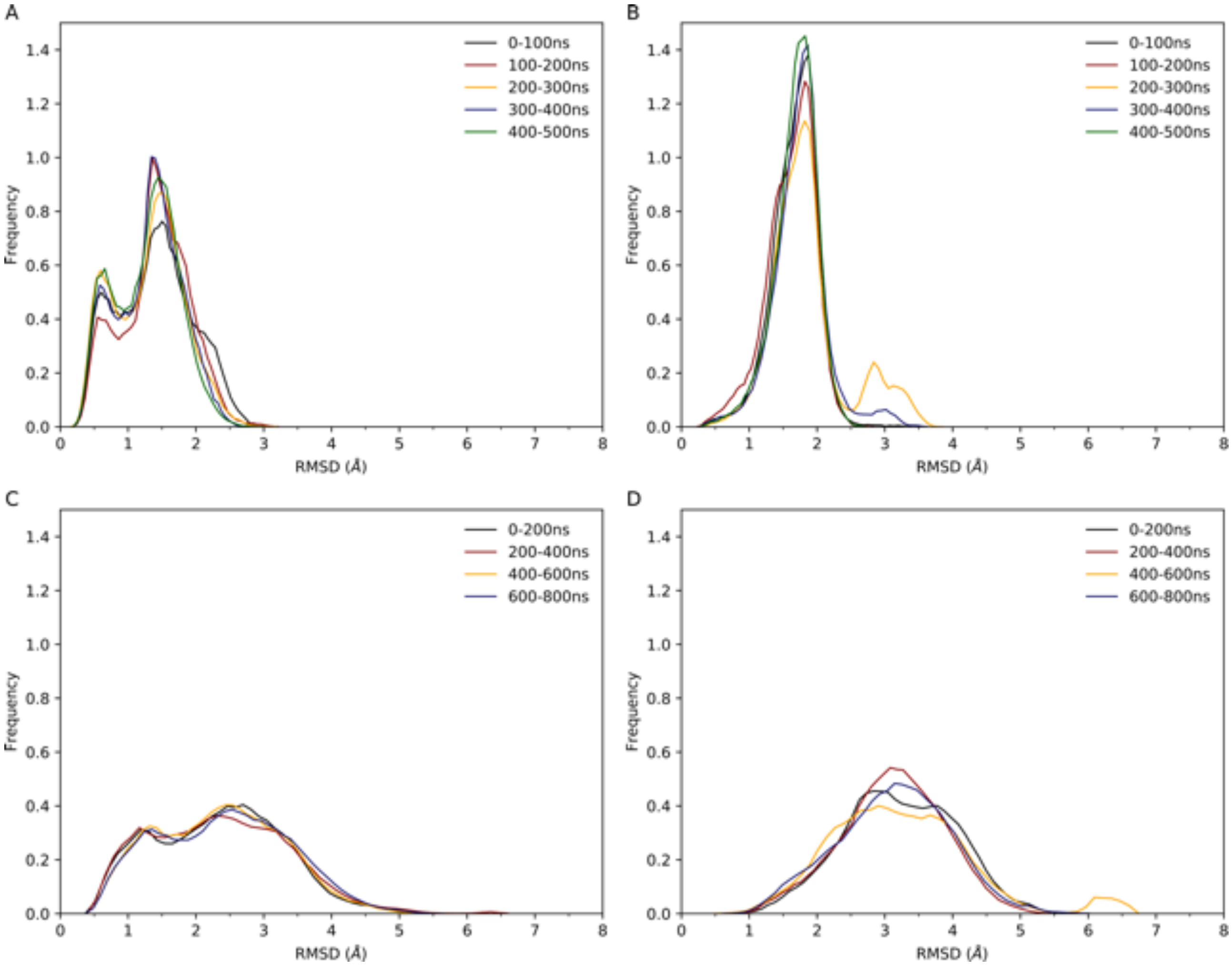

Figure S1. Backbone heavy atoms RMSD distributions from the extended conformation for the tetrapeptides **7** (A) and **8** (B) and the hexapeptides **11** (C) and **12** (D) calculated on a 100 ns and 200 ns time intervals, respectively.

*General Procedure for the Preparation of dipeptides **3** and **4**.* Dipeptide **1** or **2** (300 mg, 0.7 mmol)[1] was suspended in an aqueous solution of HCl 1M (15 mL). After heating heated for 24 h at 80 °C, the solvent was removed under reduced pressure, affording the unprotected dipeptide as HCl salt. The crude compound was used without further purification. It was dissolved in CH_2Cl_2 (15 mL, 0.05 M) and cooled to 0 °C. Triethylamine (0.3 mL, 2.1 mmol) and Boc_2O (213 mg, 0.9 mmol) were added and the solution was left to react overnight at 25 °C. A saturated solution of $KHSO_4$ (15 mL) was then added. The aqueous layer was separated and the organic one was first washed with a saturated solution of NaCl (15 mL) and then dried over Na_2SO_4. The solvent was removed under reduced pressure, affording the dipeptide **3** from **1** and 4 from **2** as colorless oil.

---

[1] Bonetti A., Pellegrino S., Das P., Yuran S., Bucci R., Ferri N., Meneghetti F. Castellano C., Reches M., Gelmi M. L., *Org. Lett.* **2015** 17 (18), 4468-4471

H-*S*-Ala-β-2*R*,3*R*-Fpg-OH HCl: 99 %. M.p. 153 °C; IR (KBr) ν_{max} 3430, 1713, 1673 cm^{-1}; ^1H NMR (300 MHz, CD_3OD) δ 7.67-7.15 (m, 9H), 5.77 (d, *J* 11.8, 1H), 4.53 (d, *J* 11.8, 1H), 3.63 (m, 1H), 0.88 (d, *J* 7.15, 3H); ^{13}C NMR (75 MHz, $CDCl_3$) *δ* 16.2, 48.8, 48.7, 54.3, 115.2 (d, *J* 22.9), 123.5 (d, *J* 14.6), 124.4 (d, *J* = 3.5), 127.6 (x2), 127.9, 128.5 (x2), 129.5 (d, *J* 8.06), 129.6, 140.1, 161.1 (d, *J* 245.6), 168.6, 172.4; MS (ESI): *m/z* calcd for [$C_{18}H_{19}FN_2O_3$]: 330.14; found: *m/z* 331.16 $[M+H]^+$.

H-*S*-Ala-β-2*S*,3*S*-Fpg-OH HCl: 99 %. M.p. 153 °C. IR (KBr) ν_{max} 3220, 1719, 1689 cm^{-1}; ^1H NMR (300 MHz, CD_3OD) *δ* 7.67-7.01 (m, 9H), 5.69 (d, *J* 11.7, 1H), 4.64 (d, *J* 11.8, 1H), 3.53 (m, 1H), 1.21 (d, *J* 7.0, 3H). ^{13}C NMR (75 MHz, $CDCl_3$) *δ* 16.3, 48.8, 48.7, 54.9, 115.2 (d, *J* 22.8), 123.6 (d, *J* 14.5), 124.5 (d, *J* 3.5), 127.7 (x2), 128.0, 128.6 (x2), 129.5 (d, *J* 8.06), 129.7, 140.5, 161.4 (d, *J* 246.1), 168.8, 172.6; MS (ESI): *m/z* calcd for [$C_{18}H_{19}FN_2O_3$]: 330.14; found: *m/z* 331.4 $[M+H]^+$.

Boc-*S*-Ala-β-2*R*,3*R*-Fpg-OH (3): 96%. IR (KBr) ν_{max} 3430, 1717, 1658 cm^{-1}; ^1H NMR (200 MHz, $CDCl_3$) *δ* 7.58-7.00 (m, 9H), 6.84 (brs, 1H), 5.69 (t, *J* 9.9, 1H), 4.98 (brs, 1H), 4.45 (d, *J* 9.9, 1H), 3.98-3.82 (m, 1H), 1.33 (s, 9H), 0,9 (d, *J* = 7.1 Hz, 3H); ^{13}C NMR (50 MHz, $CDCl_3$) *δ* 17.9, 28.4(x3), 48.8, 49.8, 54.5, 80.5, 115.5 (d, *J* 22.9 Hz), 122.4 (d, *J* 14.0), 124.7 (d, *J* 3.1), 127.6 (x2), 128.1, 128.8 (x2), 129.9 (d, *J* 8.3), 130.2 (brs), 139.6, 155.8, 161.1 (d, *J* 246.1), 171.9, 173.7; MS (ESI): *m/z* calcd for [$C_{23}H_{27}FN_2O_5$]: 430.19; found: *m/z* 453.3 $[M+Na]^+$.

Boc-*S*-Ala-β-2*S*,3*S*-Fpg-OH (4): 96%. IR (KBr) ν_{max} 2925, 1716 cm^{-1}; ^1H NMR (300 MHz, $CDCl_3$) *δ* 7.60-6.90 (m, 9 H), 7.00-6.40 (brs, 2H), 5.70 (t, *J* 9.1, 1H), 4.95 (brs, 1H), 4.50 (d, *J* 9.6, 1H), 4.00-3.70 (m, 1H), 1.2 (brs, 9H), 0.95 (brs, 3H); ^{13}C NMR (75 MHz, $CDCl_3$) *δ* 17.9, 29.9 (x3), 49.1, 52.4, 54.3, 68.4, 115.4 (d, *J* 22.9 Hz), 122.7 (d, *J* 14.1 Hz), 124.6 (d, *J* 2.9 Hz), 127.5(x2), 128.1, 128.8 (x2), 129.7 (d, *J* 8.3), 130.4 (d, *J* 3.1), 139.8, 156.1, 161.1 (d, *J* 246.1 Hz), 172.0, 173.9; MS (ESI): *m/z* calcd for [$C_{23}H_{27}FN_2O_5$]: 430.19; found: *m/z* 453.1 $[M+Na]^+$.

General Procedure for Boc-deprotection. Dipeptide **1** or tetrapeptide **7** or **8** (2.3 mmol) was dissolved in CH_2Cl_2 (20 mL) and cooled to 0 °C. After 10 min., TFA (20 mL) was slowly dropped to the solution. The mixture was stirred at 25 °C for 2h. The solvent was removed under reduced pressure. The salt, was dissolved in CH_2Cl_2 (10 mL) and washed first with a saturated solution of $NaHCO_3$ (4 mL) and then with a saturated solution of NaCl (6 mL). The organic layer was dried over Na_2SO_4. The solvent was removed under reduced pressure and the crude was crystallized from Et_2O affording pure free dipeptide **5** from **1**, and tetrapeptides **9** or **10**, from **7** and **8** respectively.

H-*S*-Ala-β-2*R*,3*R*-Fpg-OMe (5): 98%. Mp 87 °C (AcOEt/*n*-hexane); $[\alpha]_D$ +32.1 (*c* 6, $CHCl_3$); IR (KBr) ν_{max} 1733, 1661 cm^{-1}; ^1H NMR ($CDCl_3$, 200 MHz) *δ* 7.74 (d, *J* 9.6, 1H), 7.63-7.00 (m, 9H), 5.69 (t, *J* 10.1, 1H), 4.54 (d, *J* 10.4, 1H), 3.53 (s, 3H), 3.29-3.22 (m, 1H), 1.99 (brs, 2H), 1.04 (d, *J*

7.0, 3H);^{13}C NMR ($CDCl_3$, 50 MHz) δ 21.1, 49.2, 50.7, 52.5, 54.7, 115.3 (d, *J* 22.7), 123.1 (d, *J* 14.1), 124.9 (d, *J* 3.5), 127.7 (x2), 128.3, 129.4 (x2), 129.9 (d, *J* 8.3), 130.2 (d, *J* 3.1), 140.4, 161.2 (d, *J* 245.5 Hz), 171.4, 174.0; MS (ESI): *m/z* calcd for [$C_{19}H_{21}FN_2O_3$]: 344.15; found: *m/z* 345.3 $[M+H]^+$. Elemental analysis calcd (%) for $C_{19}H_{21}FN_2O_3$: C, 66.26; H, 6.15; N, 8.13; found: C, 65.89; H, 6.37; N, 7.95.

H-[*S*-Ala-β-2*R*,3*R*-Fpg]$_2$OMe (9): 96%. Mp 90 °C (AcOEt/*n*-hexane); $[\alpha]_D$ +33.6 (*c* 4, $CHCl_3$); IR (KBr) ν_{max} 3278, 1739, 1686, 1666 cm^{-1}; ^{1}H NMR ($CDCl_3$, 200 MHz) *δ* 7.74 (d, *J* 8.9), 7.70-7.57 (m, 1H), 7.55-7.45 (m, 1H), 7.40-6.90 (m, 16H), 6.48 (d, *J* 9.3, 1H), 6.10 (d, *J* 7.0, 1H), 5.63 (t, *J* 10.2), 5.59 (t, *J* 10.0), 4.39 (d, *J* 10.5, 1H), 4.12 (d, *J* 10.3, 1H), 3.97-3.92 (m, 1H), 3.48 (s, 3H), 3.22-3.20 (m, 1H), 1.84 (brs, 2H), 1.03 (d, *J* 6.5, 3H), 0.55 (d, *J* 6.8, 3H); ^{13}C NMR ($CDCl_3$, 50 MHz) δ 17.9, 20.3, 48.8, 49.2, 50.8 (x2), 52.6, 55.0 (x2), 115.2 (d, *J* 22.9), 115.7 (d, *J* 22.8 Hz), 122.7 (d, *J* 14.0 Hz), 123.4 (d, *J* 13.5), 124.9 (d, *J* 3.4), 125.0, (d, *J* 4.4 Hz), 127.5 (x2), 127.6 (x2), 128.1, 128.3, 128.9 (x2), 129.0(x2), 129.6 (d, *J* 8.5 Hz), 129.9 (d, *J* 8.1 Hz), 130.0 (d, *J* 2.9 Hz), 130.2 (d, *J* 2.8 Hz), 139.7, 140.6, 161.0 (d, *J* 241.3 Hz), 162.1 (d, *J* 241.1 Hz), 169.7, 170.8, 171.2, 174.4; MS (ESI): *m/z* calcd for [$C_{37}H_{38}F_2N_4O_5$]: 656.28; found: *m/z* 681.2 $[M+Na]^+$

H-[*S*-Ala-β-2*S*,3*S*-Fpg]$_2$OMe (10): 97%. Mp: 91 °C (AcOEt/*n*-hexane); $[\alpha]_D$ = +51.2 (*c* 4, $CHCl_3$); IR (KBr) ν_{max} 1742, 1660 cm^{-1}; ^{1}H NMR ($CDCl_3$, 200 MHz) *δ* 8.02 (d, *J* 10.1, 1H), 7.60-6.90 (m, 18H), 6.61 (d, *J* 9.3, 1H), 6.00 (d, *J* 5.1, 1H), 5.72 (t, *J* 9.5 Hz, 1H), 5.55 (t, *J* 9.7 Hz, 1H), 4.44 (d, *J* 10.2 Hz, 1H), 4.23 (d, *J* 9.2 Hz, 1H), 4.10-4.01 (m, 1H), 3.48 (s, 3H), 3.41-3.34 (m, 1H), 1.28 (brs, 2H), 0.9 (d, *J* 7.0 Hz, 3H), 0.93 (d, *J* 7.0 Hz, 3H); ^{13}C NMR ($CDCl_3$, 50 MHz) δ 17.9, 21.2, 49.2, 49.6, 50.2, 50.9, 52.6, 53.9, 55.2, 115.3 (d, *J* 15.9), 115.6 (d, *J* 15.7), 123.0 (d, *J* 14.1), 123.5 (d, *J* 13.5), 124.9 (x2), 127.7 (x4), 128.1, 128.3, 128.9 (x4), 129.7 (d, *J* 8.5), 130.1 (d, *J* 8.2), 130.3, 130.5, 139.6, 140.5, 159.5 (d, *J* 3.1), 162.6 (d, *J* 4.5), 170.0, 171.1, 171.4, 174.3; MS (ESI): *m/z* calcd for [$C_{37}H_{38}F_2N_4O_5$]: 656.28; found: *m/z* 657.3 $[M+H]^+$.

General Procedure for the coupling reaction. Dipeptide **3** or **4** (77.4 mg, 0.18 mmol) was dissolved in CH_2Cl_2 (2 mL). The solution was cooled to 0°C and then EDC (34.9 mg, 0.18 mmol) and EtCN-oxime (25.9 mg, 0.18 mmol) were added. The mixture was stirred at 0°C for 1h. Dipeptide **5** or **6** or tetrapeptide **9** or **10** (0.17 mmol), dissolved in CH_2Cl_2 (2 mL) and DIPEA (8.64 μL, 0.05 mmol) were added and the mixture was stirred at 25 °C for 24 h. A saturated solution of $NaHCO_3$ (4 mL) was added. The aqueous layer was separated and the organic layer was washed with a saturated solution of NH_4Cl (4 mL), and then with a saturated solution of NaCl (4 mL). After drying over Na_2SO_4, the solvent was removed under reduced pressure and the crude residue was purified by column

chromatography on silica gel (*n*-hexane/AcOEt, 2:3), affording tetrapeptide **7** from **5** or **8** from **6** or hexapeptide **11** from **9** or **12** from **10**.

Boc-[*S*-Ala-β-2*R*,3*R*-Fpg]$_2$-OMe (7): 74%. Mp: 99°C; $[\alpha]_D$ = +37.4 (*c* 3.1 in $CHCl_3$). NMR data are reported in Table TS1; MS (ESI): *m/z* calcd for [$C_{42}H_{46}F_2N_4O_7$]: 756.33. Found *m/z* (%): 779.4 $[M+Na]^+$.

Boc-[*S*-Ala-β-2*S*,3*S*-Fpg]$_2$-OMe (8): 76%. Mp: 88 °C; $[\alpha]_D$ = +56.1 (*c* 3.4 in $CHCl_3$). NMR data are reported in Table TS2; MS (ESI): *m/z* calcd for [$C_{42}H_{46}F_2N_4O_7$]: 756.33. Found *m/z* (%): 779.0 $[M+Na]^+$.

Boc-[*S*-Ala-β-2*R*,3*R*-Fpg]$_3$-OMe (11): 55%. Mp: 102°C; $[\alpha]_D$ = +39.1 (*c* 1.9 in $CHCl_3$); NMR data are reported in Tables TS3 and TS4; MS (ESI): *m/z* calcd for [$C_{60}H_{63}F_3N_6O_9$]: 1068.46. Found *m/z* (%): 1091.7 $[M+Na]^+$.

Boc-[*S*-Ala-β-2*S*,3*S*-Fpg]$_3$-OMe (12): 62%. Mp: 108°C; $[\alpha]_D$ = +58.2 (*c* 2.8 in $CHCl_3$); NMR data are reported in Table TS5; MS (ESI): *m/z* calcd for [$C_{60}H_{63}F_3N_6O_9$]: 1068.46. Found *m/z* (%): 1092.2 $[M+Na]^+$.

Table TS1. Tetrapeptide **7** ($CDCl_3$, 31 mM, 293 K, 500 MHz)

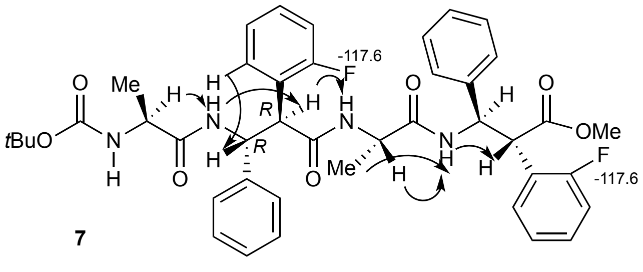

AA	atom		^{1}H	*J* (Hz)	^{13}C	H (Noesy)[a] F$_\delta$ (Hoesy)
**Ala-1**	CO				171.6	
	CH		3.92	brs	49.4 br	NH$_{β2}$(m), Me$_{Ala1}$(vs), NH$_{Ala1}$(vvw)
	Me		0.86	*J* 6.9	18.4	NH$_{Ala1}$(m), CH$_{Ala1}$(vs)
	NH		5.08	brs		Me$_{Ala1}$(m), Boc (vw) CH$_{Ala1}$(vvw)
	Boc	Me	1.39		28.4	NH$_{Ala1}$(vw)
		C			79.8	
		CO			155.5	
***Beta*-2**	CO				169.2	
	2		4.19	d, *J* 11.0	50.3	NH$_{β2}$(m), Ar(7.35s, 7.61 vvw), NH$_{Ala3}$(s)
	3		5.76	brs	54.4	Ar(7.01vw, 7.35vs, 7.61vs);
	NH		6.99	Overl.		CH$_{Ala1}$(m), H$_{β2}$-2(m), Ar(7.32s)
	Ar$_F$		7.61$_{F-6}$<br>7.23$_{F-4}$<br>7.04$_{F-5}$<br>6.98$_{F-3}$		129.$_{F-6}$<br>128.5$_{F-4}$<br>124.5 $_{F-5}$ (*J* 9.8)<br>115.3$_{F-3}$ (*J* 17.0)	Ar(7.04vs, 7.23m), H$_{β2}$-3(vs)

	 <br>Ph	-<br><br>7.33-7.16		162.2$_{F2}$ (J 242.2)<br>123.0$_{F1}$ (J 13.6)<br>127.3[b], 129.5,<br>127.8,[b] 140.0 (q)	 <br>7.35: $H_{\beta2}$-3(s), $H_{\beta2}$-2(vs), Ar$_{F-5}$(7.03m)<br>Fδ[c]
**Ala-3**	CO			170.5	
	CH	3.98	brs	48.4	Me_{Ala3}(s), $NH_{\beta4}$(s), NH_{Ala3}(w)
	Me	0.48	d, J 6.9	17.6	H_{Ala3}(s), $NH_{\beta4}$-3(w), NH_{Ala3}(m),
	NH	6.39	brs		$H_{\beta2}$-2(vs), Me_{Ala3}(m), CH_{Ala3}(w);
***Beta*-4**	CO			170.9	
	2	4.38	d, J10.7	48.7 (J_{CF} 1.9)	Ar(7.18 m; 7.49w)
	3	5.62	t, J10.3	55.3	$NH_{\beta4}$(m), Ar(7.16s, 7.49s)
	NH	6.52	d, J10.7		Me_{Ala3}(w), H_{Ala3}(s), $H_{\beta4}$-2(m), $H_{\beta4}$-3(w), Ar(7.16 m)
	Ar$_F$<br><br><br><br><br><br>Ph	7.49$_{F-6}$<br>7.23$_{F-4}$<br>7.06$_{F-5}$<br>6.94$_{F-3}$<br>-<br>-<br>7.30-7.16		129.6$_{F-6}$<br>128.5$_{F-4}$<br>124.5$_{F-5}$ (J 9.8)<br>115.0$_{F-3}$ (J 16.5)<br>162.2$_{F2}$ (J 242.2)<br>122.4$_{F1}$ (J14.4)<br>129.5[b], 128.6[b],<br>127.3, 139.2(q)	F_6: Ar(7.06s), $H_{\beta4}$-3(s), $H_{\beta4}$-2(vw),<br>F_5: Ar(7.49s)<br><br><br><br>Fδ[c]
	OMe	3.52	s	52.1	

[a]400 ms. [b]Tentatively assigned. [c]δ_F -117.66 or -117.69.

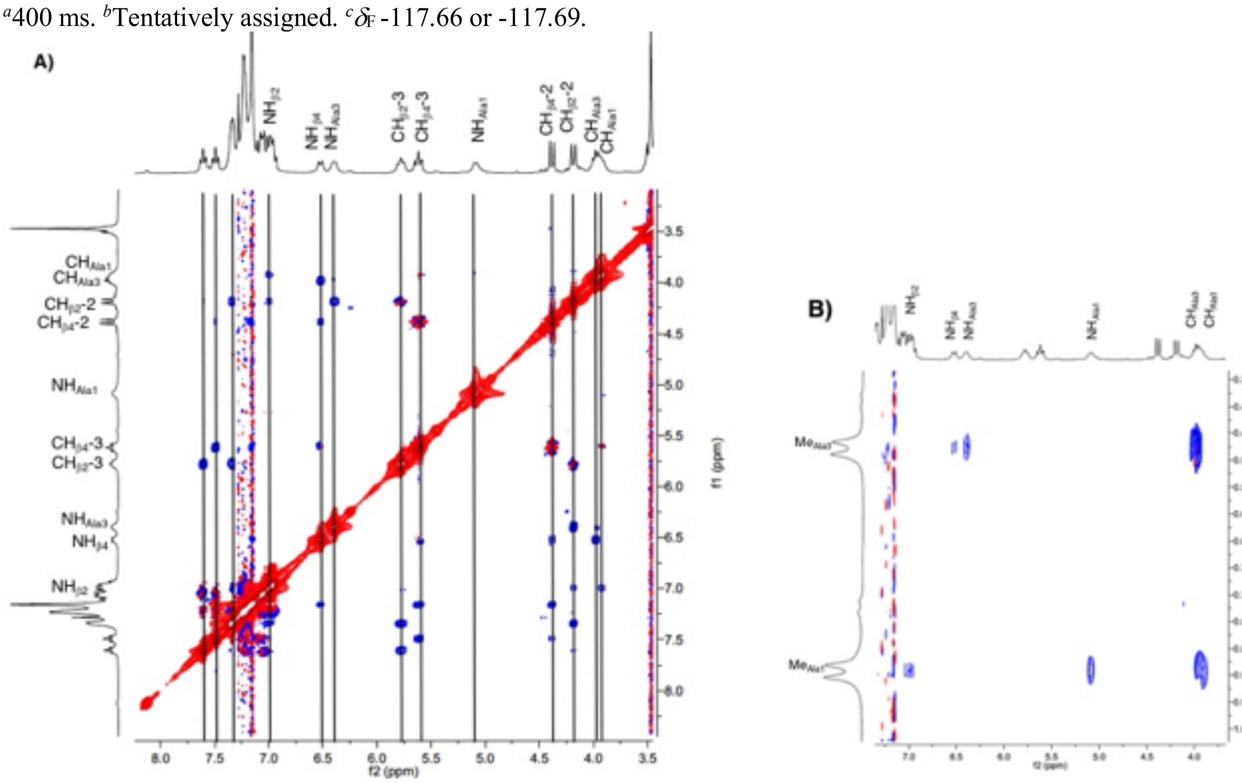

Figure S2. Tetrapeptide **7**: A) CH/NH Noe region. B)Me/CH and Me/NH Noe region; ($CDCl_3$, 31 mM, 500 MHz 400 ms at 300 K).

Table TS2. Nuovo Tetrapeptide **8** ($CDCl_3$, 10 mM, 293 K, 400 MHz)

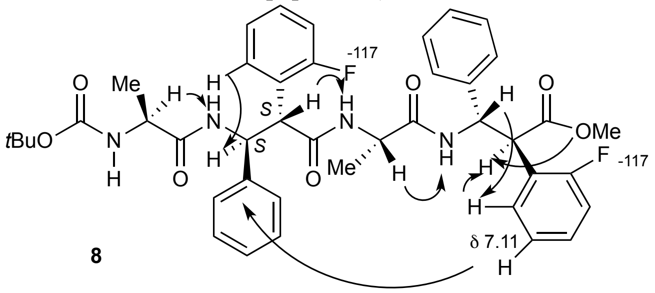

AA	atom		^{1}H	Molteplicity<br>*J* (Hz)	^{13}C	Noesy CH[a]<br>Fδ
**Ala-1**	CO				171.2	
	CH		3.95	brs	50.4 br	$NH_{\beta 2}$ (m)
	Me		1.12		17.8	CH_{Ala1}(s), NH_{Ala1}(w)
	NH		4.83	brs		Me_{Ala1}(w)
	Boc	Me	1.42	brs	28.2	
		C			80.1	
		CO			155.5	
***Beta*-2**	CO				169.8	
	2		4.24	d, *J* 8.3	49.1	NH_{Ala3}(s), Ar(7.28, m)
	3		5.63	t *J* 8.3, 9.0	54.3 brs	Ar(7.32,s)
	NH		7.37	overlapped		
	Ar_F<br><br><br><br><br><br>Ph		7.32_{F-6}<br>7.04_{F-5}<br>7.26_{F-4}<br>7.01_{F-3}<br><br><br>7.30-7.22		130.5_{F-6} (brs)<br>124.3_{F-5} (brs)<br>129.3_{F-4} (*J* 8.9)<br>115.0_{F-3} (*J* 14.2)<br>162.2_{F2} (*J* 251.1)<br>122.8_{F1} (*J* 12.5)<br>128.5-128.3<br>139.2(q, brs)	H_{F-6} : Ar(7.04,s), $H_{\beta 4}$-3(m)<br>H_{F-5}: Ar(7.32,s)<br><br>H_{F-3}: Ar(7.29s)<br><br>δ_F[c]
**Ala-3**	CO				171.0	
	CH		4.09	brs	49.4	Me_{Ala3}(s), $NH_{\beta 4}$(s)
	Me		0.97	d, *J* 6.7	17.3	CH_{Ala3} (s), NH_{Ala3}(vw)
	NH		6.36	brs		$H_{\beta 2}$-2 (s)
***Beta*-4**	CO				171.0	
	2		4.47	d, *J* 10.3	48.8 (J_{CF} 1.6)	OMe (w), $NH_{\beta 4}$(m), Ar(7.28s, 7.44m)
	3		5.57	dd, *J* 10.3, 9.0	54.9	$NH_{\beta 4}$ (m), Ar(7.28,s), Ar_{F-6}(7,44,s)
	NH		6.69	d, *J* 8.4		CH_{Ala3}(s), $H_{\beta 4}$-2 (s), $H_{\beta 4}$-3(m), Ar(7.27,m)
	Ar_F<br><br><br><br><br><br>Ph		7.44_{F-6}<br>7.11_{F-5}<br>7.27_{F-4}<br>7.05_{F-3}<br><br><br>7.30-7.22		129.9_{F-6} (*J* 2.5)<br>124.5_{F-5} (*J* 3.3)<br>129.7_{F-4} (*J* 8.7)<br>115.3_{F-3} (*J* 15.5)<br>162.2_{F2} (*J* 251.1)<br>122.5_{F1} (*J* 13.6)[b]<br>128.5-128.3<br>139.0(q)	H_{F-6} : $H_{\beta 4}$-2(m), $H_{\beta 4}$-3(s), Ar(7.11,s)<br>H_{F-5}: Ar(7.44, s), Ph(7.31,m)<br><br>δ_F[c]
	OMe		3.55	s	52.2	$H_{\beta 4}$-2 (w)

[a]300 ms; [b]A correlation between this carbon with $H_{\beta 4}$-3 in the HMBC experiment confirms the correct assignment.

[c]δ_F-117.5 or -117.77.

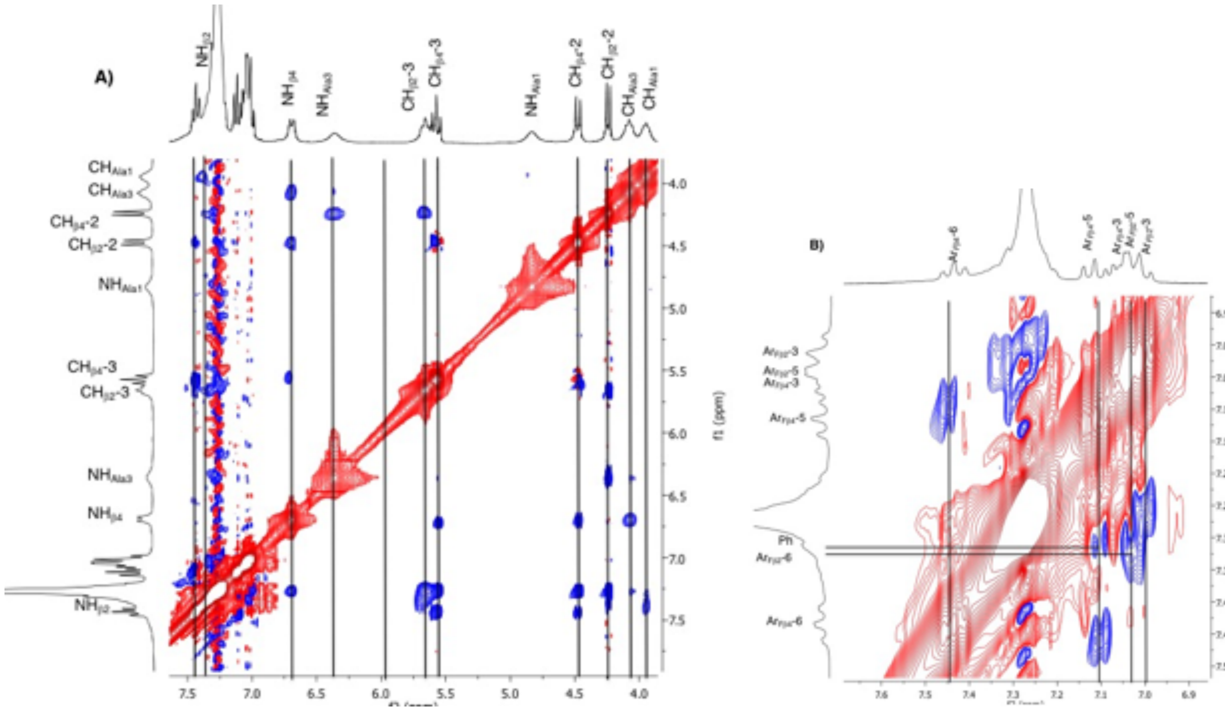

Figure S3. Tetrapeptide **8**: A) CH/NH Noe region; B) Aromatic Noe region ($CDCl_3$, 10 mM, 293 K, 400 MHz, 300 ms at 300 K).

Table TS3. Hexapeptide **11** ($CDCl_3$, 10 mM, 293 K, 300 MHz)

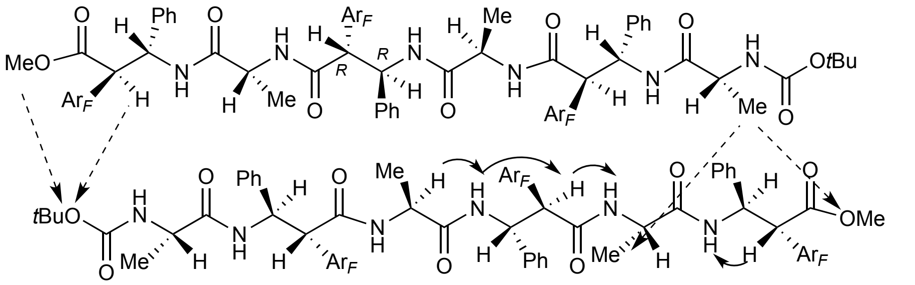

11

AA	atom		^{1}H	Molteplicity *J* (Hz)	^{13}C	H (Noesy/Roesy)[a] F_δ (Hoesy)
**Ala-1**	CO				172.1	
	CH		4.05		49.7	Me_{Ala1}(vs), OMe(vw)
	Me		0.77	*J* 7.0	19.6	CH_{Ala1}(s), Me_{Ala5}(m), Boc(w),
	NH		5.49	*J* 6.8		
	Boc	Me	1.40		28.8	$H_{\beta6}$-2(m), OMe(w), Arom (7.19s, 7.10m)
		C			79.8	
		CO			155.8	
Beta-2	CO				171.0	
	2		4.55	d, *J* 9.4	50.7(J_{CF} 2.2)	
	3		5.97	t, *J* 11.1	53.9	$H_{\beta2}$-2(w), Arom(7.61vs)
	NH		7.49	overl.		
	Ar_F		$7.61_{F\text{-}6}$ $6.97_{F\text{-}5}$ $7.16_{F\text{-}4}$ $6.77_{F\text{-}3}$	Overl.	b	F_6: Ar(6.97vs)
	Ph		7.43 7.21 7.15		128.2 127.5[c] 129.9[c]	$H_{\beta2}$-3(vs) F_δ[e]

				[d]	
**Ala-3**	CO			171.4	
	CH	4.19		48.9	Me_{Ala3}(m), $NH_{\beta 4}$(m)
	Me	0.32	*J* 6.9	18.8	H_{Ala3}(m)
	NH	7.43	overl.		
***Beta*-4**	CO			171.1	
	2	4.27	d,*J*10.7	51.3	Ar(6.84s), $NH_{\beta 4}$(m),
	3	5.92	t *J*11.4	53.6	$H_{\beta 4}$-2(w), Ph(7.16vs), $Ar_{\beta 4}$(7.48m)
	NH	6.88	overl.		H_{Ala3}(m), $H_{\beta 4}$-2(m)
	Ar_F<br><br><br><br>Ph	7.48$_{F\text{-}6}$<br>6.99$_{F\text{-}5}$<br>7.14$_{F\text{-}4}$<br>6.84$_{F\text{-}3}$<br>7.23-7.07		[b]<br><br><br><br>128.8-127.5 [d]	F_6: Ar(6.99vs), $H_{\beta 4}$-3(s)<br>F_5: Ar(7.50s)<br><br>F_δ[e]
**Ala-5**	CO			171.0	
	CH	4.09		48.7	Me_{Ala5}(s)
	Me	0.35	*J* 6.5	18.8	H_{Ala5}(s), Me_{Ala1}(m)
	NH	6.85	overl.		
***Beta*-6**	CO			171.0	
	2	4.41	*J*11.2	48.9	Boc(m), Arom (7.22s), $H_{\beta 6}$-3(),<br>$NH_{\beta 6}$(vw)
	3	5.69	t*J*10.6	54.1	$H_{\beta 6}$-2(s), $NH_{\beta 6}$(w), $Ar_{\beta 6}$(7.49w)<br>Ph(7.21,vs)
	NH	6.76	overl.		
	Ar_F<br><br><br><br>Ph	7.49$_{F\text{-}6}$<br>6.95$_{F\text{-}5}$<br>7.18$_{F\text{-}4}$<br>6.91$_{F\text{-}3}$<br>7.23-7.07	 overl.	[b]<br><br><br><br>128.8-127.5 [d]	$H_{\beta 6}$-3(w)<br><br>F_δ: -117.68 ($H_{\beta 6}$-2s, $Ar_{F\text{-}5}$m, 7.49)
	OMe	3.44		52.4	Boc(w), H_{Ala3}(vw)

[a]NOESY (500 or 300 ms), ROESY (200 ms). [b]δ_C: $Ar_{F\text{-}6}$129.5-129.9 region; $Ar_{F\text{-}5}$124.9-124.6 region; $Ar_{F\text{-}4}$129.0-129.5 region; $Ar_{F\text{-}3}$ 115.7 (*J* 23.0) or 115.6 (*J* 21.9) or 115.5 (*J* 22.0); $Ar_{F\text{-}2}$ 160.5 (*J* 246.6) or 160.4 (*J* 246.6); $Ar_{F\text{-}1}$ 123.9 (*J* 13.8) or 123.6 (*J* 13.8) or 122.9 (*J* 14.0). [c]Tentatively assigned. [d]δ_C: Ph_4 140.4 or 139.9 or 139.6. [e]δ_F -116.03 (brs) or -114.79 (brs).

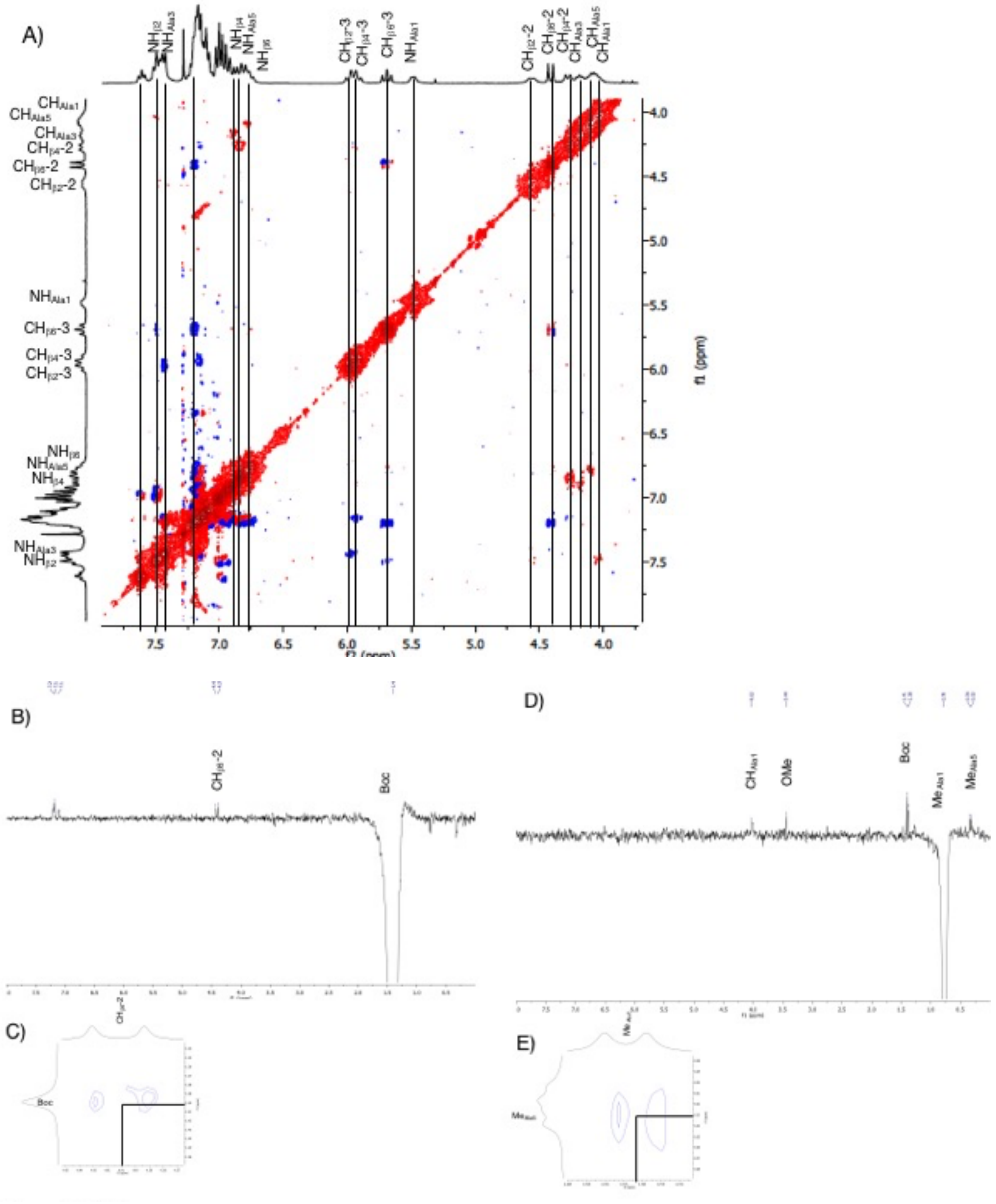

Figure S4. Hexpeptide **11**: A) CH/NH/Aromatic NOESY region; B) Row of Boc; C) NOESY zoom of Boc/$CH_{\beta6}$-2; D) Row of Me_{Ala1}; E) NOESY zoom of Me_{Ala1}/ Me_{Ala5} ($CDCl_3$, 10 mM, 293 K, 300 MHz, 300 ms).

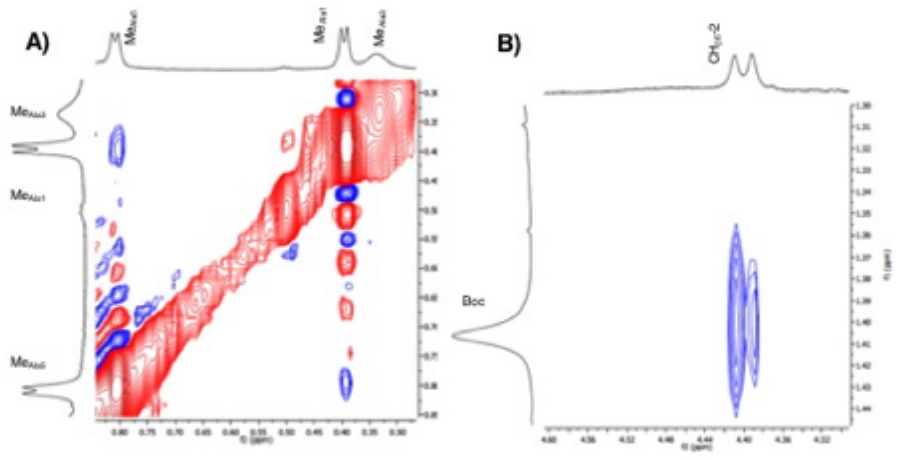

Figure S5. ROESY zoom of hexpeptide **11**: A) Me region; B) Boc/CH$_{\beta 6}$-2 region (CDCl$_3$, 1.5 mM, 300 K, 600 MHz, 300 ms).

Table TS4 Hexapeptide **11** (DMSO-d$_6$, 10 mM, 293 K, 300 MHz).

AA	atom		^{1}H	Molteplicity *J* (Hz)	^{13}C	H (Roesy)[a]
**Ala-1**	CO				171.9	
	CH		3.75		47.8	NH$_{\beta 2}$(s), Me$_{Ala1}$(vs)
	Me		0.57	d, *J* 6.9	18.6	NH$_{\beta 2}$(w), NH$_{Ala1}$(m), CH$_{Ala1}$(vs),
	NH		6.48	d, *J* 8.2		CH$_{Ala1}$(vw), Me$_{Ala1}$(m)
	Boc	Me	1.28		28.6	
		C			78.2	
		CO			155.0	
***Beta*-2**	CO				169.0	
	2		4.27	d, *J* 11.0	48.9	NH$_{Ala3}$(vs), NH$_{\beta 2}$(w), Ph(7.35s)
	3		5.45	dd, *J* 10.5, 9.6	54.4	Arom(7.53vs, 7.35m)
	NH		8.13	*J* 9.6		CH$_{Ala1}$(s), Me$_{Ala1}$(w)
	Ar$_F$<br><br><br><br><br>Ph		7.53$_{F\text{-}6}$<br>7.03$_{F\text{-}5}$<br>7.19$_{F\text{-}4}$<br>7.04$_{F\text{-}3}$<br><br>[c]		130.2<br>124.8(brs)<br>overl.<br>115.1 (*J* 22.7)<br>[b]<br>[d]	F$_6$: H$_{\beta 2}$–2(s)
**Ala-3**	CO				170.9	
	CH		3.81		47.6	Me$_{Ala3}$(s), NH$_{\beta 4}$(s)
	Me		0.08	d *J* 7.2	18.1	H$_{Ala3}$(s), NH$_{\beta 4}$(w), NH$_{Ala5}$(vs)
	NH		7.82	d *J* 8.0		H$_{Ala3}$(w), H$_{\beta 2}$-2(s)
***Beta*-4**	CO				169.1	
	2		4.20	d *J* 11.4	48.8	Ar(7.25s), NH$_{\beta 4}$(m), NH$_{Ala5}$(vs)
	3		5.35	dd *J* 11.4,<br>10.0	54.4	Ar(7.49vs,7.25m)
	NH		8.12	*J* 10.0		H$_{Ala3}$(s), H$_{\beta 4}$-2(m), Me$_{Ala3}$(w)
	Ar$_F$<br><br><br><br><br><br>Ph		7.49$_{F\text{-}6}$<br>6.96$_{F\text{-}5}$<br>7.17$_{F\text{-}4}$<br>6.98$_{F\text{-}3}$<br><br><br>[c]		130.1<br>124.1 (brs)<br>Overl.<br>115.1 (*J* 22.7)<br>124.7(*J*10.5)<br>[b]<br>[d]	F$_6$: H$_{\beta 3}$-3(vs)
**Ala-5**	CO				171.0	
	CH		3.76		48.7	NH$_{\beta 6}$(vs), Me$_{Ala5}$(s)

	Me	0.07	d J 7.02	18.3	NH$_{Ala5}$(m), NH$_{\beta6}$(w), Ph(7.25w), CH$_{Ala5}$(s)
	NH	7.91	J 7.9		H$_{\beta4}$-2(vs)
***Beta*-6**	CO			170.9	
	2	4.28	J 11.4	49.6	Arom (7.29s) NH$_{\beta6}$(m)
	3	5.50	dd J 11,4, 9.7	52.9	Ar$_{\beta6}$(7.39s), Ph(7.29m)
	NH	8.28	J 9.7		H$_{Ala5}$(vs), H$_{\beta6}$-2(m), Me$_{Ala5}$(w)
	Ar$_F$<br><br><br><br><br><br>Ph	7.39$_{F\text{-}6}$<br>7.10$_{F\text{-}5}$<br>7.28$_{F\text{-}4}$<br>7.12$_{F\text{-}3}$<br><br><br>^{c}	overl.	129.9<br>124.7 brs<br>130.1<br>115.7 (J 23.5)<br>123.0(J15.7)<br>^{b}<br>^{d}	H$_{\beta6}$-3(s)
	OMe	3.34		52.4	

[a]200 ms. Ph: [c]δ$_C$: 128.9-127.4 Ar$_{F\text{-}2}$ 160 region

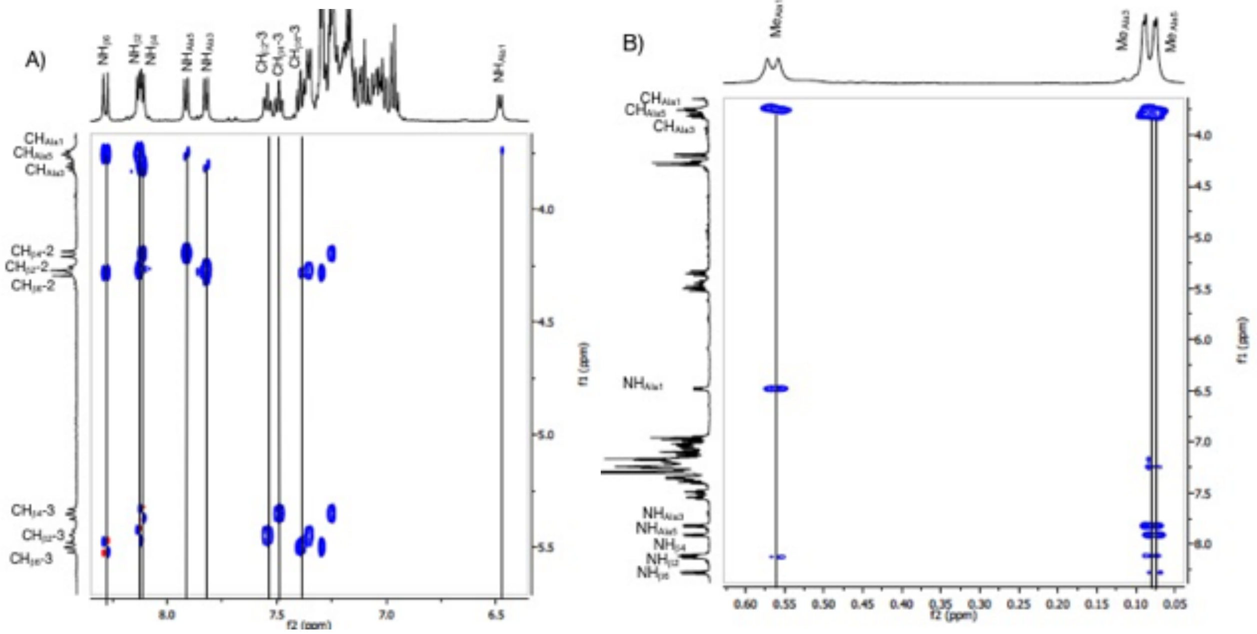

Figure S6. Hexpeptide **11**: A) CH/NH/Aromatic Noe region; B) Me ROESY region (DMSO-d$_6$, 10 mM, 293 K, 300 MHz, 200 ms at 300 K).

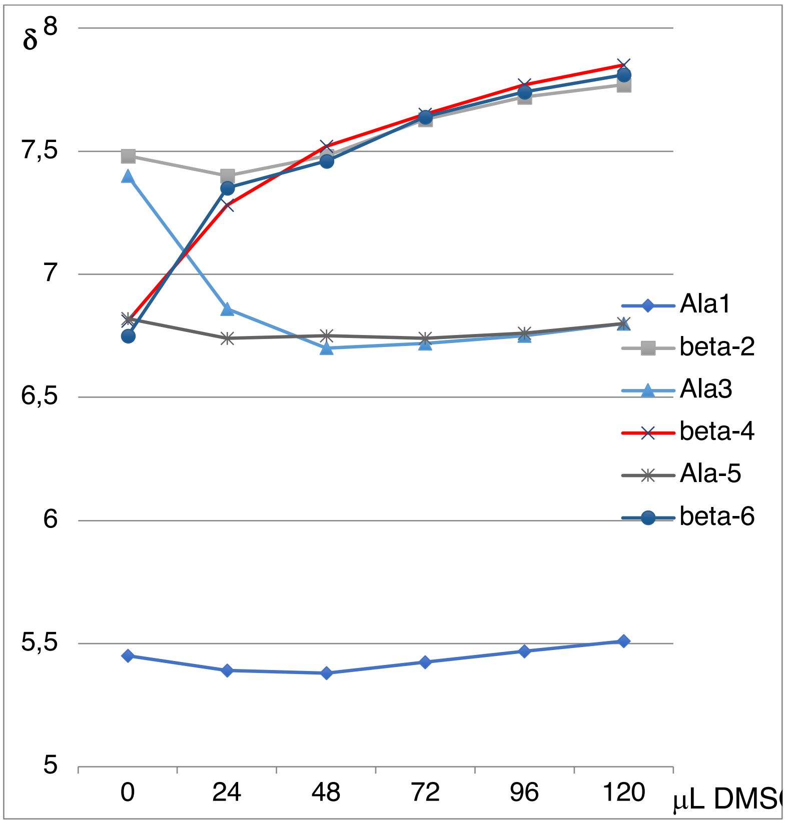

Figure S7. DMSO-d$_6$ titrations of peptide **11** in $CDCl_3$ (10 mM; 500 MHz).

Table TS5. Hexapeptide **12** ($CDCl_3$, 6.2 mM, 293 K, 500 MHz).

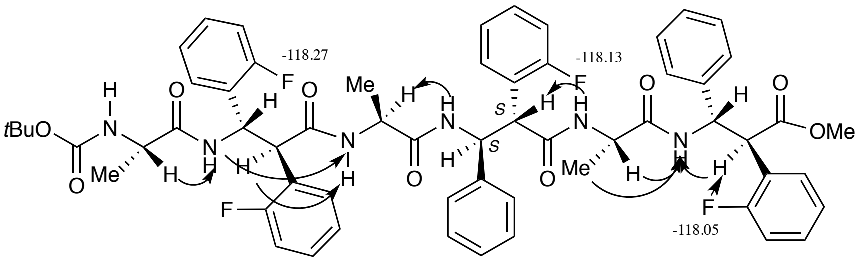

12

AA	atom		^{1}H	Molteplicity *J* (Hz)	^{13}C	H (Noesy)[a] F$_\delta$ (Hoesy)
**Ala-1**	CO				172.9	
	CH		4.22	brs	51.2	$NH_{\beta 2}$(s), NH_{Ala1}(vvw), Me_{Ala1}(s)
	Me		1.36	d, *J* 6.2	17.5	NH_{Ala1}(m), CH_{Ala1}(s)
	NH		5.12	brs		Me_{Ala1}(m)
	Boc	Me	1.50		28.4	
		C			80.3	
		CO			155.1	
Beta-2	CO				170.3	
	2		4.37	d, *J* 6.4	47.3	$H_{\beta 2}$-3(s), NH_{Ala3}(s)
	3		5.60	brt	55.3	$H_{\beta 2}$-2(s), $NH_{\beta 2}$(vvw), Arom (7.02 vs)
	NH		8.09	d, *J* 8.2		CH_{Ala1} (s), Ar(7.03w), NH_{Ala3}(vw)
	Ar$_F$		7.27$_{F-6}$<br>6.86$_{F-5}$<br>7.22$_{F-4}$<br>7.10$_{F-3}$<br>-<br>-	Overl.<br>Overl.	129.7 $_{F-6}$<br>122.9 $_{F-5}$ (*J* 6.6)<br>131.7$_{F-4.}$<br>114.7 $_{F-3}$ (*J* 22.4)<br>165.1$_{F2}$ (*J* 245.0)<br>122.8 $_{F1}$ (*J* 11.1)	F$_\delta$: -118.27 ($H_{\beta 2}$-2,s; 7.14,s)

	Ph	[c]	Overl.	[d]	
**Ala-3**	CO			173.6	
	CH	3.85	br	50.9	Me_{Ala3}(s), $NH_{\beta4}$(s)
	Me	1.20	*J* 5.6	17.0	H_{Ala3}(s), NH_{Ala3}(m)
	NH	7.56	brs		Me_{Ala3}(s), $H_{\beta2}$-2(s)
***Beta*-4**	CO			169.5	
	2	4.54	br	47.3	$H_{\beta4}$-3(s), NH_{Ala5}(7.26m), Ar(7.04m),
	3	5.45	dd, *J* 9.7, 6.0	56.3	$NH_{\beta4}$(w), $H_{\beta4}$-2(s), Ar(7.04s)
	NH	8.18	d, *J* 9.7		H_{Ala3}(m), Ar(7.06s, 7.24w)
	Ar_F<br><br><br><br><br><br>Ph	7.24_{F-6}<br>6.89_{F-5}<br>7.26_{F-4}<br>7.11_{F-3}<br>-<br>-<br>[c]	Overl.<br>Overl.<br><br><br><br><br>Overl.	131.9_{F-6}<br>123.4_{F-5}<br>129.7_{F4}<br>114.8_{F-3} (*J*23.5)<br>165.1_{F2}(*J* 245.0)<br>122.8_{F1} (*J* 11.1)<br>[d]	F_6: $H_{\beta4}$-2(m)<br>F_5: NH_5-2(s) (7.24s)[b]
**Ala-5**	CO			172.4	
	CH	4.40	m	49.2	Me_{Ala5}(vs), $NH_{\beta6}$(m)
	Me	1.01	d, *J* 7.1	16.4	H_{Ala5}(vs), $NH_{\beta6}$(m), Ar(7.04w,6.95 vvw)
	NH	7.24	overl.		$H_{\beta4}$-2(w)[b]
***Beta*-6**	CO			171.1	
	2	4.49	d, *J* 9.4	48.8	$H_{\beta6}$-3(s), $NH_{\beta6}$(s), Ar(7.35 w; 7.13s)
	3	5.57	t, *J* 8.6, 9.4	55.3	$NH_{\beta6}$(vw), $H_{\beta6}$-2(s), Ar(7.13vs, 7.34m)
	NH	6.58	d, *J* 8.6		Me_{Ala5}(m), H_{Ala5}(w), $H_{\beta6}$-2(w), $H_{\beta6}$-3(w), Ar(7.13 m)
	Ar_F<br><br><br><br><br><br>Ph	7.35_{F-6}<br>7.13_{F-5}<br>7.08_{F-4}<br>7.04_{F-3}<br>-<br>-<br>[c]	 <br><br><br><br><br>Overl.	129.9_{F-6} (*J* 2.0)<br>124.7_{F-5}<br>131.6_{F-4}<br>115.4_{F-3} (*J* 21.3)<br>165.2_{F2} (*J* 245.3)<br>122.3_{F1} (*J* 13.6)<br>[d]	F_6: $H_{\beta6}$-3(vs), $H_{\beta6}$-2(m)<br>F_5: $H_{\beta6}$-3(vs), $H_{\beta6}$-2(s), NH_6(m)<br><br><br>F_δ: -118.05 ($H_{\beta6}$-2vs; 7.14s, 7.10vs, 7.17w)
	OMe	3.56	s	52.3	

[a]500 ms. [b]Tentatively assigned. [c]δ_H 7.02-7.30. [d]δ_C 127.3 (127.4,127.5), 127.7 (127.72, 128.3), 128.6 (128.7, 128.9), 139.2 (q).

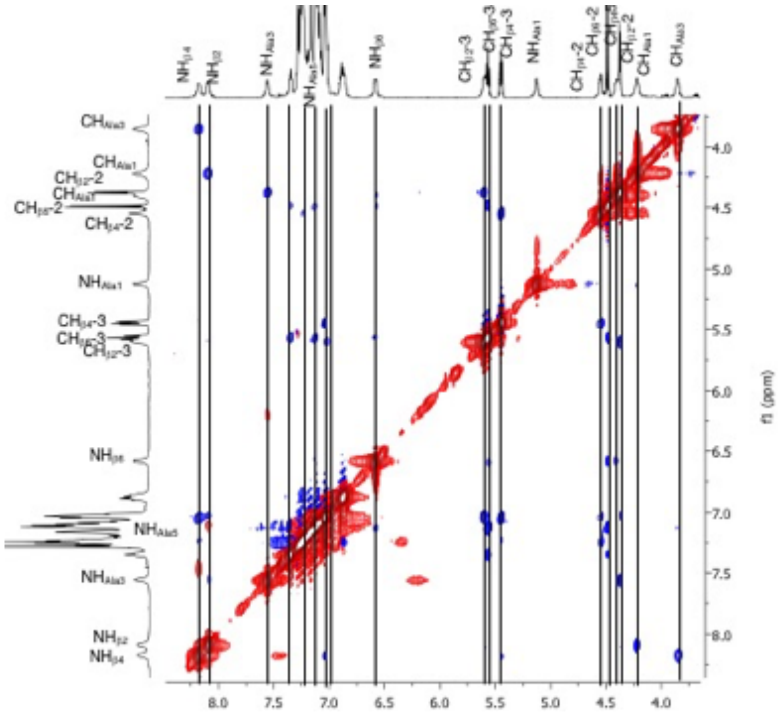

Figure S8. Hexpeptide **12**: CH/NH/Aromatic Noe region (CDCl3, 6.2 mM, 293 K, 500 MHz)

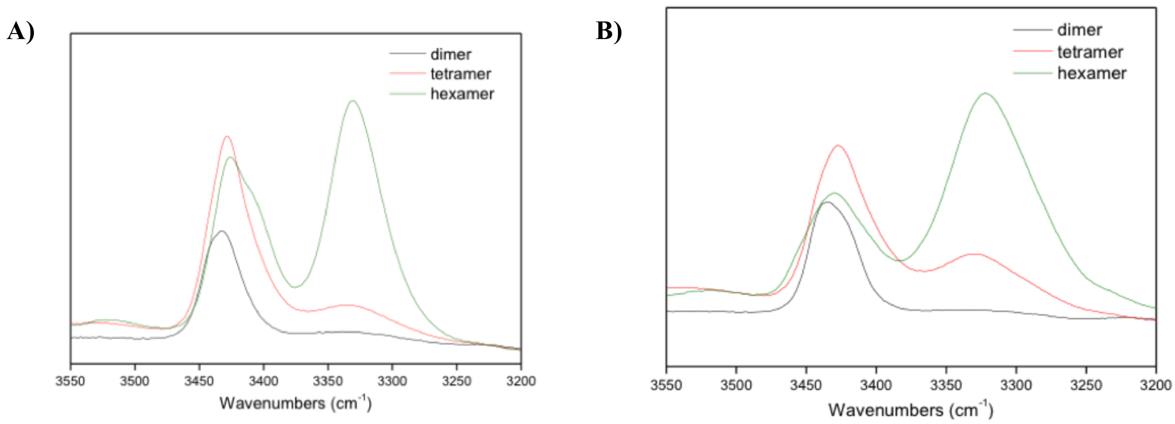

Figure S9. FTIR absorption spectra (N-H stretching region) of peptides **1,7,11** (A) and **2,10,12** (B) in $CDCl_3$ solution. Peptide concentration: $1x10^{-3}$ M.

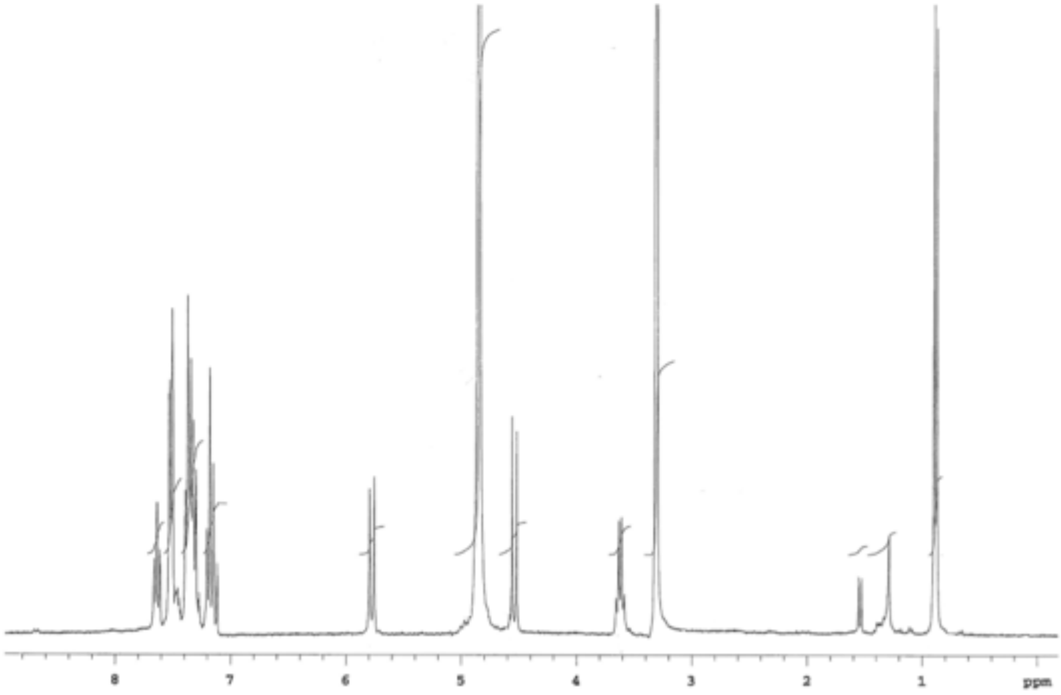

Figure S10: ^{1}H NMR spectra of H-*S*-Ala-β-2*R*,3*R*-Fpg-OH HCl

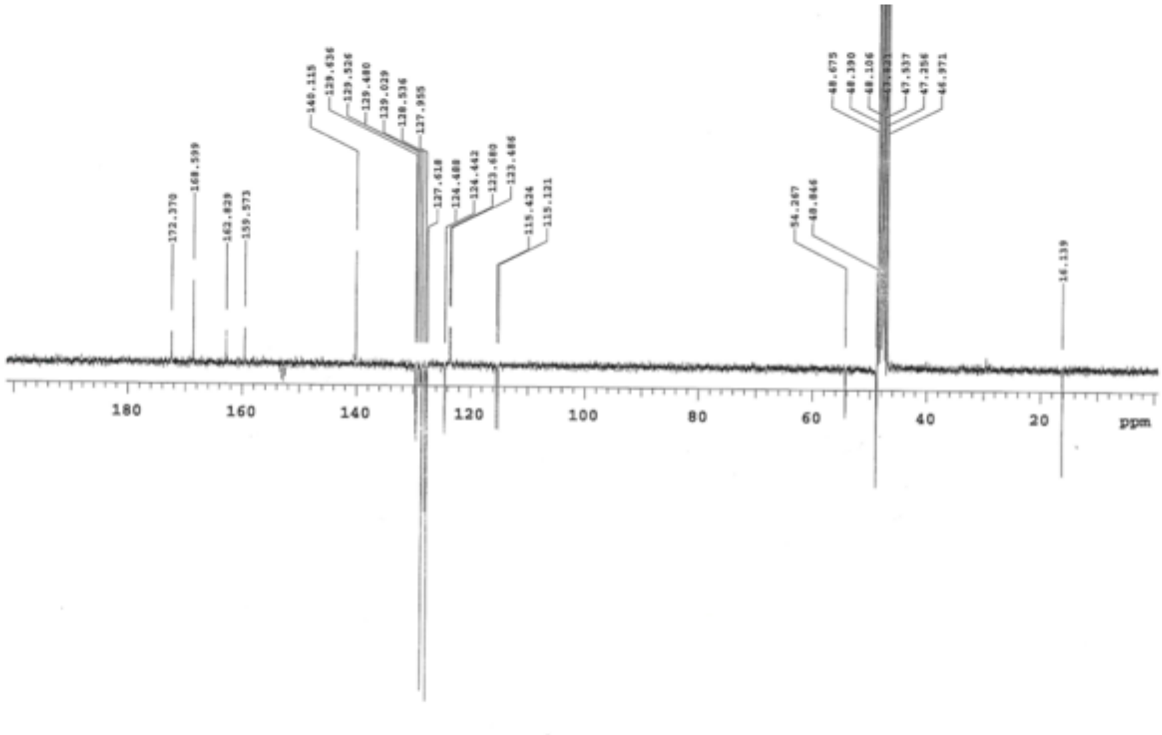

Figure S11: ^{13}C NMR spectra of H-*S*-Ala-β-2*R*,3*R*-Fpg-OH HCl

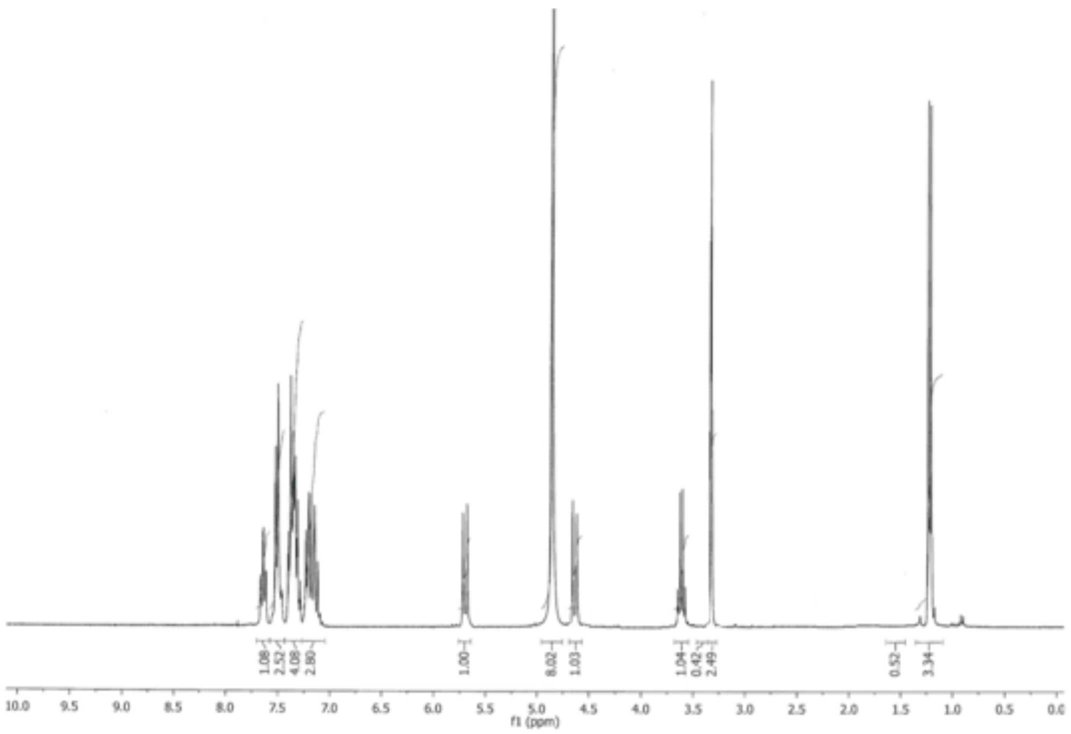

Figure S12: ^{1}H NMR spectra of H-*S*-Ala-β-2*S*,3*S*-Fpg-OH HCl

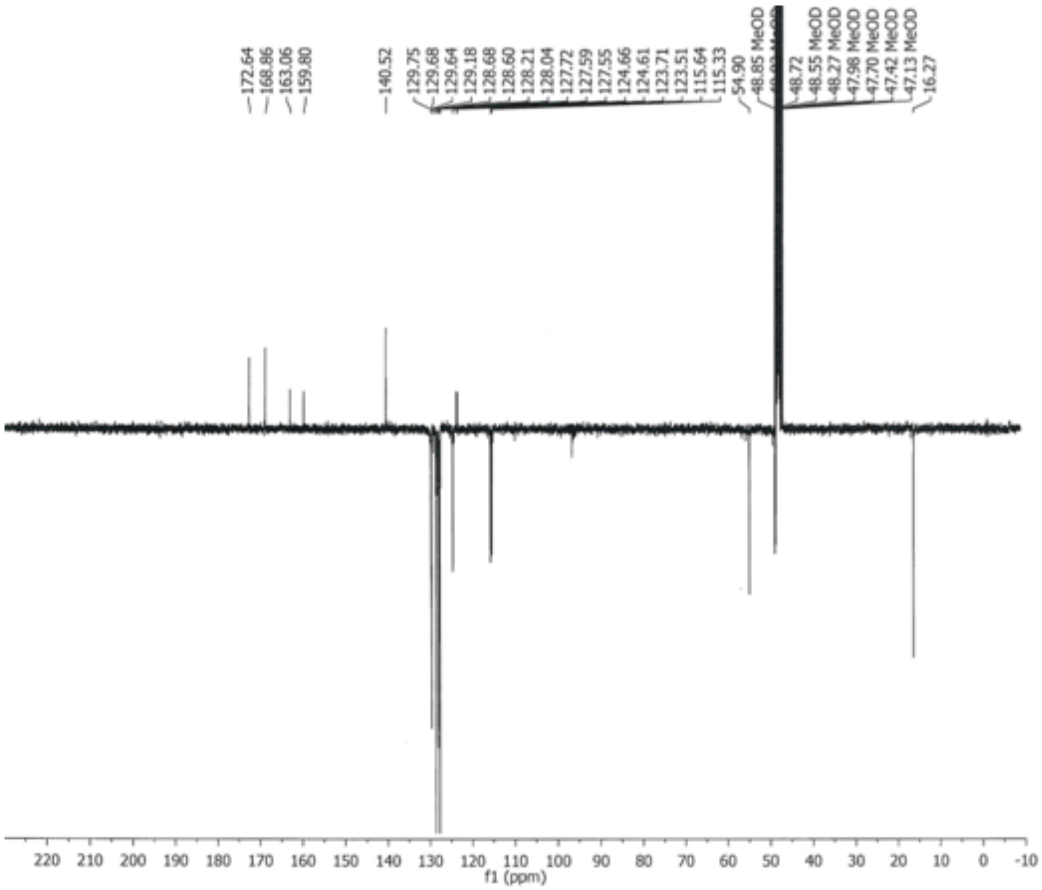

Figure S13: ^{13}C NMR spectra of H-*S*-Ala-β-2*S*,3*S*-Fpg-OH HCl

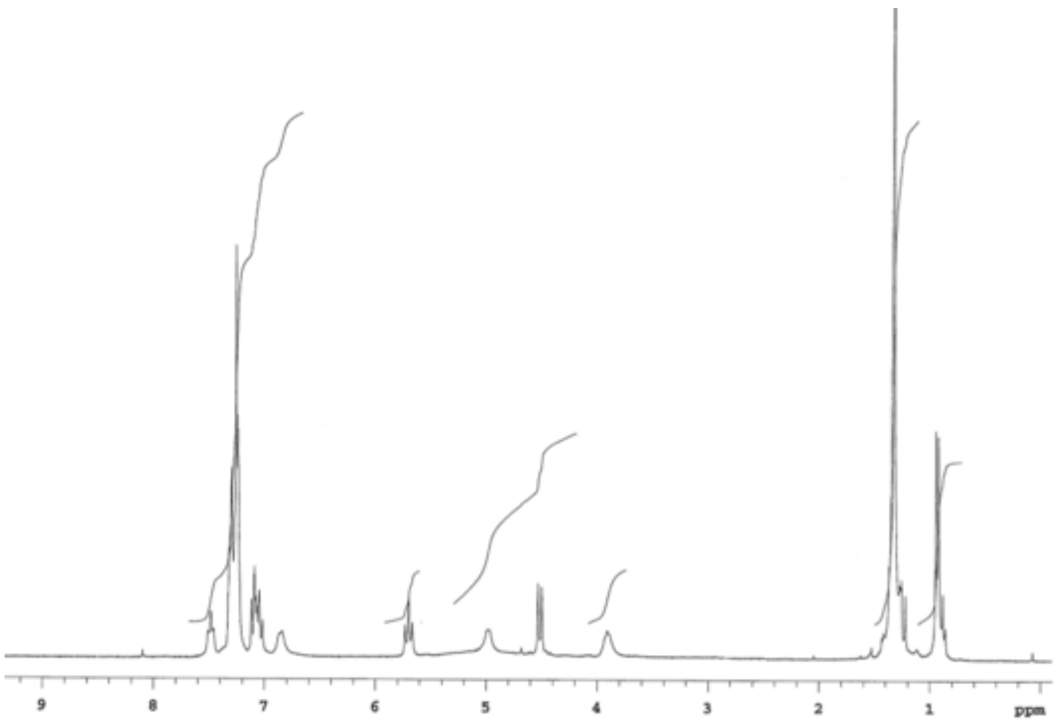

Figure S14: ^{1}H NMR spectra of Boc-*S*-Ala-β-2*R*,3*R*-Fpg-OH (**3**)

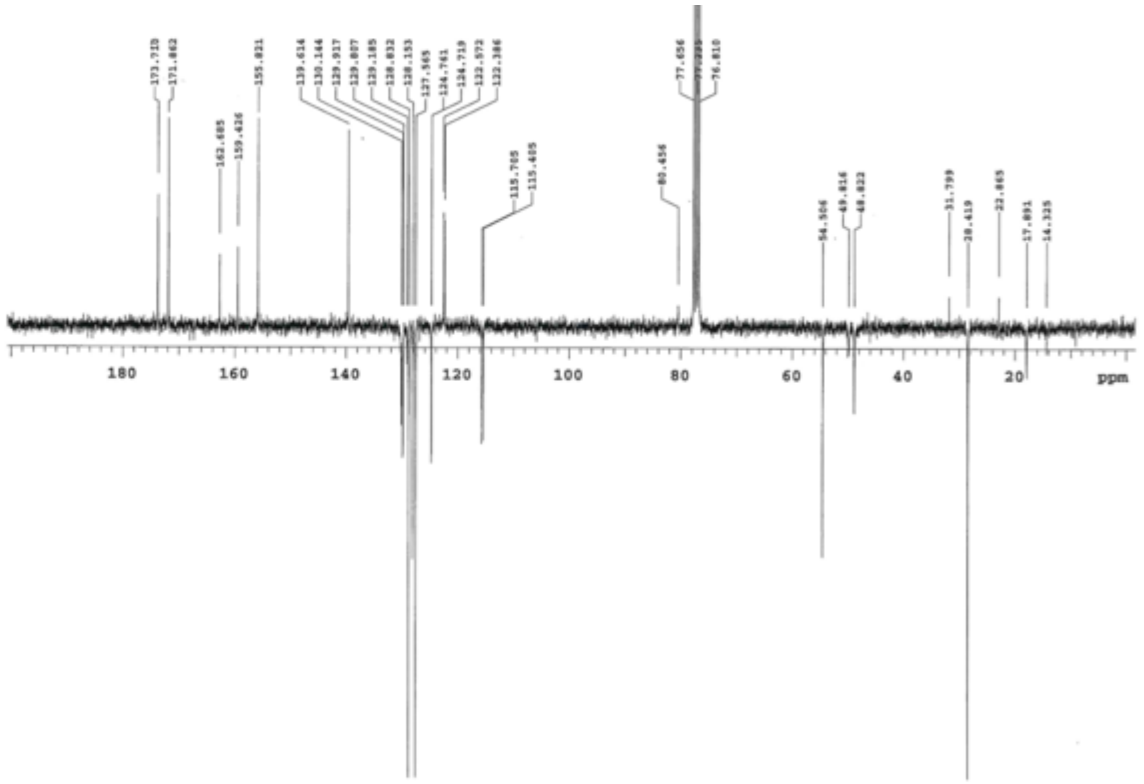

Figure S15: ^{13}C NMR spectra of Boc-*S*-Ala-β-2*R*,3*R*-Fpg-OH (**3**)

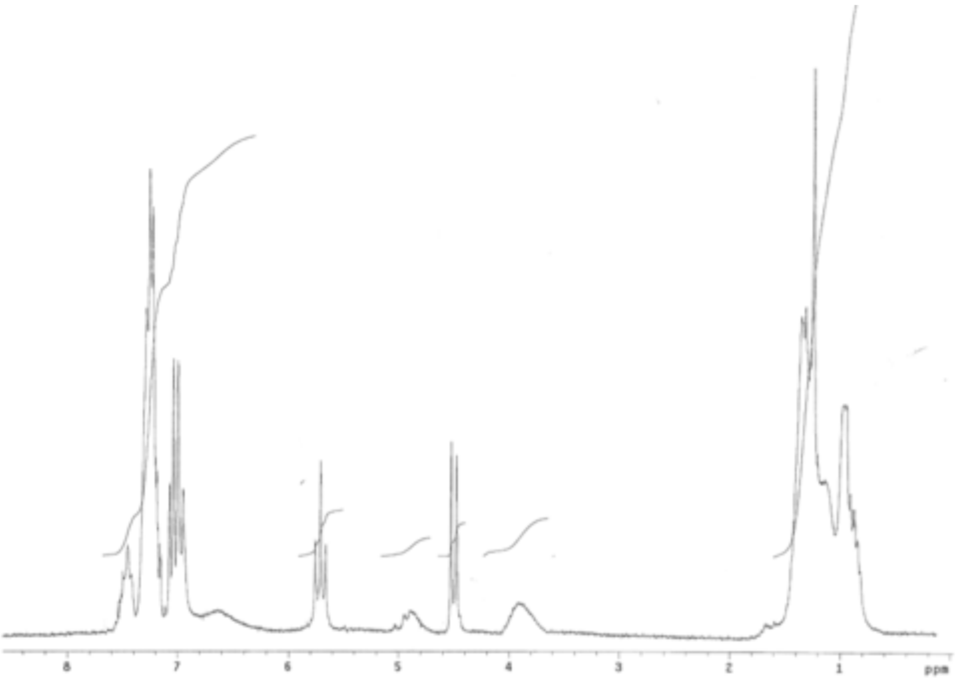

Figure S16: ^{1}H NMR spectra of Boc-*S*-Ala-β-2*S*,3*S*-Fpg-OH (**4**)

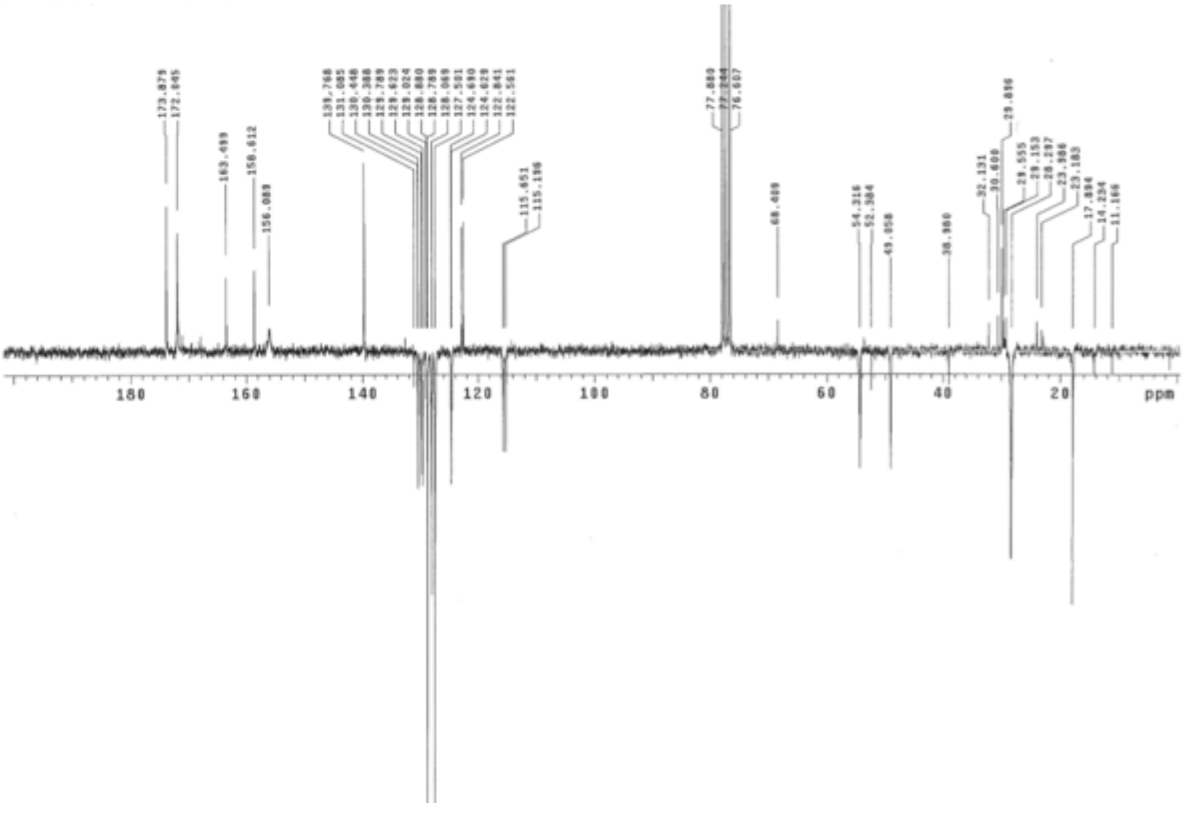

Figure S17: ^{13}C NMR spectra of Boc-*S*-Ala-β-2*S*,3*S*-Fpg-OH (**4**)

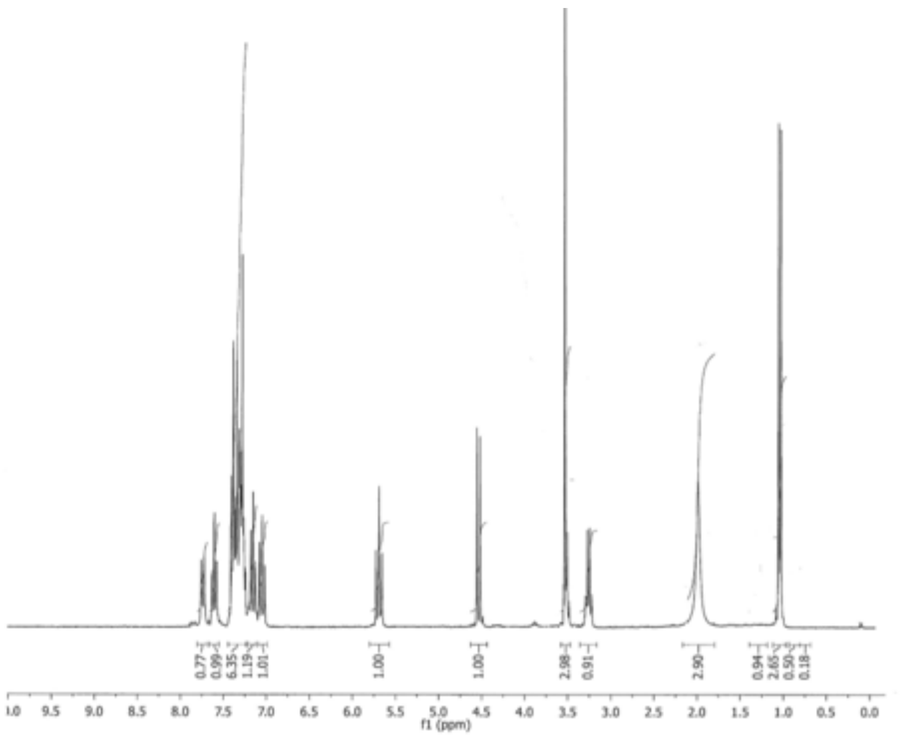

Figure S18: ^{1}H NMR spectra of H-*S*-Ala-β-2*R*,3*R*-Fpg-OMe (**5**)

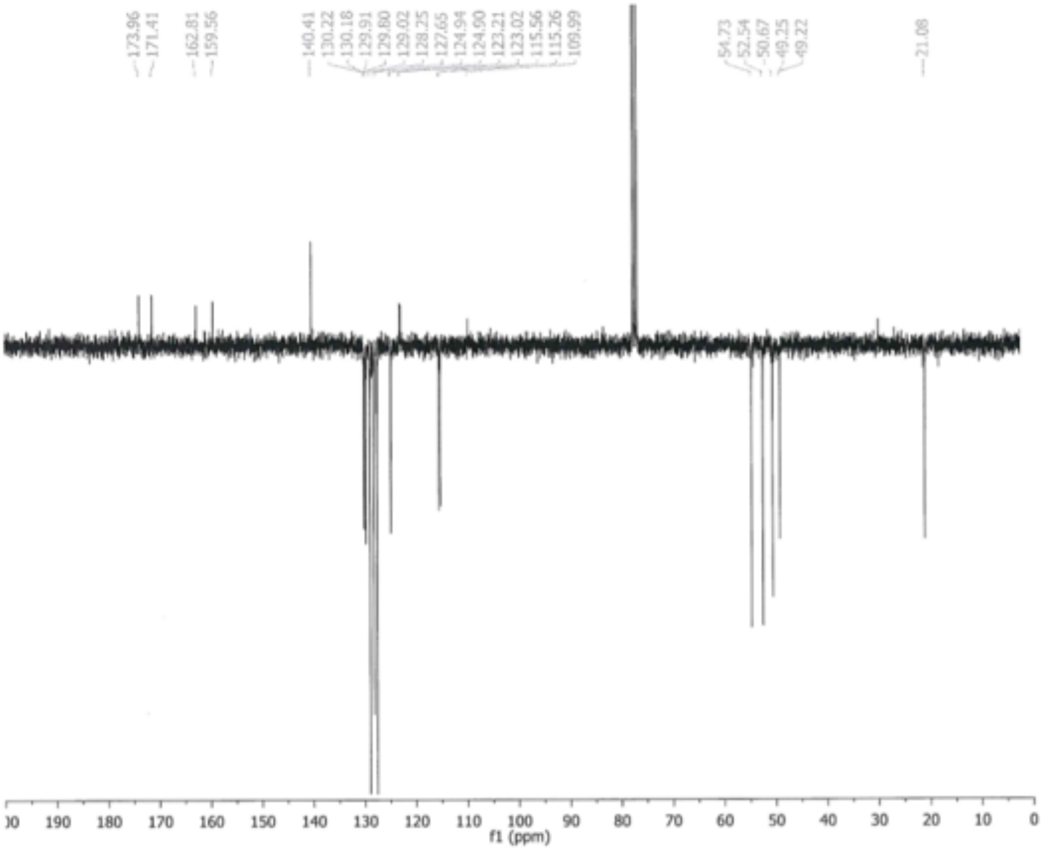

Figure S19: ^{13}C NMR spectra of H-*S*-Ala-β-2*R*,3*R*-Fpg-OMe (**5**)

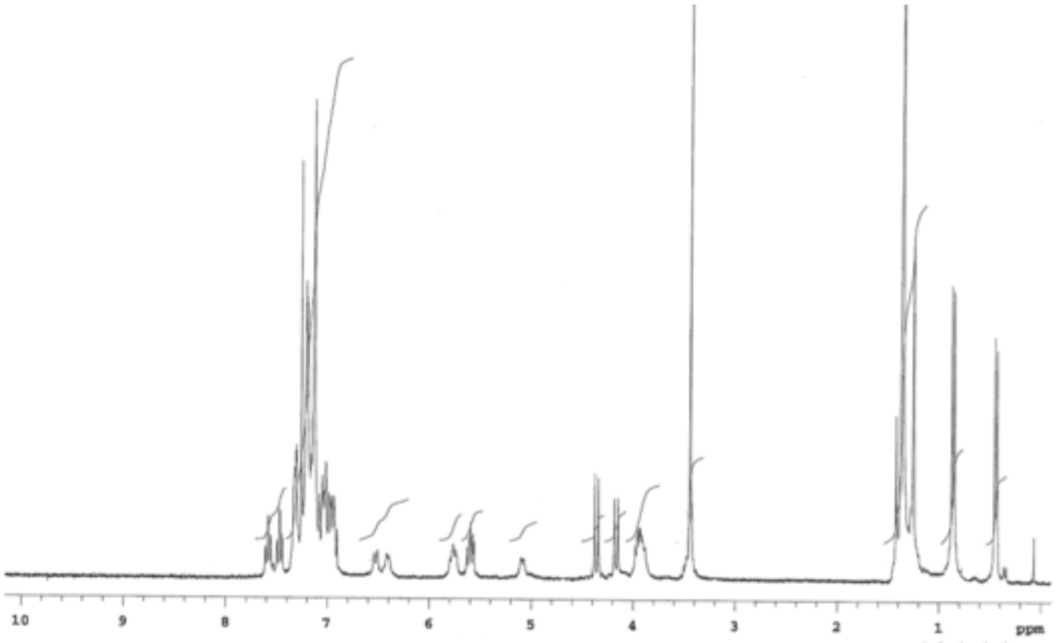

Figure S20: ^{1}H NMR spectra of Boc-[*S*-Ala-β–2*R,3R*-Fpg]$_2$-OMe (**7**)

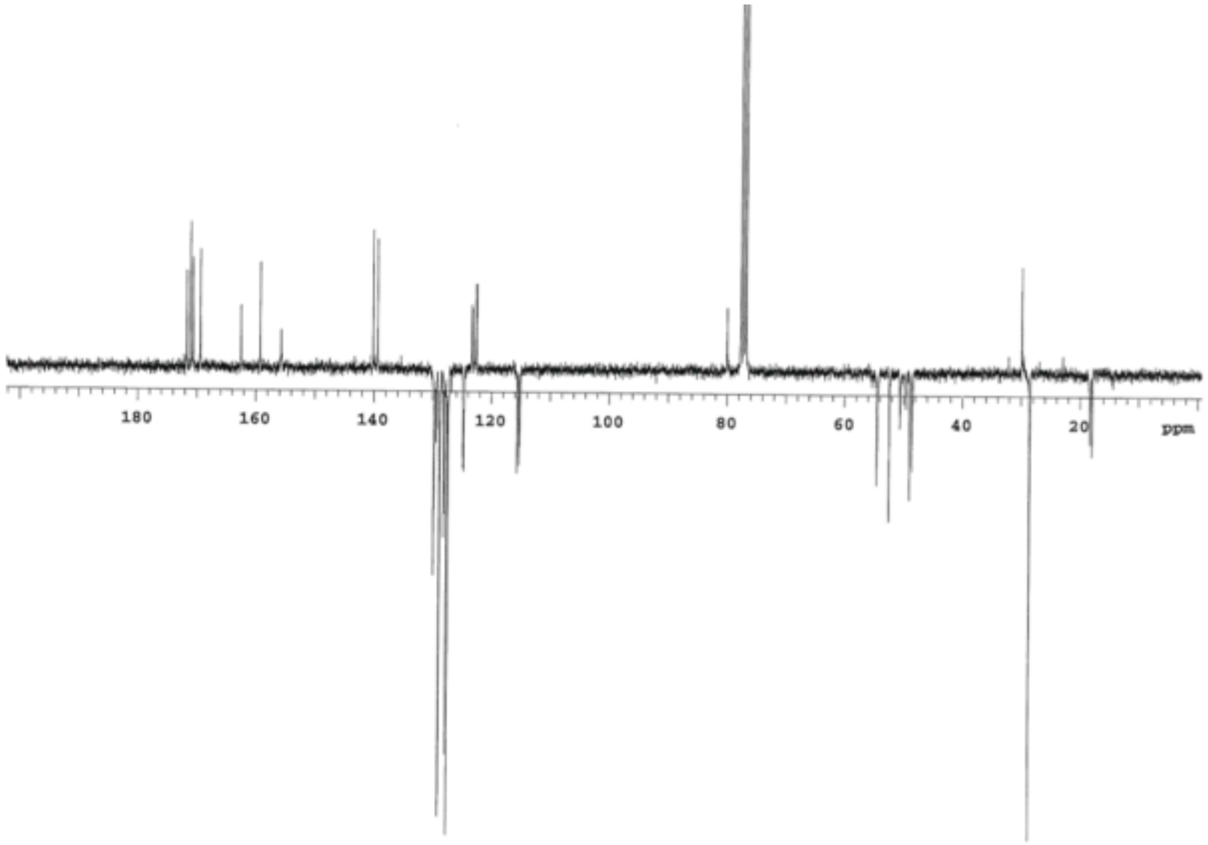

Figure S21: ^{13}C NMR spectra of Boc-[*S*-Ala-β–2*R,3R*-Fpg]$_2$-OMe (**7**)

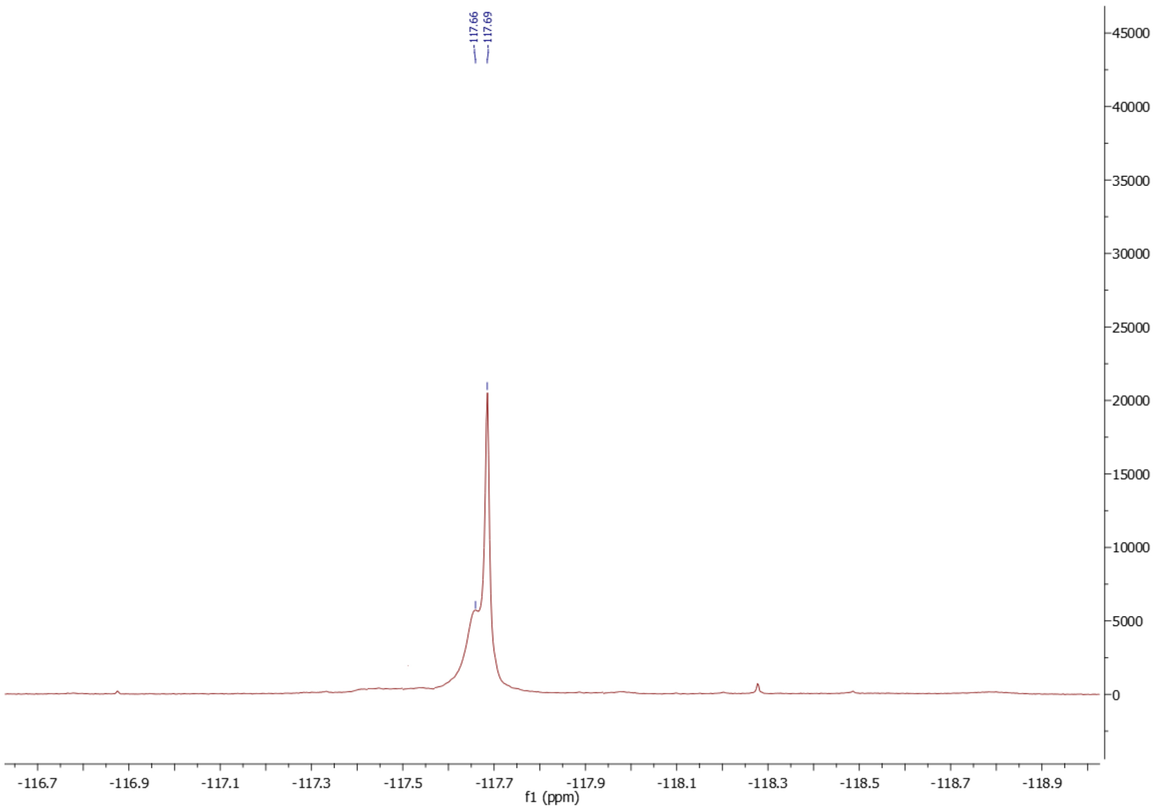

Figure S22: ^{19}F NMR spectra of Boc-[*S*-Ala-β−2*R,3R*-Fpg]$_2$-OMe (**7**)

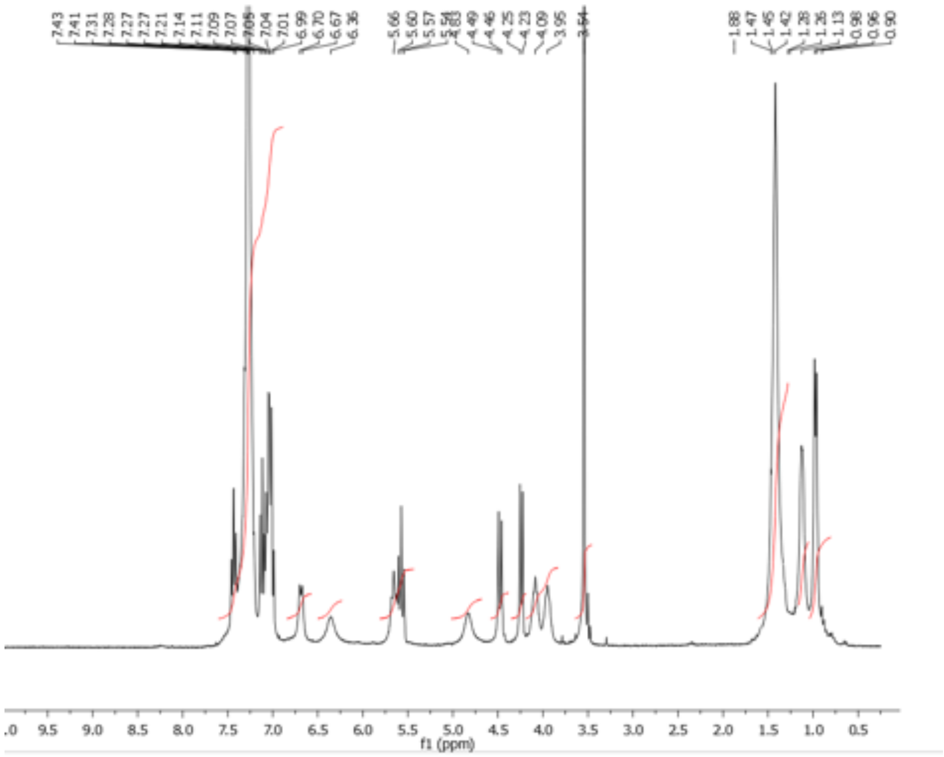

Figure S23: ^{1}H NMR spectra of Boc-[*S*-Ala-β−2*S,3S*-Fpg]$_2$-OMe (**8**)

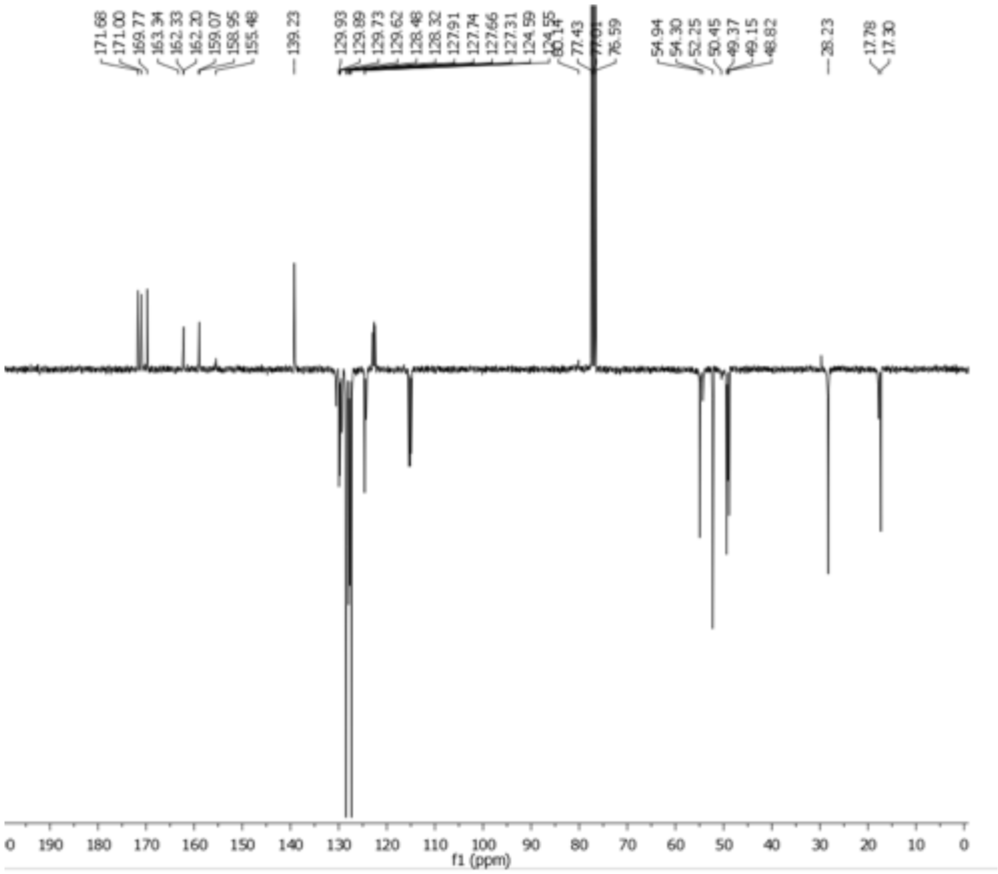

Figure S24: ^{13}C NMR spectra of Boc-[*S*-Ala-β–2*S,3S*-Fpg]$_2$-OMe (**8**)

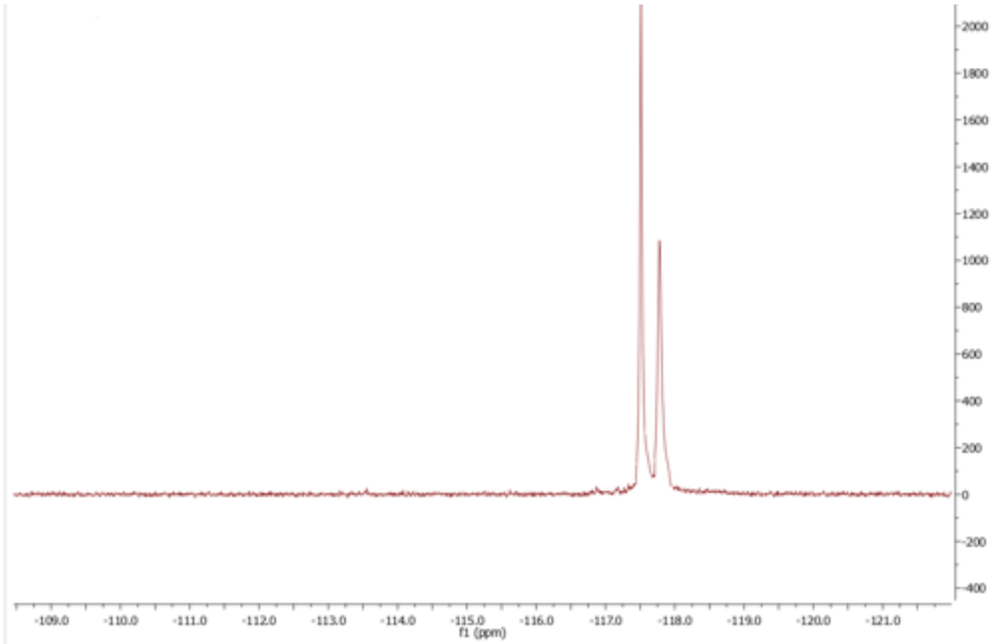

Figure S25: ^{19}F NMR spectra of Boc-[*S*-Ala-β–2*S,3S*-Fpg]$_2$-OMe (**8**)

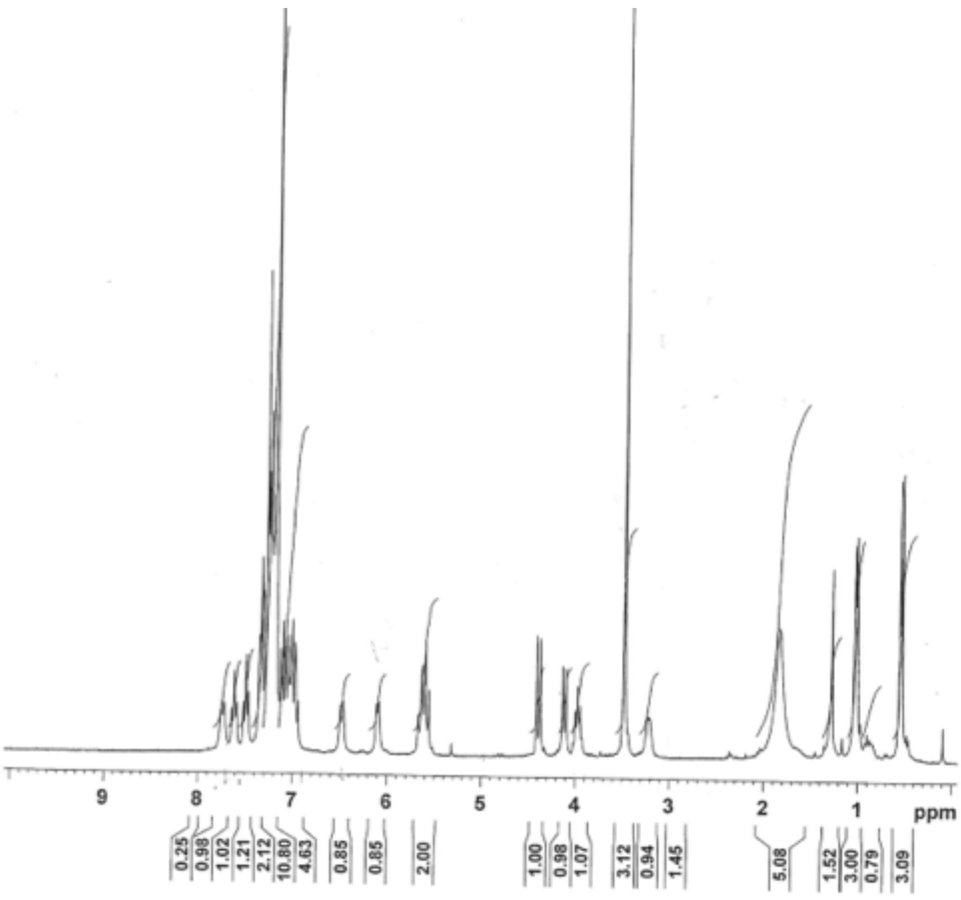

Figure S26: ^{1}H NMR spectra of H-[*S*-Ala-β–2*R,3R*-Fpg]$_2$-OMe (**9**)

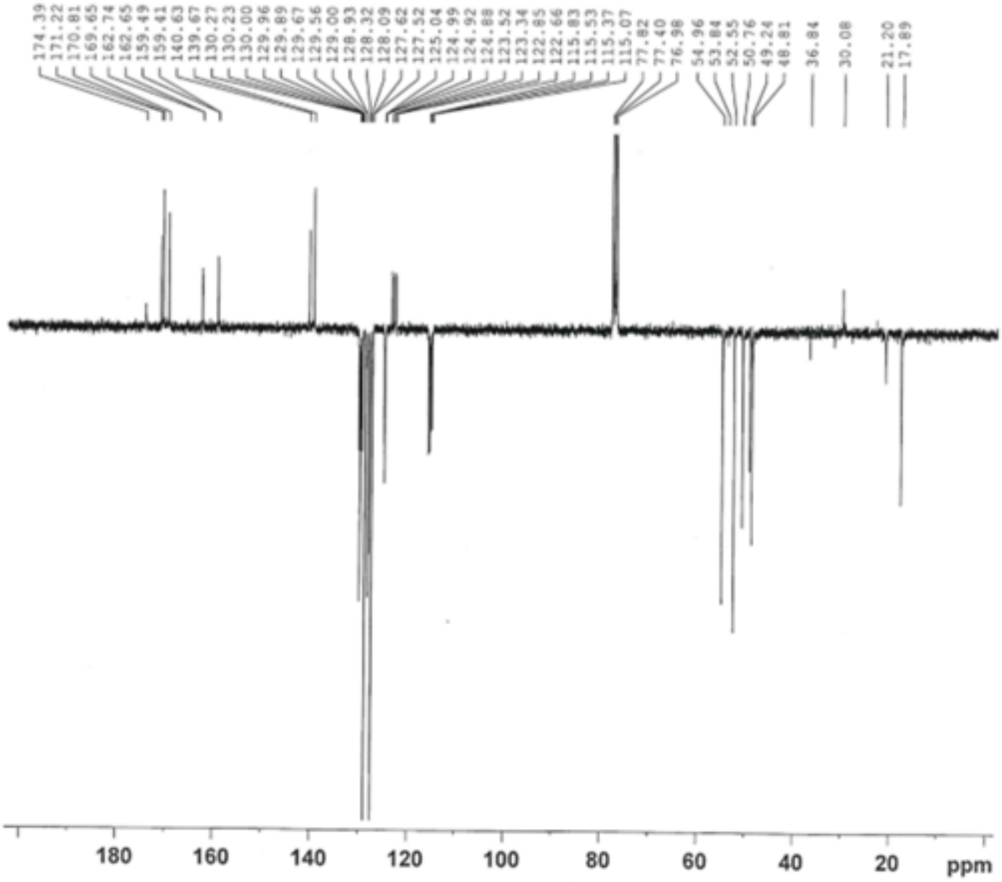

Figure S27: ^{13}C NMR spectra of H-[*S*-Ala-β−2*R,3R*-Fpg]$_2$-OMe (**9**)

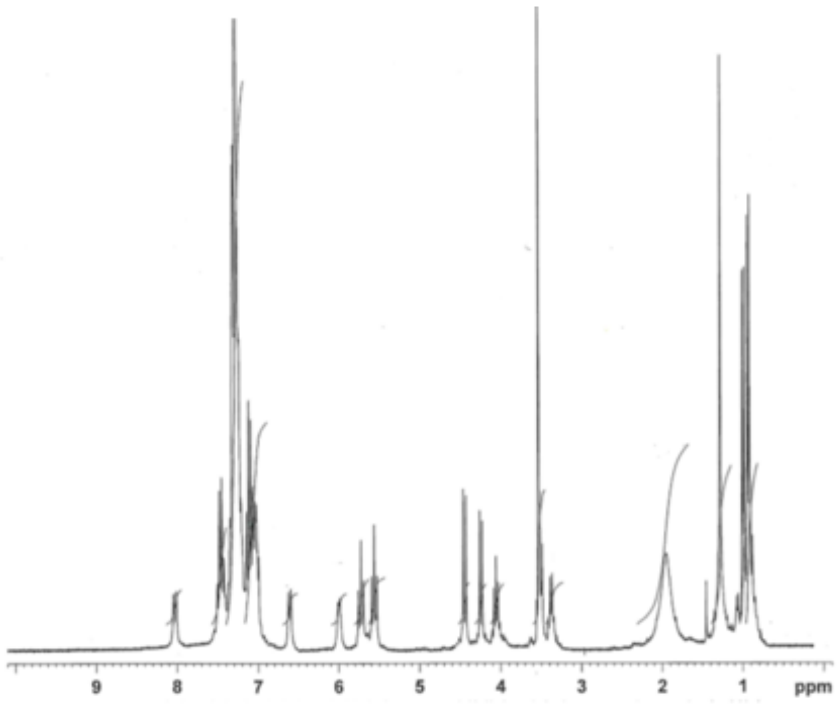

Figure S28: ^{1}H NMR spectra of H-[*S*-Ala-β−2*S,3S*-Fpg]$_2$-OMe (**10**)

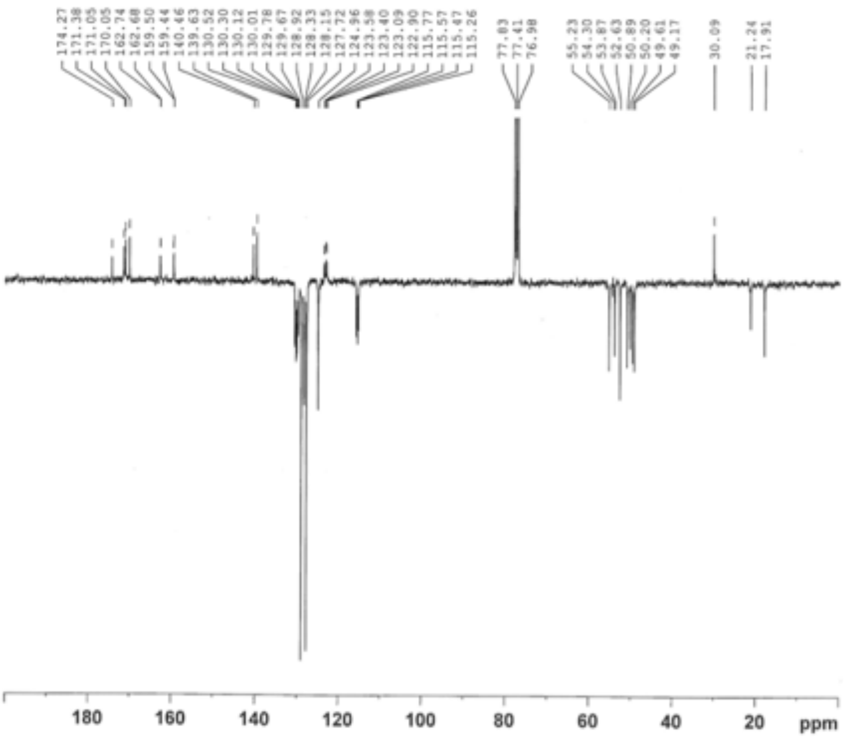

Figure S29: ^{13}C NMR spectra of H-[*S*-Ala-β−2*S*,3*S*-Fpg]$_2$-OMe (**10**)

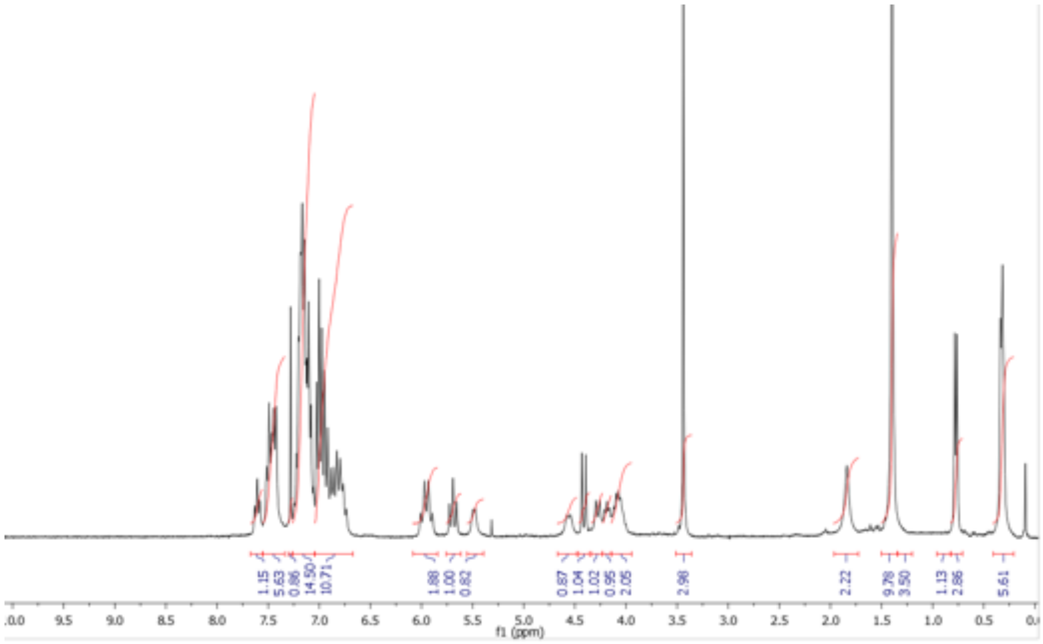

Figure S30: ^{1}H NMR spectra of Boc-[*S*-Ala-β−2*R*,3*R*-Fpg]$_3$-OMe (**11**) in $CDCl_3$

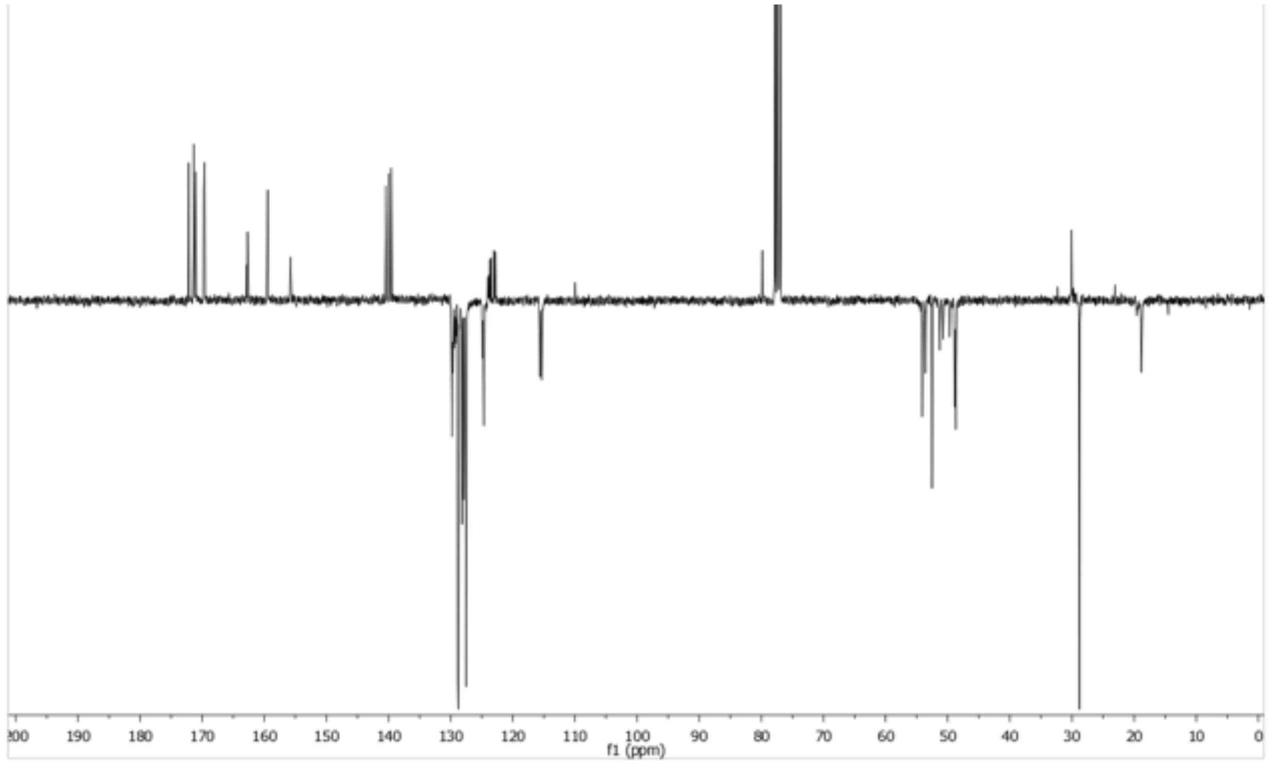

Figure S31: ^{13}C NMR spectra of Boc-[*S*-Ala-β−2*R,3R*-Fpg]$_3$-OMe (**11**) in $CDCl_3$

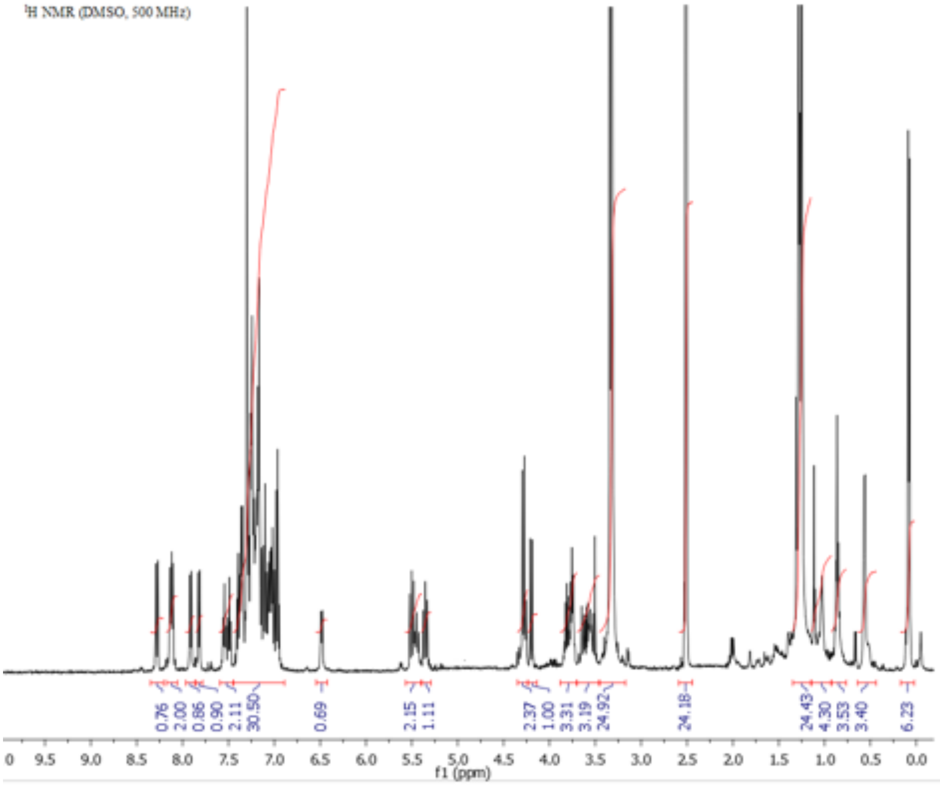

Figure S32: ^{1}H NMR spectra of Boc-[*S*-Ala-β−2*R,3R*-Fpg]$_3$-OMe (**11**) in DMSO

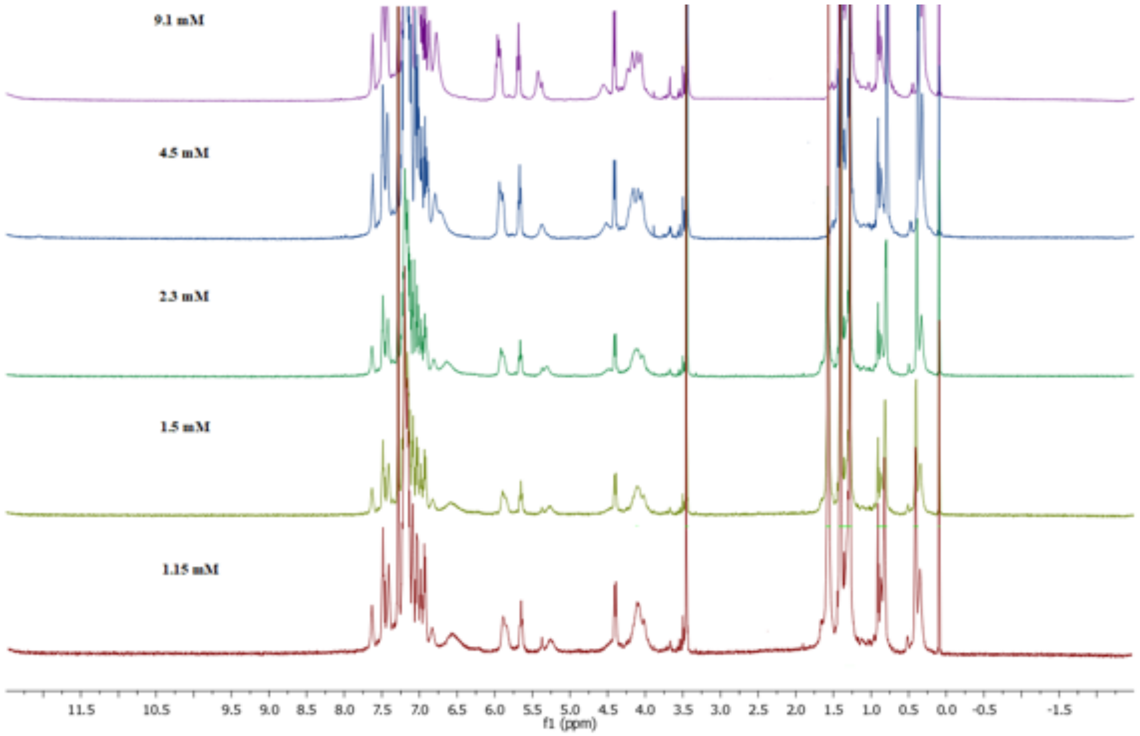

Figure S33: ^{1}H NMR spectra of Boc-[*S*-Ala-β−2*R,3R*-Fpg]$_3$-OMe (**11**) at different concentrations in $CDCl_3$ (1.15-9.10 mM)

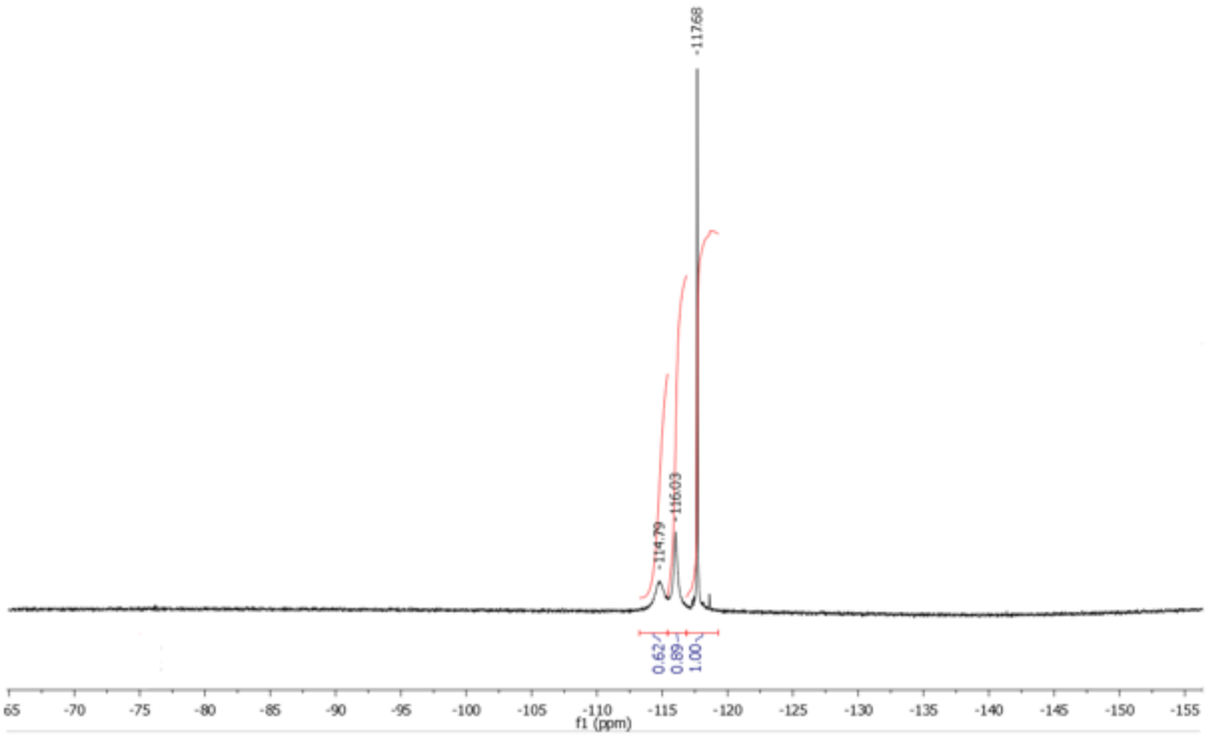

Figure S34: ^{19}F NMR spectra of Boc-[*S*-Ala-β−2*R,3R*-Fpg]$_3$-OMe (**11**)

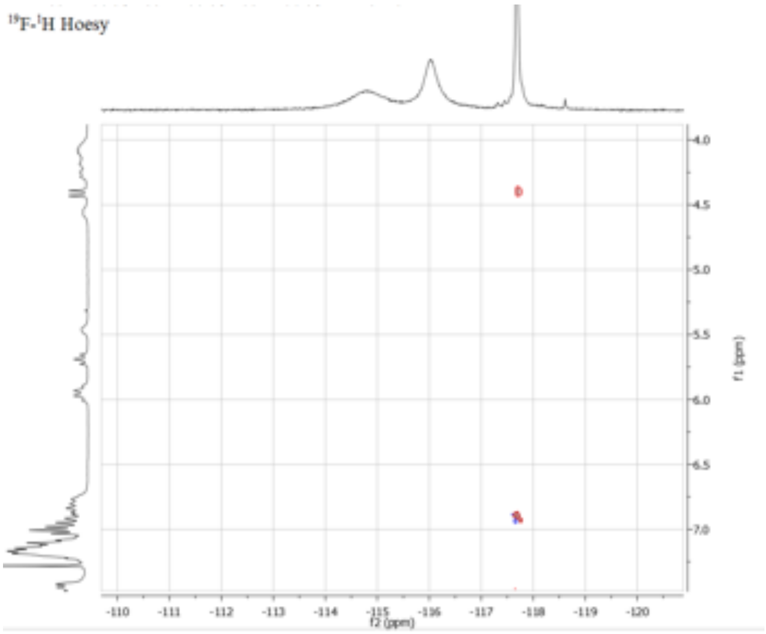

Figure S35: ^{19}F-^{1}H Hoesy spectra of Boc-[*S*-Ala-β–2*R*,3*R*-Fpg]$_3$-OMe (**11**)

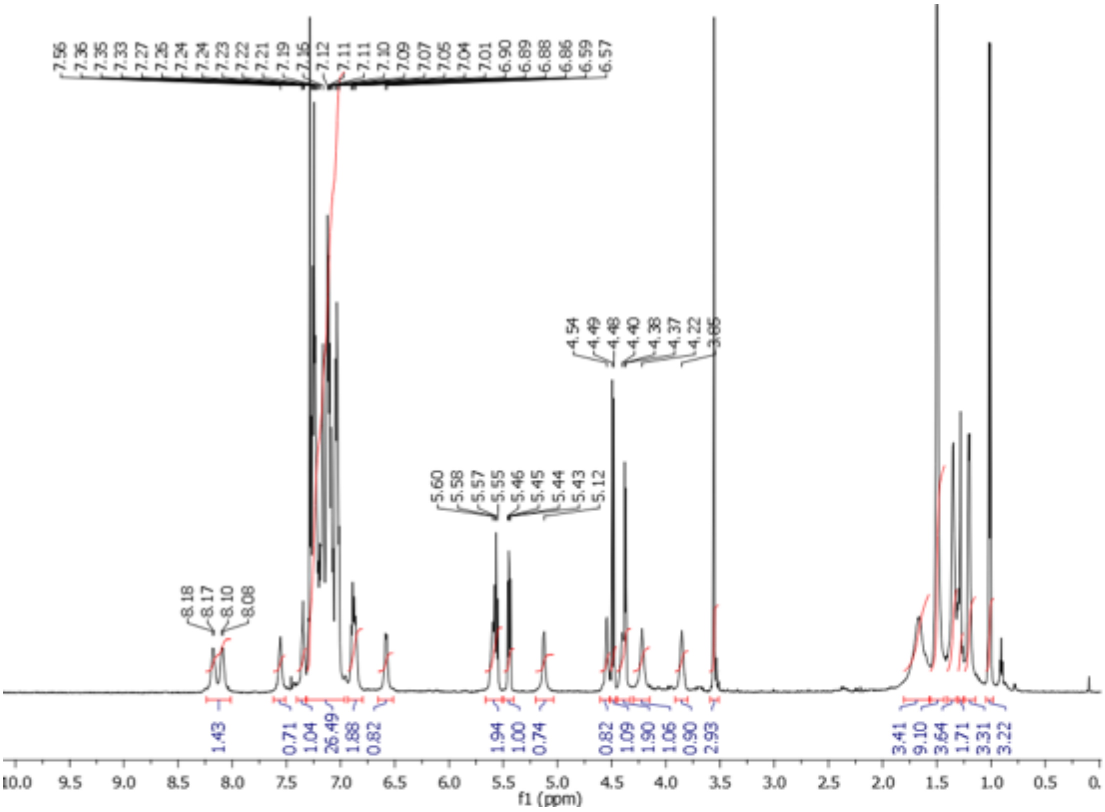

Figure S36: ^{1}H NMR spectra of Boc-[*S*-Ala-β–2*S*,3*S*-Fpg]$_3$-OMe (**12**)

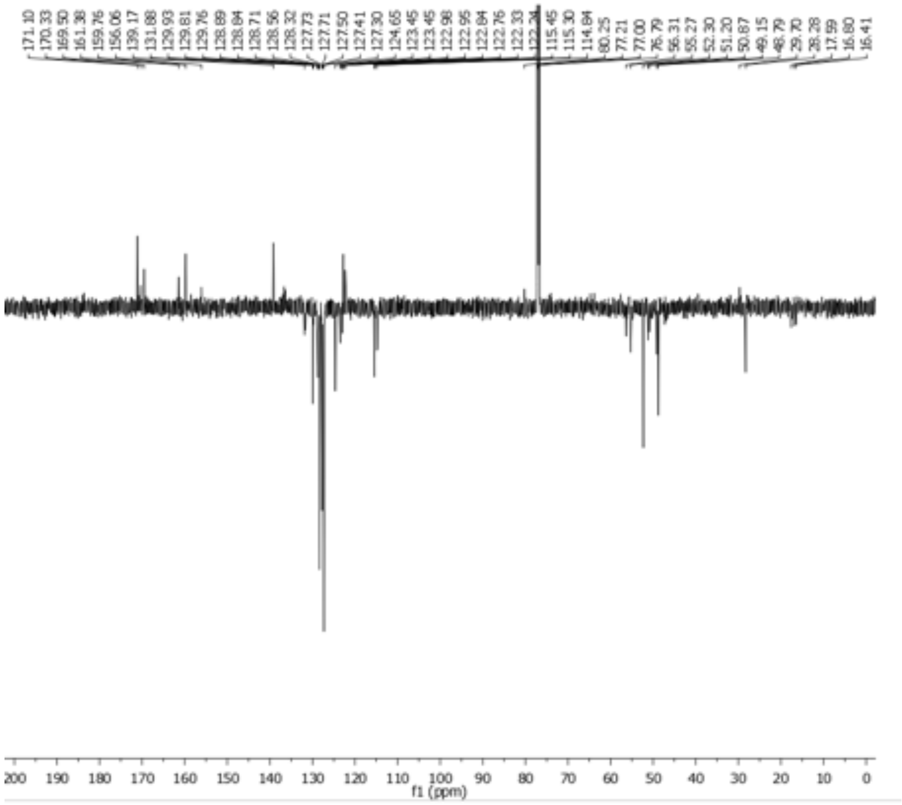

Figure S37: ^{13}C NMR spectra of Boc-[*S*-Ala-β−2*S,3S*-Fpg]$_3$-OMe (**12**)

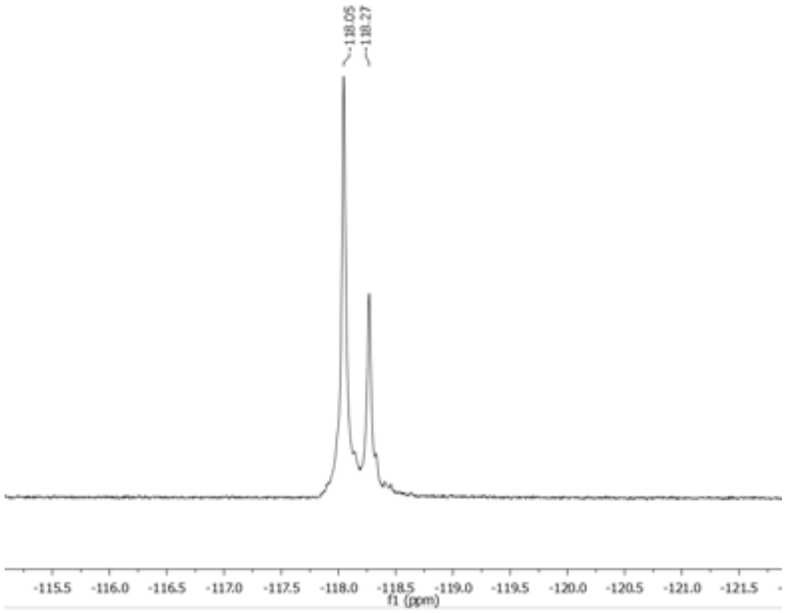

Figure S38: ^{19}F NMR spectra of Boc-[*S*-Ala-β−2*S,3S*-Fpg]$_3$-OMe (**12**)

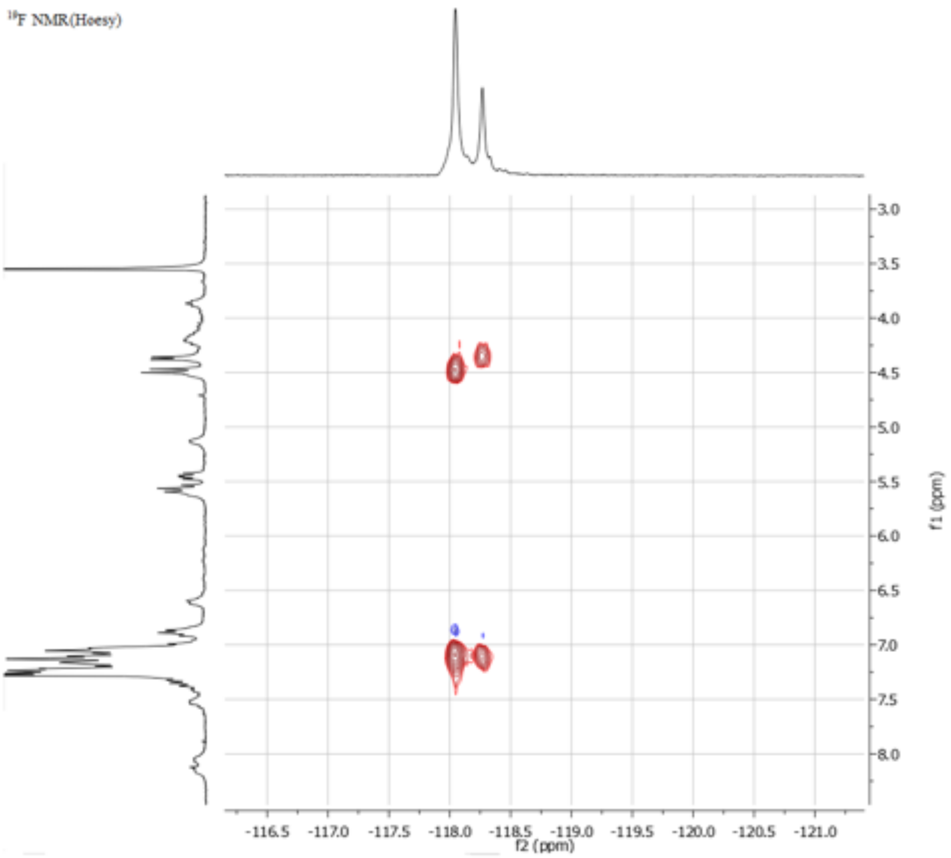

Figure S39: ^{19}F-^{1}H Hoesy spectra of Boc-[*S*-Ala-β–2*S*,*3S*-Fpg]$_3$-OMe (**12**)
